# Evolutionary origin of type IV classical cadherins in arthropods

**DOI:** 10.1186/s12862-017-0991-2

**Published:** 2017-06-17

**Authors:** Mizuki Sasaki, Yasuko Akiyama-Oda, Hiroki Oda

**Affiliations:** 1 Laboratory of Evolutionary Cell and Developmental Biology, JT Biohistory Research Hall, 1-1 Murasaki-cho, Takatsuki, 569-1125 Osaka Japan; 2 Department of Microbiology and Infection Control, Osaka Medical College, Takatsuki, Osaka Japan; 3 Department of Biological Sciences, Graduate School of Science, Osaka University, Osaka, Japan; 4 Current address: Department of Parasitology, Asahikawa Medical University, 2-1-1-1 Midorigaoka-higashi, Asahikawa, 078-8510 Hokkaido Japan

**Keywords:** Cadherin, Cell adhesion, Adherens junction, Arthropod, Chelicerate, Crustacean, Insect, Genome, Evolution, Phylogeny

## Abstract

**Background:**

Classical cadherins are a metazoan-specific family of homophilic cell-cell adhesion molecules that regulate morphogenesis. Type I and type IV cadherins in this family function at adherens junctions in the major epithelial tissues of vertebrates and insects, respectively, but they have distinct, relatively simple domain organizations that are thought to have evolved by independent reductive changes from an ancestral type III cadherin, which is larger than derived paralogs and has a complicated domain organization. Although both type III and type IV cadherins have been identified in hexapods and branchiopods, the process by which the type IV cadherin evolved is still largely unclear.

**Results:**

Through an analysis of arthropod genome sequences, we found that the only classical cadherin encoded in chelicerate genomes was the type III cadherin and that the two *type III cadherin* genes found in the spider *Parasteatoda tepidariorum* genome exhibited a complex yet ancestral exon-intron organization in arthropods. Genomic and transcriptomic data from branchiopod, copepod, isopod, amphipod, and decapod crustaceans led us to redefine the type IV cadherin category, which we separated into type IVa and type IVb, which displayed a similar domain organization, except type IVb cadherins have a larger number of extracellular cadherin (EC) domains than do type IVa cadherins (nine versus seven). We also showed that *type IVa cadherin* genes occurred in the hexapod, branchiopod, and copepod genomes whereas only *type IVb cadherin* genes were present in malacostracans. Furthermore, comparative characterization of the type IVb cadherins suggested that the presence of two extra EC domains in their N-terminal regions represented primitive characteristics. In addition, we identified an evolutionary loss of two highly conserved cysteine residues among the type IVa cadherins of insects.

**Conclusions:**

We provide a genomic perspective of the evolution of classical cadherins among bilaterians, with a focus on the Arthropoda, and suggest that following the divergence of early arthropods, the precursor of the insect type IV cadherin evolved through stepwise reductive changes from the ancestral type III state. In addition, the complementary distributions of polarized genomic characters related to type IVa/IVb cadherins may have implications for our interpretations of pancrustacean phylogeny.

**Electronic supplementary material:**

The online version of this article (doi:10.1186/s12862-017-0991-2) contains supplementary material, which is available to authorized users.

## Background

Classical cadherins, a metazoan-specific subfamily of the cadherin superfamily [1–3], are homophilic cell-cell adhesion molecules that play key roles in metazoan morphogenesis [3–7], and as single-pass transmembrane proteins, their ectodomains contain repetitive extracellular cadherin (EC) domains that function to recognize and bind cells that express the same or similar cadherin molecules [8, 9]. The cytoplasmic domains of classical cadherins also bind to catenins [10], through which they interact with the actomyosin network [7] and potentially integrate actomyosin-generated physical forces into tissue-level tension, thereby regulating tissue homeostasis and morphogenesis [11–13].

Genes that encode classical cadherins have been identified in many bilaterian species, as well as in several non-bilaterian metazoans [2, 3, 14–19], and studies in both vertebrate and insect models have firmly established the role and mechanisms of classical cadherins in animal development [4, 5, 7]. However, despite the conservation of their functions, classical cadherins exhibit remarkable variation in the structure of their ectodomains [3], and members of the classical cadherin family have been categorized as types I, II, III, and IV, or otherwise, based on their phylogenetic grouping and domain organization [1, 3, 20].

**Fig. 1:**
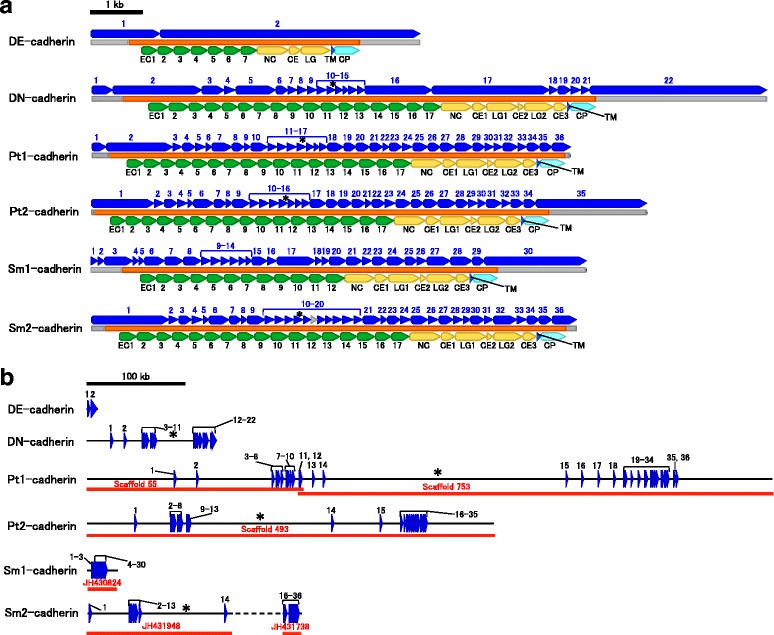
Genomic and domain organization of classical cadherins in *Parasteatoda tepidariorum and Strigamia maritima*. **a**. Schematic representation of exons (upper), transcripts (middle), and domain organization (lower) of Pt1-, Pt2-, Sm1-, and Sm2- cadherins, compared with those of DE- and DN-cadherins. The scale bar indicates 1 Kbp. All exons identified in the genome sequences are depicted in blue, except for the exons depicted in gray for the *Sm2-cadherin* gene, which remained hypothetical because its sequence was not found in the *S. maritima* genome sequence assembly. For each cadherin, the exons are tentatively numbered to facilitate comparison (numbers in blue). The coding region of each transcript is depicted in orange, with the 5’- and 3’-untranslated regions depicted in gray. The domain names are abbreviated as follows: EC, extracellular cadherin domain; NC, non-chordate classical cadherin domain; CE, cysteine-rich EGF-like domain; LG, laminin globular-like domain; TM, transmembrane domain; CP, cytoplasmic domain. **b**. Schematic representation of the genomic organization of the *Pt1-*, *Pt2-*, *Sm1-*, and *Sm2-cadherin* genes, compared with those of the *DN-* and *DE-cadherin* genes. Thin horizontal black lines indicate the genome sequences. The scale bar indicates 100 Kbp. Blue triangles indicate individual exons, which are numbered to facilitate comparison. Red lines indicate scaffold sequences of the *P. tepidariorum* or *S. maritima* genome assemblies. Broken lines indicate missing sequences. Conserved insertions of the largest introns observed in the *Pt1-*, *Pt2-*, *DN-*, and *Sm2-cadherin* genes are indicated by asterisks in both A and B. The genomic sequences annotated for the Pt1- and Pt2-cadherins are available in GenBank (BR001342 and BR001343)

Type I and type II cadherins each possess five tandem EC domains, and these cadherin types are common in vertebrates but have not been reported to occur in invertebrates, with the exception of urochordates [14, 21]. Certain subtypes of type I and type II cadherins, including E-cadherin (type I) and cadherin-5 or VE-cadherin (type II), serve as components of adherens junctions in vertebrate epithelial tissues. However, type IV cadherins function as the key adhesion molecules of adherens junctions in insect epithelial tissues and include the *Drosophila melanogaster* E-cadherin, DE-cadherin (Fig. 1a), which is the representative type IV cadherin [22–24]. Type IV cadherins are characterized by their shared domain organization, which includes seven EC domains, followed by the non-chordate classical cadherin (NC), cysteine-rich EGF-like (CE), and laminin-G (LG) domains [25], and they have been identified in insects, non-insect hexapods (e.g., collembolan) and branchiopod crustaceans [15]. Importantly, recent studies have revealed that the structural mechanisms responsible for homophilic binding of type I/II and type IV cadherins are quite different [26, 27]. Moreover, type III cadherins are distributed among a wide range of bilaterian metazoans, including arthropods, echinoderms, and even vertebrates, but they have yet to be identified in non-bilaterian metazoans [2, 15, 20, 28, 29]. The representative type III cadherin is *D. melanogaster* neural cadherin, DN-cadherin (Fig. 1a), the expression and function of which primarily occurs in non-epithelial tissues [30]. In contrast to type I, II, and IV cadherins, type III cadherin molecules contain 14 to 17 EC domains followed by the ectodomain, which includes one NC, three CE (CE1-CE3), and two LG (LG1 and LG2) domains with the following organization: NC-CE1-LG1-CE2-LG2-CE3. In addition, non-categorized/unconventional forms of classical cadherins have also been reported to occur in nematodes, hemichordates, and cephalochordates [15, 31, 32]. Although up to 17 EC domains have been observed in the classical cadherins of bilaterians, 25 or more have been reported in the classical cadherin-encoding genes of non-bilaterian metazoans [2, 17].

The structural variation of the ectodomains of classical cadherins is thought to have resulted from domain losses that occurred at critical points in metazoan or bilaterian evolution [2, 3, 15, 33]. This hypothesis is based on the conclusion that the type III form represents the last common precursor of all bilaterian classical cadherins, a conclusion that is supported by the widespread, albeit scattered, phylogenetic distribution of *type III cadherin* genes among bilaterians, detectable conservation throughout the amino acid sequences of type III cadherins, and the observation that all other forms of cadherins can be recognized as derived states of the type III form [15, 34]. However, it remains unclear whether the various forms of classical cadherins were present in the last common ancestors of the individual phyla, as well as whether the currently recognized derived states, i.e., the type I/II and type IV cadherins, evolved from the ancestral type III state during a distinct event or through progressive evolution. Efforts to answer these questions may contribute to a better understanding of how the structural mechanisms of classical cadherin-mediated adhesion evolved in metazoans and at what points of animal evolution the adhesion mechanisms were changed or modified.

To address these questions, we focused on the phylum Arthropoda, in which growing volumes of genomic and transcriptomic sequence resources are available for a broad range of species. We investigated both genomic and transcriptomic classical cadherin-encoding sequences from a wide range of arthropod and non-arthropod bilaterians, including chelicerates, a myriapod, and several non-branchiopod crustaceans, to determine whether *type IV cadherin* genes evolved from *type III cadherin* genes before, during, or after the early divergence of arthropods, and whether type IV cadherins arose from the type III state abruptly or through an intermediate state (or several intermediate states).

## Results

### *Classical cadherin* genes in the chelicerate *P. tepidariorum* genome

In the present study, we first identified a *P. tepidariorum* (common house spider formerly known as *Achaearanea tepidariorum*) type III cadherin-encoding cDNA (Fig. 1a) that was distinct from a copy of At-cadherin cDNA previously reported [15]. Therefore, the previously identified At-cadherin was redesignated Pt1-cadherin, and the newly identified gene product was designated Pt2-cadherin.

RNA sequencing (RNA-seq) of *P. tepidariorum* embryos at stages 5 and 10 demonstrated that the Pt1- and Pt2-cadherin transcripts were expressed in both early and late embryonic stages and that, at both stages, the expression level of the *Pt2-cadherin* gene was greater than that of the *Pt1-cadherin* gene (Additional file 1: Tables S1, S2). The predicted Pt1- and Pt2-cadherins were 2985 and 2961 amino acids long, respectively, and the sequences could be aligned along their entire lengths, exhibiting 66% identity. These sequences could also be aligned with the DN-cadherin sequence; however, the N-terminal regions appeared to have diverged. Using a protein domain search of the PROSITE database [35], we detected 17 and 16 EC domains in the Pt1- and Pt2-cadherin sequences, respectively, and two LG domains in each. Next, we aligned the sequences of the EC repeats (Additional file 2: Figure S1) and defined the start and end positions of the individual EC domains, which were numbered from 1 to 17 (EC1 to EC17). The more C-terminal regions of the Pt1- and Pt2-cadherins were subdivided into eight domains (NC, CE1, LG1, CE2, LG2, CE3, TM, and CP; Fig. 1a; Additional file 2; Figure S1). Although some NC domain sequences in classical cadherins have been reported to exhibit weak similarities to typical EC domains [36], the NC domain was not considered an EC domain in this work because of its limited sequence similarity. Practically, the positions of the domains of DN-cadherin and other type III cadherins were defined based on sequence alignment with the Pt1- and Pt2-cadherins.

To investigate the genomic organization of the *Pt1-* and *Pt2-cadherin* genes, we used scaffold sequences of the *P. tepidariorum* isolate Göttingen genome (~1.4 Gbp) (GCA_000365465.1) [37], as well as whole genome shotgun sequencing (WGS) reads of the *P. tepidariorum* isolate Osaka genome (>31× coverage) (Additional file 1: Table S3). The full-length nucleotide sequence of the Pt1- cadherin cDNA was mapped to the ~488 Kbp region of Scaffolds 55 and 753, which could be connected into a continuous sequence (Fig. 1b) and contained at least 35 exons that were separated by introns of various sizes (from 444 bp to more than 240 Kbp). Similarly, the full-length nucleotide sequence of the *Pt2-cadherin* transcript was mapped to the ~298 Kbp region of Scaffold 493 (Fig. 1b), and we found that the *Pt2-cadherin* gene contained at least 36 exons that were separated by introns of various sizes (from 75 bp to more than 140 Kbp). Most, but not all, of the exons in both genes were small (<400 bp), and all of the introns in the protein-coding regions of the *Pt1-* and *Pt2- cadherin* genes were inserted at homologous sites (Additional file 2: Figure S1). In addition, the total lengths of the *Pt1-* and *Pt2-cadherin* genes were much larger than the total length of the *DN-cadherin* gene (Fig. 1a, b); however, the three genes shared at least 13 intron insertion sites, including those for the largest introns (Fig. 1a, b; Additional file 2: Figure S1).

To investigate whether a third *classical cadherin* gene was present in the *P. tepidariorum* genome, we exhaustively searched the genome *P. tepidariorum* isolate Göttingen genome sequence assembly and reads from the *P. tepidariorum* isolate Osaka WGS and RNA-seq. However, there was no sign of a third *classical cadherin* gene in *P. tepidariorum*, which led us to conclude that the *Pt1-* and *Pt2-cadherin* genes are the only *classical cadherin* genes in the species.

### Identification of *classical cadherin* genes in other non-hexapod arthropod genomes

To investigate the repertoire of *classical cadherin* genes in other non-hexapod arthropod genomes, we searched the publicly available genome sequence assemblies of four chelicerate species (velvet spider *Stegodyphus mimosarum* [38]; two-spotted mite *Tetranychus urticae* [39]; western predatory mite *Metaseiulus occidentalis* [40]; *Mesobuthus martensii* [41]), a myriapod species (centipede *Strigamia maritima* [42]), and four crustacean species (water flea *Daphnia pulex* [43]; copepod *Eurytemora affinis* [37]; amphipod *Hyalella azteca* [37]; amphipod *Parhyale hawaiensis* [44]) (Table 1). The capability to detect the entire organization of *classical cadherin* genes depended on the quality of the genome sequence assembly and the availability of rich transcriptomic resources. The genome sequence assemblies that were searched comprised scaffolds or contigs with relatively high N50 values and relatively low proportions of undetermined bases. Although transcript models for classical cadherins were predicted in many of the genome sequence assemblies, we carefully evaluated the organization of all detectable *classical cadherin* genes. RNA-seq reads, if publicly available and necessary, were used to reconstruct the transcript sequence of classical cadherins. In addition to the publicly available sequence resources, we generated RNA-seq reads for both sea slater *Ligia exotica* and freshwater shrimp *Caridina multidentata*, as well as WGS reads with approximately 8× and 13× coverage depths, respectively (Table 1; Additional file 1: Tables S1, S3). These sequence resources were also used to search for *classical cadherin* genes.

**Table 1:** *Classical cadherin* genes found in publicly available genome sequences of non-hexapod arthropods

Taxon/species	Genome accession	#Scaffold (Gene accession)	Type	Product
Chelicerata, Araneae				
*Parasteatoda tepidariorum*	GCA_000365465.1	#55/#753 (AB190303)	III	Pt1-cadherin
		#493 (LC110189)	III	Pt2-cadherin
*Stegodyphus mimosarum*	GCA_000611955.2	#7/#4105/#15,197/#13303^a^	III	(Close to Pt1)
		#10064/#11,847/#1110^a^	III	(Close to Pt2)
Chelicerata, Scorpiones				
*Mesobuthus martensii*	GCA_000484575.1	#343080^b, c, d^	III	Mma1-cadherin
		#352483^b, c, d^	III	Mma2-cadherin
Chelicerata, Acari				
*Tetranychus urticae*	GCA_000239435.1	#8 (XP_015784984)	III	
*Metaseiulus occidentalis*	GCA_000255335.1	#JH621154 (XM_003743492)	III	Mo-cadherin
Myriapoda, Chilopod				
*Strigamia maritima*	GCA_000239455.1	#JH431948/#JH431738^d^	III	Sm2-cadherin
		#JH430824^d^ (SMAR001807)	n.c.	Sm1-cadherin
Crustacea, Branchiopoda				
*Daphnia pulex*	GCA_000187875.1	#100 (EFX70325)	III	Dp2-cadherin
		#3 (EFX89066)	IVa	Dp1-cadherin
Crustacea, Copepoda				
*Eurytemora affinis*	GCA_000591075.1	#33^d^	III	Ea2-cadherin
		#103273/#511^d^	IVa	Ea1-cadherin
Crustacea, Isopoda				
*Ligia exotica*	BDMT010000000	(AB190302)	III	Le2-cadherin
		(LC110190)	IVb	Le1-cadherin
Crustacea, Amphipoda				
*Hyalella azteca*	GCA_000764305.2	#323 (XM_018161032)	III	Ha2-cadherin
		#236 (XM_018157906)	IVb	Ha1-cadherin
*Parhyale hawaiensis*	GCA_001587735.1	#25754^c, e^ (tra_m.010273)	III	Ph2-cadherin
		#4723 (tra_m.024063)	IVb	Ph1-cadherin
Crustacea, Decapoda				
*Caridina multidentata*	BDMR010000000	(AB190301)	III	Cm-cadherin

^a^The scaffolds were linked by detected sequences that were very similar to those of Pt1- or Pt2-cadherin

^b^#Contig

^c^Only the scaffold or contig containing exons coding for the CP domain is shown

^d^Sequence details are available in Additional files 11 and 12

^e^#95284 was detected as a partial duplicate. n.c., not categorized

#### Chelicerates

In each of the *S. mimosarum and M. martensii* genomes, we detected two *type III cadherin* genes that were closely related to the *Pt1-* and *Pt2-cadherin* genes. In the genomes of both *T. urticae* and *M. occidentalis*, we detected a single *type III cadherin* gene; however, no other *classical cadherin* genes were detected. In addition, we found that all intron insertion sites in the coding regions of all the *type III cadherin* genes of *P. tepidariorum* and *M. martensii* were conserved between them (Additional file 3: Figure S2).

#### Myriapods

In the *S. maritima* genome sequence assembly, two *classical cadherin* genes were identified (Fig. 1a; Table 1). The predicted products were designated Sm1- and Sm2-cadherin. The Sm2-cadherin was considered a type III cadherin, and its exon-intron structure was similar, but not identical, to that of the *Pt1-* and *Pt2-cadherin* genes, although a small portion of the coding sequence was not mapped to any scaffold (Fig. 1a, b). In contrast, Sm1-cadherin exhibited most of the typical type III cadherin elements, but since it contained only 12 EC domains, it could be classified as neither a type III nor a type IV cadherin. In addition, we also observed that the *Sm1-cadherin* gene contained at least 30 exons that were condensed within a small genomic region (~15 Kbp) (Fig. 1a, b).

#### Branchiopod crustaceans

In the branchiopod crustacean *Artemia franciscana*, we previously identified both type IV and type III cadherins*,* i.e., Af1- and Af2-cadherin [15], which were orthologous to DE- and DN-cadherin, respectively, as well as to two predicted products from the *Daphnia pulex* genome [43], hereafter referred to as Dp1- and Dp2-cadherin (Table 1).

#### Non-branchiopod crustaceans

**Fig. 2:**
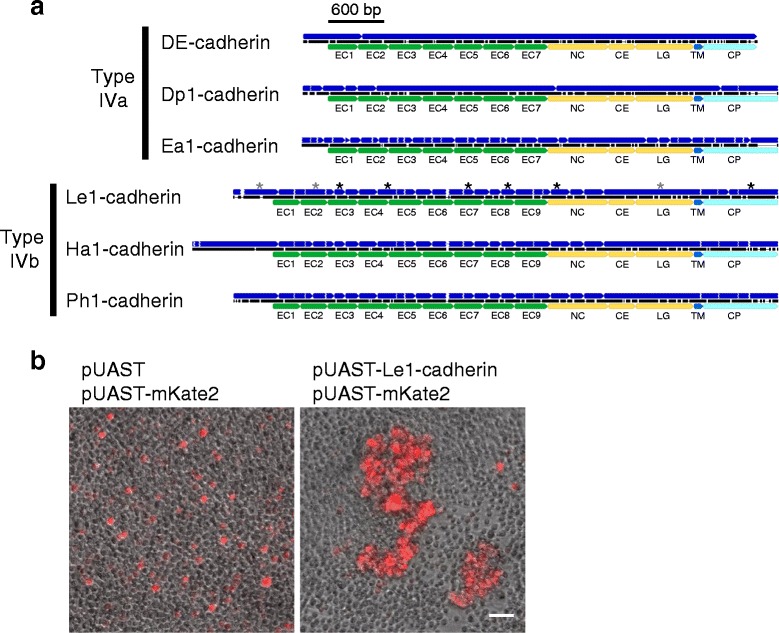
Reconstruction of transcripts for type IVa and type IVb cadherins in pancrustaceans. **a**. Schematic representation of exons (*upper, blue*), coding sequences (*middle, black*), and domain organization (*lower*) of the type IVa DE-, Dp1-, and Ea1-cadherins from *D. melanogaster*, *D. pulex*, and *E. affinis*, respectively, and the type IVb Le1-, Ha1-, and Ph1-cadherins from *L. exotica*, *H. azteca*, and *P. hawaiensis*, respectively. The scale bar indicates 600 bp. Breaks in the black lines indicate gaps in the amino acid sequence alignment of the five cadherins (Additional file 4: Figure S3). The domain names are abbreviated as follows: EC, extracellular cadherin domain; NC, non-chordate classical cadherin domain; CE, cysteine-rich EGF-like domain; LG, laminin globular-like domain; TM, transmembrane domain; CP, cytoplasmic domain. Asterisks indicate transcript regions with sequences that were not (*black*) or only partially (*gray*) found in the WGS reads. **b**. Capability of Le1-cadherin to mediate cell aggregation. *Drosophila* S2 cells transiently transfected with empty pUAST (*left*) or pUAST-Le1-cadherin (*right*) in combination with pUAST-mKate2 and pWA-GAL4 were used for the cell aggregation assay. Cells expressing the exogenous genes were identified via mKate2 fluorescence (*red*). Scale bar, 50 μm

In the isopod *L. exotica* and the decapod *C. multidentata*, we previously identified type III cadherins but failed to detect any other forms [15]. In the present study using *L. exotica*, we were able to predict a transcript that encoded a hypothetical classical cadherin that was distinct from the previously identified Le-cadherin (Table 1; Fig. 2a), and the occurrence of the transcript was validated using reverse transcriptase polymerase chain reaction (PCR) amplification and sequencing. Accordingly, the newly identified classical cadherin was designated Le1-cadherin, and the previously identified Le-cadherin was redesignated Le2-cadherin. Notably, Le1-cadherin was structurally similar to type IV cadherins in that it lacked the CE2, LG2, and CE3 domains that are typical of type III cadherins (Fig. 2a; Additional file 4: Figure S3). However, the protein was distinct from other known type IV cadherins in that it contained two additional EC domains.

Considering that the N-terminal-most four EC domains of type IV DE-cadherin has a folded, globular structure involved in homophilic binding [26, 27], the finding of the unique domain organization of Le1-cadherin raised the question of whether it is functional. To investigate this question, we performed cell aggregation assays using *Drosophila* S2 cells transiently transfected with or without a Le1-cadherin expression construct (Fig. 2b). The result indicated that Le1-cadherin was capable of mediating cell-cell adhesion.

A *classical cadherin* gene specifically related to the *Le1-cadherin* gene was also identified in each of the *H. azteca* and *P. hawaiensis* genomes. Both these predicted products had essentially the same domain organization as Le1-cadherin, and they were designated Ha1- and Ph1-cadherin, respectively, (Fig. 2a; Additional file 4: Figure S3). The *Le1-*, *Ha1-* and *Ph1-cadherin* genes exhibited a relatively complex yet mutually similar exon-intron organization (Fig. 2a). As expected, the amphipod genomes also contained *type III cadherin* genes, and their predicted products were designated Ha2- and Ph2- cadherin (Additional file 3: Figure S2).

Additionally, analysis of the *C. multidentata* WGS reads revealed genomic regions with sequences that were distinct from the previously reported *type III Cm-cadherin* gene and were more closely related to Le1-cadherin than to Le2-cadherin (Additional file 5: Figure S4). However, since the *C. multidentata* RNA-seq data poorly represented sequences specifically related to Le1-cadherin, we were unable to generate a predicted transcript. The de novo assembly of the *C. multidentata* RNA-seq reads, nonetheless, allowed us to detect contigs encoding a part of classical cadherin closely similar but not identical to Cm-cadherin (Additional file 5: Figure S4). These contigs were connected by some raw reads, indicating that the *C. multidentata* genome might have another *classical cadherin* gene, which had retained domain elements characteristic of type III cadherin, rather than type IV cadherin.

In the genome sequence assembly of the copepod *E. affinis*, two hypothetical *classical cadherin* genes were detected, and their predicted products were designated Ea1- and Ea2-cadherin (Table 1). Ea2-cadherin was a type III cadherin, whereas Ea1-cadherin was a type IV cadherin that had essentially the same domain organization as those of DE- and Dp1-cadherin (Fig. 2a; Additional file 4: Figure S3).

### Redefinition of the type IV cadherin category

For simplicity and convenience, we redefined the term “type IV cadherin.” Irrespective of the total number of EC domains, all the classical cadherins that were characterized by the absence of the CE2, LG2, and CE3 domains were included in the type IV category, and the category was also separated into two subclasses, type IVa and type IVb, based on differences in their EC domains. More specifically, type IVa cadherins were defined as type IV cadherins that contain seven EC domains, whereas type IVb cadherins were defined as type IV cadherins that contain the same seven EC domains as well as two more EC domains (Fig. 2a). The validity of this classification will be further examined below.

### Relationships among the domain organizations of type II, III, IVa, and IVb cadherins

**Fig. 3:**
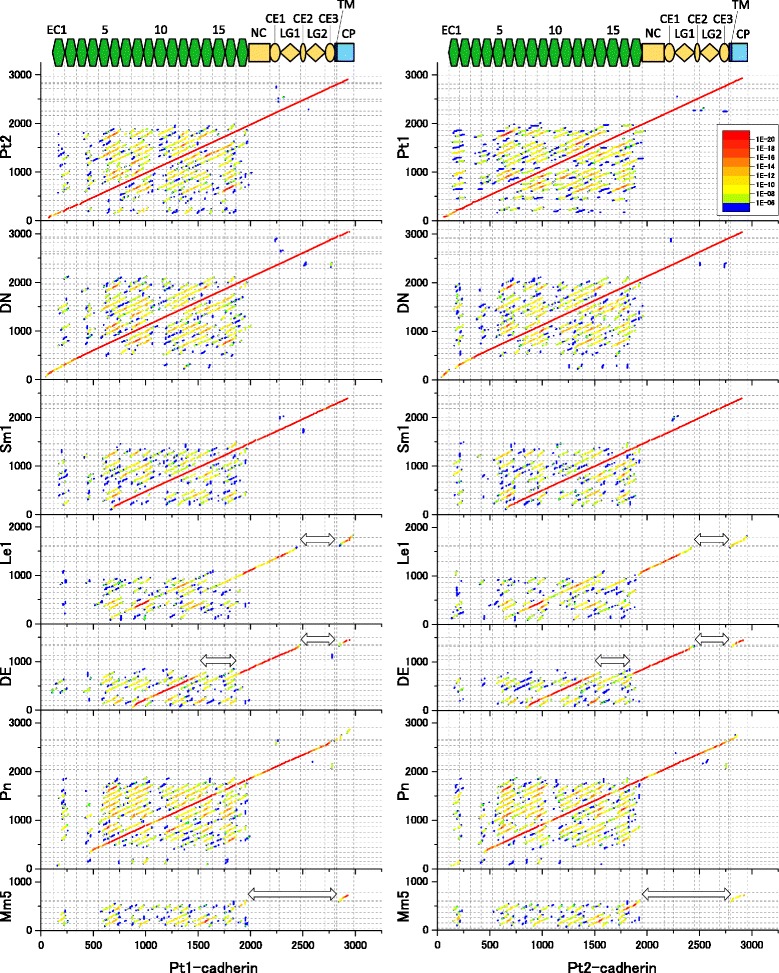
Blast-based dot-plot comparisons of classical cadherin amino acid sequences. The classical cadherins analyzed are as follows: type III Pt1-cadherin (Pt1, spider); type III Pt2-cadherin (Pt2, spider); type III DN-cadherin (DN, fruit fly); Sm1-cadherin (Sm1, centipede); type IVb Le1-cadherin (Le1, sea slater); type IVa DE-cadherin (DE, fruit fly); type III Pn-cadherin (Pn, fish); type II Mm5-cadherin (Mm5, mouse). Double-headed horizontal arrows indicate gaps detected between regions of collinear similarity

To systematically detect the possible homologous regions between the diverse classical cadherins in various arthropods and other bilaterians, we searched for collinear arrangements of similarities between their amino acid sequences using blast-based dot-plot comparisons with the amino acid sequences of the Pt1- and Pt2-cadherins as the reference sequences. Using a sliding window of 120 amino acids, we generated a series of overlapping sequences from the entire amino acid sequences of DN-, Sm1-, Le1-, and DE-cadherins, *Pundamilia nyererei* (teleost fish) Pn-cadherin (vertebrate type III), and *Mus musculus* (mammal) Mm5-cadherin (also known as VE-cadherin; vertebrate type II). The serial sequences were then blasted against each reference (i.e., Pt1- and Pt2-cadherin), and the resulting E-values at the blast-hit positions were plotted to visualize the collinearity and identify evolutionarily conserved regions (Fig. 3).

The resulting dot-plots indicated that DN-cadherin is well conserved with the chelicerate type III cadherins throughout its length and that Pn-cadherin has 14 EC domains that correspond to the EC4-EC17 domains of Pt1- and Pt2-cadherins (Fig. 3). However, the three Pn-cadherin (vertebrate type III) EC domains that corresponded to the EC1-EC3 domains of arthropod type III cadherins were too divergent to detect. To avoid confusion, however, the EC domains of all the type III cadherins in the present study were numbered based on the detected collinear arrangements with the numbered EC domains of Pt1- and Pt2-cadherin (Additional file 2: Figure S1). The blast-based dot-plot comparisons revealed correspondence between the EC1-EC6 region of the type IVa cadherins and the EC8-EC13 region of the type III cadherins, as well as between the EC7-LG region of the type IVa cadherins and the EC17-LG1 region of the type III cadherins (Fig. 3). However, although the conservation between the EC9-LG region of Le1-cadherin and the EC17-LG1 region of the type III cadherins was evident, the comparisons of Le1-cadherin against the Pt1- and Pt2-cadherins yielded less clear patterns than were observed for some of the other comparisons (Fig. 3). In addition, the five EC domains of the type II Mm5-cadherin yielded weak but specifically detectable collinear plots with the five C-terminal EC domains of Pt2-cadherin, although the pattern was less clear in the comparison of Mm5- and Pt1-cadherins (Fig. 3).

**Fig. 4:**
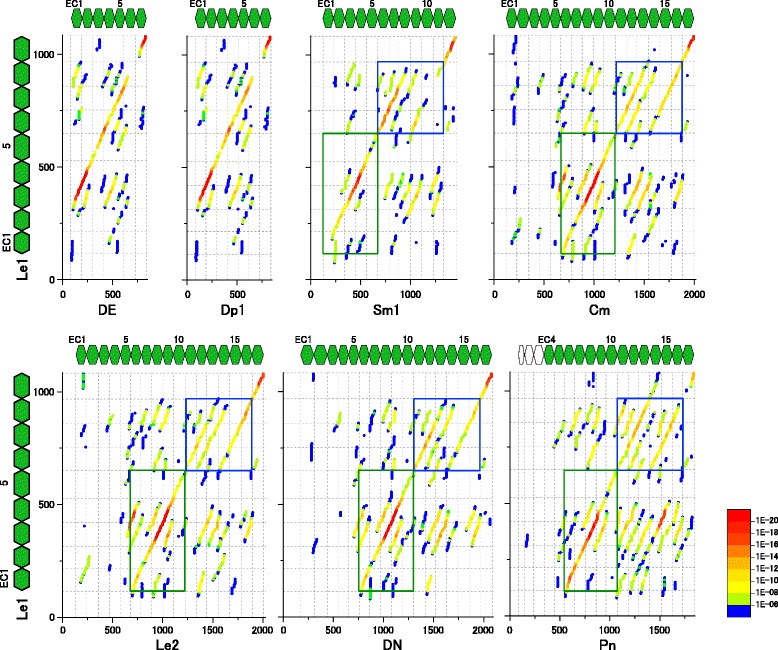
Blast-based dot-plot comparisons between the amino acid sequence of Le1-cadherin and those of other classical cadherins. The classical cadherins analyzed are as follows: type IVb Le1-cadherin (Le1, sea slater); type IVa DE-cadherin (DE, fruit fly); type IVa Dp1-cadherin (Dp1, water flea); Sm1-cadherin (Sm1, centipede); type III Cm-cadherin (Cm, shrimp); type III Le2-cadherin (Le2, sea slater); type III DN-cadherin (DN, fruit fly); type III Pn-cadherin (Pn, fish). Green boxes indicate comparisons between the EC1-EC5 region of Le1-cadherin and the EC6-EC10 regions of the type III cadherins or the corresponding region of Sm1-cadherin, which exhibited marked collinear similarities. Note that the EC1 domain of Sm1-cadherin appears to be divergent. Blue boxes indicate comparisons between the EC6-EC8 region of Le1-cadherin and the EC11-EC16 regions of the type III cadherins or the corresponding region of Sm1-cadherin, which exhibited ambiguous collinear similarities. Note that strong specific signals for collinear similarities were detected between the EC6-EC8 regions of Le1- and Sm1-cadherins

Since the difficulty of aligning the Le1-cadherin sequence might have stemmed from its divergence from the Pt1- and Pt2-cadherin sequences, we also compared the Le1-cadherin sequence to the sequences of the DE-, Dp1-, Sm1-, Cm-, Le2-, DN-, and Pn-cadherins (Fig. 4). These comparisons provided clearer patterns of collinearity and revealed correspondences between the EC3-EC9 region of Le1-cadherin and the EC1-EC7 region of the type IVa cadherins, although the sequence of the Le1-cadherin EC8 domain appeared to diverge from that of the type IVa cadherin EC6 domain. The correspondence between the EC1-EC5 region of Le1-cadherin and the EC6-EC10 region of the type III cadherins and between the EC2-EC5 region of both Le1-cadherin and Sm1-cadherin were also supported (Fig. 4, green boxes), whereas the EC6-EC8 region of Le1-cadherin demonstrated ambiguous affinities to the EC11-EC13, EC12-EC14, and EC14-EC16 regions of the type III cadherins (Fig. 4, blue boxes). Nonetheless, the correspondence between the EC6-EC7 regions of the Le1- and Sm1-cadherins was specifically supported. Similar results were also obtained with the other type IVb cadherins (Additional file 6: Figure S5).

Comparison of the exon-intron organization of type II, III, IVa and IVb cadherins

**Fig. 5:**
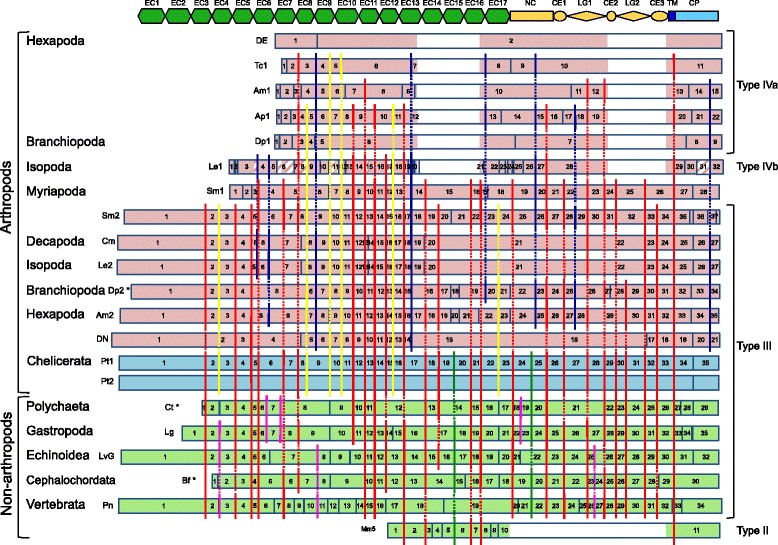
Schematic representation of conserved intron positions among *classical cadherin* genes. Colored rectangles indicate protein-coding regions of the transcripts of the *Pt1-* and *Pt2-cadherin* (light blue), non-spider arthropod *classical cadherin* (pink), and non-arthropod *classical cadherin* (green) genes. The *classical cadherin* genes shown are as follows: *DE-cadherin* (DE, fruit fly); *Tc1-cadherin* (Tc1, beetle); *Am1-cadherin* (Am1, honey bee); *Ap1-cadherin* (Ap1, aphid); *Dp1-cadherin* (Dp1, water flea); *Le1-cadherin* (Le1, sea slater); *Sm1-cadherin* (Sm1, centipede); *Sm2-cadherin* (Sm2, centipede); *Cm-cadherin* (Cm, shrimp); *Le2-cadherin* (Le2, sea slater); *Dp2-cadherin* (Dp2, water flea); *Am2-cadherin* (Am2, honey bee); *DN-cadherin* (DN, fruit fly); *Pt1-cadherin* (Pt1, spider); *Pt2-cadherin* (Pt2, spider); *Ct-cadherin* (Ct, polychaete); *Lg-cadherin* (Lg, snail); *LvG-cadherin* (LvG, sea urchin); *Bf-cadherin* (Bf, amphioxus); *Pn-cadherin* (Pn, fish); and *Mm5-cadherin* (Mm5, mouse). Regions of the transcripts for which genomic sequences were not available are indicated by slanted stripes. A schematic illustration exhibiting the domain structure of Pt1-cadherin is placed at the top as a positional reference. All identified intron insertion sites are shown, and the exons are tentatively numbered from the 5′-terminal side of each transcript. Conserved intron positions that were identified based on the alignment of the amino acid sequences (Additional file 10: Figure S8) are represented by colored vertical lines. The yellow lines indicate arthropod-specific intron positions, whereas the green lines indicate conserved intron positions between the *Pt1-* and *Pt2-cadherin* genes and some of the non-arthropod bilaterian *classical cadherin* genes but not among any other arthropod genes. The red lines indicate intron positions that are conserved both between the *Pt1-* and *Pt2-cadherin* genes and some of the other arthropod genes and between the spider genes and some of the non-arthropod bilaterian genes. The blue lines indicate conserved intron positions between some of the non-spider arthropod *type III cadherin* genes and some of the *type IV cadherin* genes, and the purple lines show the conservation of intron positions within the non-arthropods

To assess the conservation of the exon-intron organization among *classical cadherin* genes, we constructed an alignment of the amino acid sequences of 21 bilaterian classical cadherins, including arthropod and non-arthropod type III cadherins, hexapod and branchiopod type IVa cadherins, a non-branchiopod type IVb cadherin, and a vertebrate type II cadherin (Additional file 9: Figure S7). Large gaps were introduced based on the results shown in Figs. 3 and 4, so that the likely homologous regions of the sequences could be aligned. Although many regions of the cadherins, including their N-terminal regions, remained poorly aligned, we only considered unambiguously aligned regions of the sequences during the analysis (Fig. 5; Additional file 9: Figure S7).

As previously mentioned (Fig. 1), there were 34 introns inserted in the respective coding regions of the *Pt1-* and *Pt2-cadherin* genes, and all of the intron insertions were located at identical sites between the two genes. Notably, 33 of the 34 intron insertion sites were shared among four or more of the non-chelicerate bilaterian genes examined (Fig. 5, red, yellow and green lines), and of those 33 intron insertion sites, only six were specific to arthropods (Fig. 5, yellow lines), and two were shared only with non-arthropod genes (Fig. 5, green lines). Despite the differences in domain organization, six of the 10 intron insertions in the *type II cadherin* gene were conserved in the *Pt1-* and *Pt2-cadherin* genes, as well as in the gastropod and echinoderm *type III cadherin* genes (Fig. 5, red and green lines). Among the arthropod genes, eight intron insertion sites were conserved between the *type III* and *type IV cadherin* genes but were missing in the spider and non-arthropod *classical cadherin* genes (Fig. 5, blue lines). Taken together, these observations indicated that many of the introns in the *Pt1-* and *Pt2-cadherin* genes were inherited in a complex ancestral state rather than acquired via lineage-specific gains of introns and suggested that at least 33 of the 34 introns in the *Pt1-* and *Pt2-cadherin* genes predate the earliest divergence of extant arthropod groups.

Notably, comparisons between the exon-intron organizations of type IVb and arthropod type III cadherins in their EC coding regions revealed marked conservation between them despite their divergence at the amino acid sequence level (Fig. 5; Additional file 7: Figure S6), while the ancestral patterns of intron insertions were less conserved in *type IVa cadherin* genes. The EC10-EC13 coding regions of arthropod *type III cadherin* genes contained eight conserved intron insertions, seven of which were conserved in the EC5-EC8 coding region of the *Le1-cadherin* and other *type IVb cadherin* genes (Fig. 5; Additional file 7: Figure S6). Similarly, the EC1 coding region of the *type IVb cadherin* genes had two intron insertion sites conserved in the EC6 coding region of some of the pancrustacean *type III cadherin* genes (i.e., the *Le2-* and *Cm-cadherin* genes). These observations, together with the results of the blast-based dot-plot comparisons, strongly suggested that the EC1-EC7 region of type IVb cadherins and the adjacent EC8 domain were homologous to the EC6-EC12 region of type III cadherins and the adjacent EC13 domain.

Despite the simple exon-intron organization of the *DE-* and *Dp1-cadherin* genes, some other type IVa cadherins shared a considerable number of intron insertions with *type III cadherin* genes (Fig. 5, red, yellow, and blue lines). This finding indicated that the *type IVa cadherin* genes had experienced varying degrees of intron loss, depending on their specific lineage. Conversely, the *Ea1-cadherin* gene contained many additional introns whose positions were not shared with the *type III* or *type IVb cadherin* genes (Fig. 2; Additional file 4: Figure S3). In this case, we concluded that the additional complexity had resulted from lineage-specific gains of introns.

### Phylogenetic characterization of type IVa and type IVb cadherins

To validate the proposed classification of the type IVa and type IVb cadherin subtypes in the phylogenetic context, we analyzed the amino acid sequences of type IVb cadherins more extensively. The patterns for type IVb cadherins in the blast-based dot-plot comparisons indicated the divergence of their amino acid sequences. Although the EC1-EC2 region of type IVb cadherins was shown to exhibit the highest affinity to the EC6-EC7 region of type III cadherins among the classical cadherins examined, it might be possible that the N-terminal two EC domains in type IVb cadherins have a unique history. To test this possibility, we blasted the amino acid sequences of the EC1-EC2 region, as well as of the EC3-EC4 region, of Le1-, Ha1- and Ph1-cadherin against the Reference Sequence (RefSeq) protein databases for *D. melanogaster* and *Tribolium castaneum* (Additional file 8: Table S4). In all but one of the blast results, the top hit proteins were type III classical cadherins, and the hit sites were consistent with the detected collinear similarities between the type III and type IVb cadherin EC domains (Fig. 4; Additional file 6: Figure S5). These findings strongly suggested that the EC1-EC2 region of the type IVb cadherins shares a relatively recent common history with the EC6-EC7 region of the type III cadherins, and is compatible with the presence of conserved intron insertions in the EC1 coding region of the *type IVb cadherin* genes and the EC6 coding region of the *Le2-* and *Cm-cadherin* genes.

**Fig. 6:**
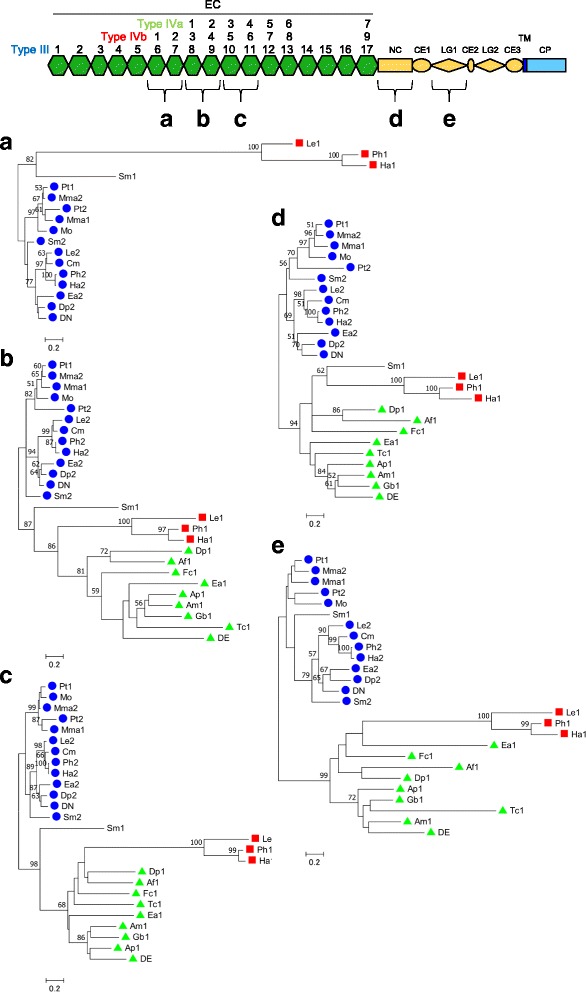
Amino acid substitution-based phylogenetic analyses of the amino acid sequences of five different extracellular regions of the arthropod classical cadherins using the ML method. The five analyzed different extracellular regions of the type III, type IVa and type IVb cadherins (**a-e**) are schematically represented at the top. The type III (*blue*), type IVa (orange), and type IVb (*red*) cadherins are indicated by blue circles, red squares and green triangles, respectively. Numbers at nodes are bootstrap values based on 100 replicates. Nodes with no numbers have support with lower bootstrap values (<50)

Furthermore, we performed phylogenetic analyses of the amino acid sequences of five different extracellular regions (including three EC regions and two non-EC regions) of arthropod type III, type IVa, type IVb and Sm1-cadherins using the maximum likelihood (ML) method (Fig. 6). The results of these analyses revealed the separation of type IVa/IVb cadherins from type III cadherins as well as the separation of type IVb cadherins from type IVa cadherins. The data from all the different regions consistently indicated deep divergence between type IVa and type IVb cadherins, validating the classification of these type IV cadherin subtypes. In addition, the position of Sm1-cadherin was varied among the ML trees, although one of them had support for its association with type IVb cadherin branch.

### Conserved cysteine residues in specific subsets of classical cadherins

**Fig. 7:**
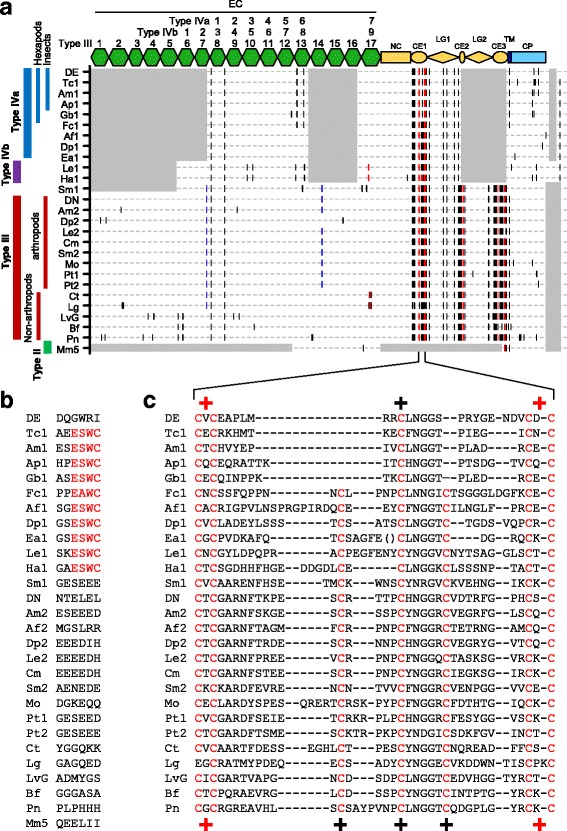
Comparison of the distribution patterns of cysteine residues among classical cadherins. **a**. Comparative diagram of the distribution patterns of cysteine residue in arthropod and non-arthropod bilaterian classical cadherins. In addition to the 21 classical cadherins shown in Fig. 5, the following seven classical cadherins were used: Gb1-cadherin (Gb1, cricket); Fc1-cadherin (Fc1, springtail); Af1-cadherin (Af1, brine shrimp); Af2-cadherin (Af2, brine shrimp); Ea1-cadherin (Ea1, copepod); Ha1-cadherin (Ha1, amphipod); and Mo-cadherin (Mo, mite). The relative positions of cysteine residues in the classical cadherins are indicated by short vertical bars. The black bar denotes a solitary cysteine residue (−C-), the blue bar denotes two successive cysteine residues (−C-C-), and the red bar denotes two cysteine residues spaced with a single non-cysteine residue (−C-X-C-). The shaded regions indicate that there are no sequences for comparison. **b** Representation of the six C-terminal-most amino acid residues of the various classical cadherins. The short sequence motif, E-S/A-W-C, is shown in red. **c** Alignment of the amino acid sequences in parts of the CE1 or CE domains of the various classical cadherins. All cysteine residues are highlighted in red. Parentheses denote the omission of seven non-cysteine residues. The difference between the cysteine patterns of the insect type IVa cadherins and the other cadherins is indicated by “+” characters

The alignments of the amino acid sequences of the selected bilaterian classical cadherins (Additional file 9: Figure S7) allowed us to observe that the majority of cysteine residues are conserved in two or more classical cadherins. To determine the phylogenetic range at which each cysteine residue was conserved, we mapped the relative positions of cysteine residues in the amino acid sequences of 28 arthropod and non-arthropod classical cadherins (Fig. 7a). We found that the CE and LG domains demonstrated highly conserved cysteine patterns, and we also found two highly stable cysteine residues in the EC1 domain of type IVa cadherins and the corresponding EC domains of other metazoan classical cadherins, including a *Trichoplax adhaerens* (placozoan) classical cadherin (Additional file 10: Figure S8).

In addition to the presence of stable cysteine residues, other lineage-restricted features were also observed. For example, we identified a short sequence motif (E-S/A-W-C) at the C-terminus as a shared characteristic of both type IVa and type IVb cadherins (with the exception of DE-cadherin; Fig. 7a, b). We also found that EC6 domains of hexapod type IVa cadherins shared a unique pair of cysteine residues (Fig. 7a; Additional file 10: Figure S8) and that the CE domains of insect type IVa cadherins lacked the two other highly conserved cysteine residues (Fig. 7a, c). We also identified six cysteine residues that were specific to the EC5, EC8 and EC9 domains of type IVb cadherins and two cysteine residues that were specific to the EC8 domain of hexapod type IVa cadherins (Fig. 7a; Additional file 4: Figure S3; Additional file 10: Figure S8). The lineage-specific cysteine residues of the type III cadherins included two consecutive cysteine residues in the EC7 domain of arthropods and annelids/molluscans (Fig. 7a), as well as two other consecutive cysteine residues in the EC14 domain, which was limited to arthropods (Fig. 7a). We noted that two cysteine residues were also conserved in the Ca^2+^-binding motifs between the EC5 and EC6 domains in the echinoderm and chordate type III cadherins (Fig. 7a; Additional file 10: Figure S8). However, these cysteine residues were also found in a predicted classical cadherin of the non-bilaterian metazoan *T. adhaerens*. These observations indicated that the patterns of cysteine residues among the classical cadherins of metazoans were stable but varied, thus presumably reflecting rare evolutionary changes.

## Discussion

### Type III cadherin is the ancestral classical cadherin in arthropods as well as in bilaterians

The present study investigated whether type IV or related cadherins were present in all the major arthropod lineages. However, our exhaustive search of multiple chelicerate genomes failed to identify any classical cadherins other than the type III form, which was found in all the arthropods examined. This result corroborates the previous finding that the only *classical cadherin* gene in the genome of the echinoderm *Strongylocentrotus purpuratus* encodes a type III cadherin [16]. It is, therefore, reasonable to assume that, among the various forms of classical cadherin, the type III form is the only one known to have been passed on from the last common ancestor to both the arthropod and echinoderm lineages and that the same form is also the only one known to have been passed on from the earliest arthropods to all the major extant arthropod groups.

Importantly, these hypotheses provide an explanation for the relationships between the various states of classical cadherin domain organization and exon-intron organization observed both within and beyond the Arthropoda. The findings of the present study indicate that the markedly complex exon-intron organization of the *P. tepidariorum type III cadherin* genes is representative of the ancestral state for arthropods (Figs. 1 and 5), and the detection of numerous conserved intron positions between the spider and non-arthropod bilaterian *classical cadherin* genes also indicated that the complex exon-intron organization predates the divergence of the arthropod lineage from other bilaterians. Our finding that some of the *type IV cadherin* genes have retained ancestral states of exon-intron organization provides genomic evidence for the derivation of type IV cadherin from type III cadherin.

Similarly, the *5-EC cadherin* genes, which are prevalent in vertebrates (i.e., *type I* and *type II cadherin* and *non-classical desmosomal cadherin* genes), also possess a conserved exon-intron organization [45, 46], indicating that they share a common precursor in the lineage that gave rise to vertebrates. The identification of six shared intron positions among arthropod *type III* and vertebrate *type II cadherin* genes (Fig. 5) also indicated a deep link among the different classical cadherins, paving the way toward a comprehensive framework for the divergence of *classical cadherin* genes among bilaterians.

### Evolution and divergence of type IV cadherins

An unexpected and important finding of this work was the identification of a novel form of classical cadherin in isopod and amphipod crustaceans that was similar to, but distinct from, the known hexapod and branchiopod type IV cadherins. This finding led us to propose a revision of the type IV cadherin category and to define two subclasses, type IVa and type IVb. This classification was validated based on comparative and phylogenetic analyses of the arthropod classical cadherins.

**Fig. 8:**
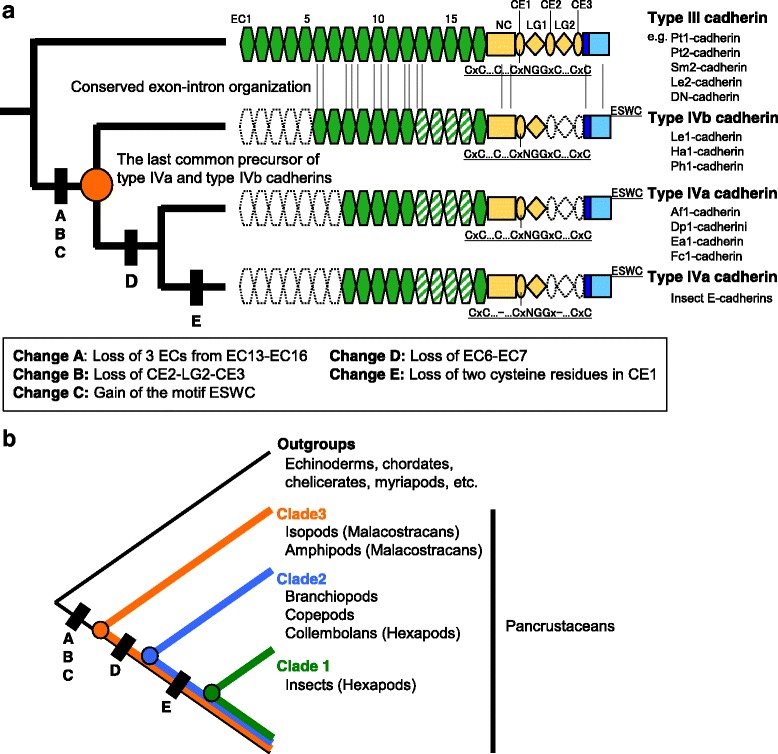
Reconstruction of the evolution of the various forms of classical cadherin in arthropods. **a**. Schematic representation of a proposed stepwise reduction model that explains the derivation of insect type IV cadherin from the ancestral type III cadherin. Possible homologous regions between different cadherins are placed at the same positions. To avoid confusion, the domains of the derived cadherins are specified according to the type III cadherin organization (*top*). Changes A-C preceded the last common precursor of type IVa and IVb cadherins (*orange* circle). The four EC domains that were putatively reduced to a single EC domain (by Change A) are indicated by slanted stripes. Conserved exon-intron insertion sites identified between the *type IVb cadherin* genes and some *type III cadherin* genes are indicated by vertical lines (see Fig. 5). The two extra EC domains of type IVb cadherin, which are missing in the type IVa cadherin, were characterized as part of a stretch of EC domains that had been inherited from the ancestral type III cadherin. This indicates that the last common type IVa/IVb cadherin precursor possessed, in addition to the seven EC domains, two EC domains corresponding to the type III cadherin EC6 and EC7 domains. The type IVa cadherin is likely to have arisen through the loss of these two EC domains (Change D), followed by Change E in the insect lineage. **b**. Schematic cladogram of the proposed phylogenetic relationships among pancrustacean subgroups. Changes A-E define three clades, Clades 1–3

The identification and characterization of type IVb cadherin provided an opportunity to discuss the transition from the ancestral type III cadherin to the insect type IVa cadherin (Fig. 8a), which is often referred to as E-cadherin. Comparative data presented in this and previous research [15] suggest that the origination of the last common precursor of type IVa and IVb cadherins was associated with a duplication of a preexisting *type III cadherin* gene followed by, or coupled with, the following three changes: the loss of 3 EC domains from the region corresponding to the EC13-EC16 region of type III cadherin (Fig. 8a, Change A), the loss of the region corresponding to the CE2-LG2-CE3 region of type III cadherin (Fig. 8a, Change B), and the gain of the C-terminal motif E-S-W-C (Fig. 8a, Change C).

Our dot-plot and genomic data indicated that both type IVa and type IVb cadherins have tandem EC domains that were derived from the EC8-EC12 region of type III cadherin, and our phylogenetic analyses based on amino acid alignment and substitution supported deep divergence between type IVa and type IVb cadherins. The key issue is what form the last common precursor of type IVa and type IVb cadherins had. There are two lines of evidence for the primitiveness of the type IVb subtype. First, the amino acid sequences of the two extra EC domains (the EC1-EC2 regions) of type IVb cadherins exhibit specific affinities to those of the EC6-EC7 regions of type III cadherins (Fig. 4; Additional file 8: Table S4). Second, the *type IVb cadherin* genes have retained many intron insertions that are shared with the arthropod *type III cadherin* genes (Figs. 5, 8a). Notably, the conservation of two intron insertion sites in the EC1 coding region of the *type IVb cadherin* genes and the EC6 coding region of the *type III Cm-* and *Le2-cadherin* genes suggest a specific association between the type IVb and type III forms. Altogether, the occurrence of these primitive characters in the *type IVb cadherin* genes is most easily understood if we consider that the two N-terminal-most EC domains and subsequent (at least) five EC domains of the type IVb cadherin are composed of a continuous stretch of EC domains that were inherited from the ancestral type III cadherin (Fig. 8a). This interpretation does not necessarily indicate that the last common precursor of type IVa and type IVb cadherins was a type IVb cadherin but does indicate that it could have at least nine EC domains. If this is the case, the transition from the last common precursor of type IVa and type IVb cadherins to type IVa cadherin should have occurred through the loss of two EC domains corresponding to the EC6-EC7 region of type III cadherin (Fig. 8a, Change D). Duplication of the precursor gene and subsequent differential loss of paralogs might have potentially occurred during the processes that gave rise to the type IVa and type IVb cadherin subtypes in the different crustacean lineages. Despite the primitive characteristics of the type IVb subtype, it could also be hypothesized that type IVb cadherin evolved from type IVa cadherin. Indeed, in the vertebrate lineage, 7-EC non-classical cadherins (e.g., LI-cadherin) are reported to have arisen from a 5-EC cadherin by internal domain duplication [47]. However, our comparative and phylogenetic analyses of type IVa and type IVb cadherins yielded no sign to support such a scenario. As another possibility, gene conversion might have led to a lineage-specific addition of two EC domains to the type IVa cadherin form. However, considering the determined characteristics of the EC1-EC2 coding regions of the *type IVb cadherin* genes, such a genetic event is much less likely to have occurred than the proposed domain loss event (Fig. 8a, Change D).

The predicted Sm1-cadherin could be categorized as neither a type III nor type IV classical cadherin. Because this cadherin, similar to type IVb cadherins, lacks the five EC domains that correspond to the EC1-EC5 region of type III cadherin, we considered whether Sm1-cadherin and the type IVb cadherins share a common precursor. However, we were unable to identify any specific signature that supported such a hypothesis. To fill the possible gaps in reconstruction of the transition processes between type III and type IVa/IVb cadherins, more data from the myriapod group, as well as from the crustacean group, will be required.

The evolutionary conservation of cysteine residues has been previously reported among type I cadherins or among protocadherins, and conserved cysteine residues are apparently involved in intra- or intermolecular disulfide bonds that contribute to the maintenance or stabilization of functional structures [48, 49]. In the present study, our comparative analysis revealed phylogenetically stable, yet varied, patterns of cysteine residues among the metazoan classical cadherins, which might imply transitions of structural mechanisms during classical cadherin evolution. We found that the CE domains of all insect type IVa cadherins specifically lacked two highly conserved cysteine residues. The relationship of the insect type IVa cadherins with other classical cadherins clearly indicated that the simpler pattern of cysteine residues in the CE domains of the insect type IVa cadherins is a derived state that was produced by the evolutionary loss of two cysteine residues (Fig. 8a, Change E). Therefore, we suggest that the transition from the ancestral type III cadherin to the derived insect type IVa cadherin was a multistep process that involved several progressive reductive changes.

### Directionality in the evolution of the classical cadherin structure

Accumulated evidence from the arthropods and chordates suggests that the lineage-specific forms of classical cadherin have been shaped by reductive changes from an ancestral type III cadherin. The genomes of certain non-bilaterian metazoans are known to contain genes encoding much larger classical cadherins than type III cadherin [2, 17]. Therefore, it is likely that reductive changes also preceded the establishment of the type III cadherin [2].

In fact, the modification of cadherins by reductive changes from a larger state seems to be a common trend in the evolution of the classical cadherin structure. This directionality could possibly be associated with the cost and efficiency of the mechanical energetic processes by which classical cadherin-based adherens junctions drive morphogenesis. However, it is difficult to imagine that large deletions would instantly enhance the performance of classical cadherin molecules. Nonetheless, if such a deletion mutation did not disrupt the homophilic cell-cell binding properties of the classical cadherin but did alter its binding specificity or strategy, the affected cadherin might have had a chance to evolve independently of the parental cadherin.

Investigating the mechanism by which the classical cadherins were able to evolve via simplification is a typical challenge in experimentation-based evolutionary biology. The multistep transition from the ancestral type III cadherin to the insect type IVa cadherin offers an example with which to investigate the evolvability of type III cadherin. Genetic analysis of a DE-cadherin in which the EC7 and subsequent membrane-proximal extracellular domains had been deleted suggested that the six N-terminal-most EC domains constitute a functional unit that is capable of mediating cell-cell adhesion, whereas the membrane-proximal domains are necessary to ensure the functionality of the DE-cadherin during morphogenesis [26].

Recently, atomic force microscopy imaging revealed that the EC1-EC4 region of DE-cadherin forms a tightly folded, globular structure [27], and the deeply bent conformation of the EC region is likely to be associated with the Ca^2+^-free linker found between the EC2 and EC3 domains of type IVa cadherin, which is conserved in the corresponding EC domains (EC9 and EC10) of type III cadherin [36]. The EC2-EC4 region of hexapod type IVa cadherins has been demonstrated as the minimal portion capable of mediating exclusive homophilic binding specificity. Intriguingly, three consecutive EC domains of the type III DN-cadherin that correspond to the EC2-EC4 region of type IVa cadherins are able to specifically recognize the DN-cadherin as the binding partner. A series of these findings implies that type IVa cadherin might have inherited part of an as-yet-uncharacterized mechanism of homophilic binding from the type III cadherin. In this context, questions of how the two N-terminal-most ECs of type IVb cadherin contribute to the functioning of the type IVb cadherin and how the large number of EC domains in type III cadherin are utilized to mediate homophilic cell-cell adhesion are key to a better understanding of the stepwise reductive changes involved in the evolution of insect E-cadherin.

### Implications for pancrustacean phylogeny

Phylogenetic inferences of deep relationships are often problematic [50–53]. For example, the reconstruction methods used in the majority of modern phylogenetic analyses are based on nucleotide or amino acid substitutions in orthologous genes or proteins; however, the deep branch topologies of the resulting phylogenetic trees can be influenced by the sampling of species, choice of sequence evolution models, and variations in substitution rates among species, among sites, and even at individual sites over time [51]. Therefore, any phylogenetic hypothesis based on such analyses should be verified using alternative methods, one of which is the identification of rare genomic changes [54, 55].

In the present study, we identified stable yet varied character states among the arthropod *classical cadherin* genes, which provided clues to the polarity of cadherin evolution. A thematically similar situation has been described among the phylum Chordata [32, 33]. For example, the 5-EC state of classical cadherin (type I/II cadherin) defines a clade that includes the vertebrates and urochordates, but excludes the cephalochordates, a relationship that is supported by various independent phylogenetic studies [56–58]. In the phylum Arthropoda, the paraphyly of crustaceans has been firmly established, based on analyses of nucleotide and amino acid substitutions and rare genomic changes, and the crustaceans are now suggested to form a clade with hexapods, termed Pancrustacea, that excludes the myriapods and chelicerates [59–63]. The monophyly of both hexapods and insects has also been strongly supported by recent molecular phylogenies [64–70]. However, the relationships between the hexapods and other pancrustacean subgroups remain a controversial topic [71, 72].

One of the major conflicts regarding the pancrustacean phylogeny concerns the relationships between hexapods, branchiopods, and malacostracans. Neural cladistics and some molecular studies propose a closer relationship between the hexapods and malacostracans than between the hexapods and branchiopods [73–77], whereas various recent studies that have used well-sampled, large-scale sequence data support a closer relationship between the hexapods and branchiopods [64, 67, 68, 78–80], and some phylogenies even suggest that the branchiopods and malacostracans are more closely related to each other than to the hexapods [65].

The complementary distributions of polarized genomic characters related to type IVa/IVb cadherins could be informative for the hexapod-branchiopod-malacostracan relationship. Based on these characters, we propose three successively nested clades, Clades 1–3 (Fig. 8b), in which Clade 1 includes insects but excludes collembolans, branchiopods, copepods, malacostracans, and non-pancrustacean arthropods, Clade 2 includes Clade 1, collembolans, branchiopods, and copepods but excludes malacostracans (such as isopods and amphipods) and non-pancrustacean arthropods, and Clade 3 includes Clade 2 and malacostracans but excludes non-pancrustacean myriapods and chelicerates. This topology indicates that branchiopods have a closer relationship to hexapods than malacostracans.

The matter of which crustacean subgroup is the closest relative of hexapods is also a subject of debate. Branchiopods [64, 66, 67, 81, 82], remipedians [61, 68, 69] and a clade that includes both remipedians and cephalocarids [65] have all been proposed as candidates for the sister group of hexapods. A few studies of ribosomal RNA sequences have also proposed copepods as the sister group of hexapods [78, 83]; however, recent molecular studies have frequently placed the copepods within a clade with the malacostracans [64, 65, 67, 68, 79, 81]. Nevertheless, although the present study did not include several key crustacean subgroups, such as Remipedia and Cephalocarida, our phylogenetic hypothesis could indicate that copepods, as well as branchiopods, should be included in the group of potential candidates for the closest relative of hexapods.

Finally, despite our exhaustive searches of the genome sequences for *classical cadherin* genes in the species, it was difficult to entirely exclude the possibility of additional *classical cadherin* genes due to incompleteness of the genome sequence assemblies. It is potentially possible that the establishment of the type IVa and type IVb cadherin subtypes might have preceded the diversification of the crustacean lineages. For example, if the malacostracan genomes are found to have *type IVa cadherin* genes in addition to the *type IVb cadherin* genes, our phylogenetic proposals should be reconsidered or rejected. Furthermore, because the number and range of the species examined in the present study were limited, our phylogenetic proposals remain highly hypothetical. We believe that the growing availability of arthropod genome sequences [37] will soon facilitate a more comprehensive analysis of type IV cadherin-related character states and help evaluate the conflicting hypotheses for arthropod phylogeny.

## Conclusions

In the present study, we provided a genomic perspective of the evolution of classical cadherins among bilaterians, with a focus on the phylum Arthropoda. We demonstrated that the *type III cadherin* genes in the chelicerate *P. tepidariorum* were representative of the ancestral genomic organization of classical cadherins in arthropods, and suggested that the precursor of insect E-cadherin originated through stepwise reductive changes after the earliest divergence of extant arthropod groups. Future studies should investigate the structural mechanisms underlying the multistep transition from the arthropod ancestral type III cadherin to the more recent insect E-cadherin. The varied, polarized, and stable character states of classical cadherins could be widely applicable as indicators of deep phylogenetic relationships, as exemplified in the arthropod and chordate phyla.

## Methods

### Animals

This work was performed according to the institutional animal care and use committee guidelines (JT Biohistory Research Hall). Laboratory stocks of *Parasteatoda tepidariorum* (formerly *Achaearanea tepidariorum*) were derived from individuals collected at several different sites in Kyoto and Osaka, Japan [84]. Adults of *Ligia exotica* were collected from Kobe, Hyogo, Japan; and adults of *Caridina multidentata* (formerly *Caridina japonica*) were purchased from local suppliers.

### Sequencing

For transcriptome sequencing with the Roche GS FLX+ system, total RNA was isolated from *P. tepidariorum* embryos at stages 1, 3, 5, 7, and 9, using the MagExtractor RNA nucleic acid purification kit (Toyobo). Poly (A) + RNA was purified from the total RNA and used to generate a cDNA library (GATC Biotech). First-strand cDNA synthesis was primed with a N6 randomized primer, and adaptors were ligated to the 5′ and 3′ ends of the cDNA, followed by 17 cycles of PCR amplification. The amplified cDNA was normalized using a single cycle of denaturation and re-association, and the re-associated ds-cDNA was separated from the remaining ss-cDNA using a hydroxylapatite column. Subsequently, the ss-cDNA was PCR amplified (7 cycles), and 500–850 bp-long cDNA fragments were eluted from an agarose gel and then sequenced, yielding 842,126 reads with an average length of 426 bp. These raw reads were subjected to adaptor trimming and de novo assembly using the CLC Genomics Workbench Version 7.0.3 (Qiagen) with the following settings: Bubble size, Automatic = 425; Word size, Automatic = 21; Map reads back to contigs, Yes (Mismatch cost, 2; Insertion cost, 3; Deletion cost, 3; Length fraction 0.9; Similarity fraction, 0.9); Update contigs, Yes. Some misassembled contigs were manually corrected. The resultant transcriptome assembly consisted of 23,144 contigs with N50 of 1046 bp (SRA Accession: DRR054577; Sequence Accession: IABY01000000).

For RNA-seq with the Illumina MiSeq system, mRNA was purified from stage-5 and stage-10 *P. tepidariorum* embryos, late stage *L. exotica* embryos, and adult *C. multidentata* muscle and neural tissues, using the QuickPrep Micro mRNA Purification Kit (GE Healthcare). The mRNAs were fragmented using the NEBNext RNase III RNA Fragmentation Module (New England BioLabs) and then used to construct DNA libraries with the NEBNext Ultra Directional RNA Library Prep Kit for Illumina (New England BioLabs) and NEBNext Multiplex Oligos for Illumina (Index Primers Set 1, New England BioLabs). Sequencing reactions were performed using the 150- or 500-cycle formats of the Illumina MiSeq Reagent Kit, and the resulting raw sequence reads were subjected to adaptor trimming and de novo assembly (Additional file 1: Table S1) using the CLC Genomics Workbench with the following settings: Bubble size, Automatic = 50; Word size, Automatic = 24; Perform Scaffolding, Yes; Auto-detect paired distance, Yes; Map reads back to contigs, Yes (Mismatch cost, 2; Insertion cost, 3; Deletion cost, 3; Length fraction 0.9; Similarity fraction, 0.9); Update contigs, Yes. For gene expression analysis, the adaptor-trimmed reads were mapped to the sequences of selected transcripts, using the CLC Genomics Workbench (Mismatch cost, 2; Insertion cost, 3; Deletion cost, 3; Length fraction 0.9; Similarity fraction, 0.9), and counted in order to quantify the expression levels were quantified as reads per kilobase of exon per million total reads.

For genome sequencing with the Illumina MiSeq system, genomic DNA was isolated from late stage *P. tepidariorum* embryos, late stage *L. exotica* embryos, and adult *C. multidentata* muscle and neural tissues, using the GenomicPrep Cells and Tissue DNA Isolation Kit (GE Healthcare). The isolated DNA was fragmented using an acoustic solubilizer (Covaris), and the fragmented DNA (250–400 bp for *L. exotica* and *C. multidentata* DNA and 250–400, 400–500, 500–600, or 600–800 bp for *P. tepidariorum* DNA) was used to construct DNA libraries with the Truseq DNA Sample Prep Kit (Illumina). Paired-end sequencing of the libraries was performed using the 500- or 600-cycle formats of the Illumina MiSeq Reagent Kit, and the resulting raw sequence reads were subjected to adaptor trimming and de novo assembly (Additional file 1: Table S3) using the CLC Genomics Workbench with the following settings: Bubble size, Automatic = 227 (for *L. exotica*) or 219 (for *C. multidentata*); Word size, 64; Perform Scaffolding, Yes; Auto-detect paired distance; Yes. The approximate coverage depth for the obtained genomic sequences was estimated by mapping the reads to the 1307-, 1226- and 2142-bp regions of the *P. tepidariorum* genome (corresponding to exon 2 of the *Pt1-cadherin* gene and exons 1 and 35 of the *Pt2-cadherin* gene in Fig. 1), the 1010-, 2281-, and 914-bp regions of the *L. exotica* genome (corresponding to exon 28 of the *Le1-cadherin* gene and exons 21 and 22 of the *Le2-cadherin* gene in Fig. 5), or the 1392-, 2275-, and 914-bp regions of the *C. multidentata* genome (corresponding to exons 1, 21, and 22 of the *Cm-cadherin* gene in Fig. 5).

### Identification of *classical cadherin* genes

Partial *Pt2-cadherin* sequences were originally found in the *P. tepidariorum* transcriptome that was generated using the Roche GS FLX+ system, and the full-length *Pt2-cadherin* cDNA was isolated from cDNA libraries of *P. tepidariorum* embryos [84] and then sequenced. The *Le1-cadherin* transcript was predicted from the de novo assembly of the *L. exotica* RNA-seq reads. A cDNA fragment that corresponded to the coding region of the *Le1-cadherin* transcript was amplified from oligo-dT primed cDNA of late stage *L. exotica* embryos by PCR using the following primers: 5’-ATAAGAATGCGGCCGCATCGGTGAACAAATCTTCAGGTTCA-3’; 5’-ATAAGAATGCGGCCGCTTAGCACCAAGATTCCTTGCTCTG-3′ (Underlines indicate the *NotI* recognition sites). This product was digested with *NotI* and then cloned into the *NotI* site of pUAST, resulting in pUAST-Le1-cadherin. This cloned cDNA was sequenced to validate the predicted transcript.

We searched for *classical cadherin* sequences in arthropod genomes available from public databases, as well as in genomic and transcriptomic sequences generated from *P. tepidariorum*, *L. exotica* and *C. multidentata* in this study (Table 1; Additional file 1: Tables S1 and S3). For the initial detection of classical cadherin-encoding gene (s), the amino acid sequences of the entire CP domains of Pt1-, Pt2-, DE-, DN-, Le1-, Le2-, Gb1-, Af1-, and Dp1-cadherins were blasted against each genome sequence assembly or the WGS and RNA-seq reads and assemblies (*P. tepidariorum*, *L. exotica* and *C. multidentata*) using the tblastn algorithm with the cutoff E-value of 1 × 10^−4^. We also blasted the entire amino acid sequences of the Pt1-, DN-, and Le2-cadherins against the identified scaffolds/contigs to determine whether the detected scaffolds contained all the typical type III cadherin elements (14–17 ECs, NC, CE1, LG1, CE2, LG2, CE3, TM, and CP domains) in the expected order. To exhaustively search for *type IV cadherin* genes, the entire amino acid sequences of DE-, Gb1-, Af1-, Dp1- and Le1-cadherins were blasted against the scaffolds/contigs in which classical cadherin CP domain-related sequences were found.

In cases where only some of the type III or type IV cadherin elements were found in the scaffolds/contigs, we examined the possibility that the remaining elements might be encoded in other scaffolds/contigs. To identify neighboring exons of a gene within the same or different scaffolds/contigs, we also used predicted transcript sequences, RNA-seq reads and transcriptome assemblies that were either publicly available or generated in the present study (Table 1; Additional file 1: Tables S1 and S3; Additional file 11: Table S5). The transcript sequences for the hypothetical Mma1-, Mma2-, Sm1-, Sm2-, Ea1-, and Ea2-cadherins were reconstructed from publicly available RNA-seq reads of *Strigamia maritima* (PRJNA246089), and *Eurytemora affinis* (PRJNA275666) using the CLC Genomics Workbench or Geneious Version 9.0.5 (Biomatters). RNA-seq reads of *Hyalella azteca* (PRJNA277380) [85] were also used to assess the transcript models for Ha1- and Ha2-cadherin. To identify type IV cadherin-related sequences in the *C. multidentata* genome, portions of the amino acid sequence of Le1-cadherin were blasted against the WGS reads.

### Sequence analysis and characterization

The sources of genomic, mRNA and protein sequences of classical cadherins used for sequence alignment, dot-plot analysis, exon-intron structure analysis and phylogenetic analysis are listed in Additional file 11: Table S5. The amino acid sequences of classical cadherins were aligned using the ClustalW algorithm with the following settings: Cost matrix, BLOSUM; Gap open cost, 10; Gap extend cost, 0.1. With the exception of the alignments shown in Additional file 3: Figure S2 and Additional file 7: Figure S6, the alignments were followed by manual adjustment, considering the results of the dot-plot analyses (Figs. 3 and 4), as well as other conserved motifs or residues. Exon-intron boundaries of the genes were determined by comparing the transcript and genome assembly sequences, considering the GT-AG mRNA processing rule. Importantly, to determine the exon-intron boundaries of the *L. exotica* and *C. multidentata* genes, the WGS reads were used.

For the dot-plot analysis, a series of overlapping 120-residue amino acid sequences were generated from the amino acid sequences of each cadherin using a sliding window with a step of five amino acids. The individual sequences were blasted against the reference sequences, using the blastp algorithm, and the E-values for the individual hits were plotted using color codes. To map the relative positions of cysteine residues in a given cadherin, the sequence alignment shown in Additional file 9: Figure S7 was used as the positional reference.

For amino acid substitution-based analysis, the amino acid sequences from the multiple regions of the indicated cadherins were individually aligned using the ClustalW algorithm with the same settings as above, and ML analyses of the resultant sequence alignments (without manual adjustment) were performed using *MEGA* version 7.0.25 [86]. Model testing was conducted under the Bayesian Information Criterion, which selected the LG + G model as the best-fit model. ML trees were constructed with the following settings: Substitution model, LG + G; Number of discrete gamma categories, 5; Gaps data treatment, complete deletion; ML heuristic method, Subtree-pruning-regrafting (extensive); Initial tree for ML, NJ/BioNJ.

### Cell aggregation assay

The culture of S2 cells, transfection, and cell aggregation assays were conducted as described [25], with some modifications. Briefly, 5 × 10^6^ S2 cells were co-transfected with pUAST-Le1-cadherin, pUAST-mKate2, and pWA-GAL4 (a gift from Yasushi Hiromi, National Institute of Genetics, Japan) at a ratio of 5:5:1, and cells were co-transfected with empty pUAST, pUAST-mKate2, and pWA-GAL4 as a negative control. After 45 h of incubation, the transfected cells were collected and resuspended in 5 ml of culture medium, and then 0.5 ml aliquots of each cell suspension were transferred to individual wells of a 24-well plate and rotated at 150 rpm for 15 min. The cell aggregates formed in the wells were observed and photographed using an Olympus IX71 fluorescence microscope equipped with a UPlanFl 10×/N.A. 0.3 objective, differential interference contrast optics, and a CoolSNAP HQ camera (Roper Scientific).

## Additional files

Additional file 1: Table S1. Statistics and accessions of RNA-seq data from *P. tepidariorum*, *L.exotica* and *C. multidentata*. **Table S2.** Expression levels of selected transcripts from *P. tepidariorum* embryos, as indicated by RNA-seq. **Table S3.** Statistics and accessions of WGS data from *P. tepidariorum*, *L.exotica* and *C. multidentata*. (PDF 91 kb)

Additional file 2: Figure S1. Characterization and subdivision of the amino acid sequences of DN-, Pt1-, and Pt2-cadherins. **A**. Alignment of the EC1-EC17 regions of DN-, Pt1-, and Pt2-cadherins (abbreviated as DN, P1 and P2, respectively). The “-“character indicates introduced gaps. Conserved hydrophobic residues (blue), Ca^2+^-binding motifs or residues (red), and XPXF motif sequences (green) are aligned, all of which represent structural features of EC domains as shown schematically at the top. Thick blue arrows denote the seven β-strands (βA to βG). Each red arrow indicates the inter-EC linker to which the Ca^2+^-binding motif or residue belongs. No residues are omitted from the alignment, except for three sections where 7–12 residues of the DN-cadherin sequences are placed outside the alignment (parentheses). The N-terminal sequence (Nt) preceding the EC1 domain is also shown for each cadherin. **B**. Alignment of the NC and subsequent domains of the DN-, Pt1-, and Pt2-cadherins. In both A and B, conserved cysteine residues are highlighted in pink, and the residues bordering the start and end of the introns are highlighted with yellow and green. (PDF 362 kb)

Additional file 3: Figure S2. Alignment of the entire amino acid sequences of thirteen type III cadherins in arthropods, and comparison of the exon-intron organizations. The alignment was produced using the ClustalW algorithm without manual adjustment. The classical cadherins shown are Pt1-, Pt2-, Mma1-, Mma2-, Mo-, Sm2-, Cm-, Le2-, Ph2-, Ha2-, Ea2-, Dp2-, and DN-cadherins. The domain organization is indicated above the Pt1-cadherin sequence. Blue lines with breakages indicate exons, and the breaking points indicate intron insertion sites revealed by comparisons with the corresponding genomic sequences. (PDF 5991 kb)

Additional file 4: Figure S3. Characterization of the amino acid sequences of type IVa and type IVb cadherins. **A**. Alignment of the amino acid sequences of all EC domains (EC1-EC7 or EC1-EC9) of the DE-, Dp1-, Ea1-, Le1-, Ha1-, and Ph1-cadherins (abbreviated as DE, D1, E1, L1, H1, and Ph1, respectively). Conserved hydrophobic residues (blue), Ca^2+^-binding motifs or residues (red), and XPXF motif sequences (green) are aligned. Thick blue arrows denote the seven β-strands (βA to βG), and each red arrow indicates the inter-EC linker to which the Ca^2+^-binding motif or residue belongs. No residues are omitted from the alignment, except for three instances where 5–7 residues from the Le1- or Ha1-cadherin sequences are placed outside the alignment (parentheses). The N-terminal sequence (Nt) preceding the EC1 domain is also shown for each cadherin. **B**. Alignment of the amino acid sequences of the NC and subsequent domains of the DE-, Dp1-, Ea1-, Le1-, Ha1-, and Ph1-cadherins. In both A and B, the conserved cysteine residues are highlighted in pink, and the residues bordering the start and end of the introns are highlighted with yellow and green. (PDF 379 kb)

Additional file 5: **Figure S4.** Schematic representation of detected sequences of *C. multidentata* related to classical cadherins. **A**. Nine reconstructed genomic sequences of *C. multidentata* that contain coding sequences closely related to those of Le1-cadherin. The sequences are available under the indicated accession numbers. **B**. Eight transcriptome contigs connected by raw reads. The sequences of these contigs are available under the indicated accession numbers. Contig33642 was modified by an insertion of 5 nucleotide bases (CCGGA) between the nucleotides 349 and 350 based on assessment of raw reads (asterisk). The assembled transcript and protein sequences are available in Additional file 12. Detected domain elements are shown. (PDF 108 kb)

Additional file 6: Figure S5. Blast-based dot-plot comparisons between the amino acid sequence of Ha1- (A) or Ph1- (B) cadherin and those of DE-, Dp1-, Sm1-, Cm-, Le2-, DN- and Pn-cadherins. Green boxes indicate comparisons between the EC1-EC5 region of Ha1- or Ph1- cadherin and the EC6-EC10 regions of the type III cadherins or the corresponding region of Sm1-cadherin, which exhibited marked collinear similarities. Blue boxes indicate comparisons between the EC6-EC8 region of Ha1- or Ph1-cadherin and the EC11-EC16 regions of the type III cadherins or the corresponding region of Sm1-cadherin, which exhibited ambiguous collinear similarities. (PDF 2623 kb)

Additional file 7: Figure S6. Comparison of the exon-intron organizations of *type IVa*, *type IVb* and *type III cadherin* genes. Alignment of the amino acid sequences of the EC1-EC6 region of type IVa cadherins, the EC1-EC8 region of type IVb cadherins, and the EC6-EC13 region of type III cadherins was produced using the ClustalW algorithm without manual adjustment. The classical cadherins shown are DE-, Dp1-, Ea1-, Le1-, Ha1-, Ph1-, Pt1-, Sm2-, Cm-, Le2-, and DN-cadherins. The EC domains for type IVa, type IVb, and type III cadherin are indicated above the DE-, Le1- and, Pt1-cadherin sequence, respectively. Blue lines with breakages indicate exons, and the breaking points indicate intron insertion sites revealed by comparisons with the corresponding genomic sequences. (PDF 1348 kb)

Additional file 8: Figure S6. Results of blast searches of type IVb cadherin EC domain sequences against the *D. melanogaster*, *T. castaneum* and *D. pulex* RefSeq protein sequences. (XLSX 11 kb)

Additional file 9: Figure S7. Amino acid alignment of selected classical cadherins from arthropods and non-arthropod bilaterians. The classical cadherins shown are as follows: DE-cadherin (DE, fruit fly); Tc1-cadherin (Tc1, beetle); Am1-cadherin (Am1, honey bee); Ap1-cadherin (Ap1, aphid); Gb1-cadherin (Gb1, cricket); Fc1-cadherin (Fc1, springtail); Af1-cadherin (Af1, brine shrimp); Dp1-cadherin (Dp1, water flea); Ea1-cadherin (Ea1, copepod); Le1-cadherin (Le1, sea slater); Ha1-cadherin (Ha1, amphipod); Sm1-cadherin (Sm1, centipede); DN-cadherin (DN, fruit fly); Am2-cadherin (Am2, honey bee); Dp2-cadherin (Dp2, water flea); Le2-cadherin (Le2, sea slater); Cm-cadherin (Cm, shrimp); Sm2-cadherin (Sm2, centipede); Mo-cadherin (Mo, mite); Pt1-cadherin (Pt1, spider); Pt2-cadherin (Pt2, spider); Ct-cadherin (Ct, polychaete); Lg-cadherin (Lg, snail); LvG-cadherin (LvG, sea urchin); Bf-cadherin (Bf, amphioxus); Pn-cadherin (Pn, fish); Ta-cadherin (Ta, placozoan); and Mm5-cadherin (Mm5, mouse). The amino acid sequence of Pt1-cadherin is duplicated; one of the duplicates is placed at the top as a reference to show the domain subdivisions. The “-” character indicates introduced gaps. All residues of each cadherin sequence are shown, although some parts of the sequences were aligned poorly or not at all. Excluding the reference sequence, the amino acid sequences derived from different exons are distinguished using arbitrary background colors to indicate the exon-exon junctions in the transcripts. (PDF 385 kb)

Additional file 10: Figure S8. Conserved cysteine residues in the EC domains of classical cadherins. Alignments were generated from the EC5-EC6 (A), EC7 (B), EC7-EC8 (C), EC13 (D), EC14 (E) and EC17 (F) regions of type III cadherins and the corresponding regions of other classical cadherins. The cysteine residues are shown in red. The “-” character indicates introduced gaps. The classical cadherins shown are as follows: DE-cadherin (DE, fruit fly); Tc1-cadherin (Tc1, beetle); Am1-cadherin (Am1, honey bee); Ap1-cadherin (Ap1, aphid); Dp1-cadherin (Dp1, water flea); Le1-cadherin (Le1, sea slater); Sm1-cadherin (Sm1, centipede); Sm2-cadherin (Sm2, centipede); Cm-cadherin (Cm, shrimp); Le2-cadherin (Le2, sea slater); Dp2-cadherin (Dp2, water flea); Am2-cadherin (Am2, honey bee); DN-cadherin (DN, fruit fly); Pt1-cadherin (Pt1, spider); Pt2-cadherin (Pt2, spider); Ct-cadherin (Ct, polychaete); Lg-cadherin (Lg, snail); LvG-cadherin (LvG, sea urchin); Bf-cadherin (Bf, amphioxus); Pn-cadherin (Pn, fish); and Mm5-cadherin (Mm5, mouse). (PDF 41 kb)

Additional file 11: Table S5. Sources of genomic, mRNA and protein sequences of classical cadherins used in the present study. (XLSX 14 kb)

Additional file 12: Multi-fasta format file of the nucleotide sequences of reconstructed transcripts for Mma1-, Mma2-, Sm1-, Sm2-, Ea1-, and Ha1-cadherins and the potential third *C. multidentata* classical cadherin, and their predicted protein sequences. (TXT 79 kb)

## Abbreviations

CE: Cysteine-rich epidermal growth factor-like domain
CP: Cytoplasmic domain
EC: Extracellular cadherin domain
LG: Laminin globular domain
ML: Maximum likelihood
NC: Non-chordate classical cadherin domain
PCR: Polymerase chain reaction
RNA-seq: RNA sequencing
TM: Transmembrane domain
WGS: Whole genome shotgun sequencing
