# Supplementary material for: Evolutionary origin of type IV classical cadherins in arthropods
Source: BMC Evol Biol. 2017 Jun 17;17:142. doi: 10.1186/s12862-017-0991-2 (PMC5473995; doi:10.1186/s12862-017-0991-2)
Supplement: Supplementary file 1 — Statistics and accessions of RNA-seq data from P. tepidariorum, L.exotica and C. multidentata. Table S2. Expression levels of selected transcripts from P. tepidariorum embryos, as indicated by RNA-seq. Table S3. Statistics and accessions of WGS data from P. tepidariorum, L.exotica and C. multidentata. (PDF 91 kb) [file 12862_2017_991_MOESM1_ESM.pdf]

Table S1. Statistics and accessions of RNA-seq data from *P. tepidariorum*, *L. exotica* and *C. multidentata*

Species	*P. tepidariorum*	*L. exotica*	*C. multidentata*
**BioProject Accession (ID)**	PRJDB4545 (311027)	PRJDB4544 (311026)	PRJDB4543 (311025)
**BioSample Accession**	SAMD00046546-SAMD00046547	SAMD00046538	SAMD00046540
**Source of RNA**	Embryos (stages 5 and 10)	Late embryos	Adult muscle and neural tissues
**Sequencing statistics**			
Total number of reads	76,311,240	63,443,171	84,031,433
Total length of reads	10,943,180,468	9,056,941,780	12,908,834,365
Average read length	143	143	154
**Assembly statistics**			
Number of contigs	110,557	111,125	137,038
Average length of contigs	504	474	517
Maximum contig length	12,239	16,790	17,619
Minimum contig length	151	151	151
**Sequence Accession**	IACA01000000 (IACA01000001-IACA01110557)	IABZ01000000 (IABZ01000001-IABZ01111125)	IABX01000000 (IABX01000001-IABX01137038)

Table S2. Expression levels of selected transcripts from *P. tepidariorum* embryos, as indicated by RNA-seq

Source of RNA		Stage 5	Stage10
Total number of reads		42,509,216	33,802,024
Read count for transcript (RPKM) [Gene accession]	*Pt1-cadherin* [AB190303]	36 (9.0×10^{-2})	164 (5.2×10^{-1})
	Pt2-cadherin [LC110189]	11,145 (2.4×10)	4,083 (1.1×10)
	HistoneH3 [AB433909]	13,884 (2.1×10^{2})	5,633 (1.1×10^{2})
	armadillo [AB120624]	18,522 (1.2×10^{2})	4,155 (3.5×10)

RPKM, Reads per kilobase of exon per million total reads

Table S3. Statistics and accessions of WGS data from *P. tepidariorum*, *L.exotica* and *C. multidentata*

**Species**	*P. tepidariorum*	*L. exotica*	*C. multidentata*
**BioProject Accession**	PRJDB4545 (311027)	PRJDB4544 (311026)	PRJDB4543 (311025)
**BioSample Accession**	SAMD00046541	SAMD00046536	SAMD00046539
**Sequencing statistics**			
Total number of raw reads	267,313,642	119,027,276	172,640,984
Total length of raw reads	62,469,669,171	27,104,689,929	38,906,241,771
Average read length	234	228	225
Estimated coverage	31×	8×	13×
**Assembly statistics**			
Total sequence length		953,555,114	1,948,953,281
Number of contigs		1,663,556	2,750,712
Maximum contig length		15,258	19,406
Minimum contig length		301	301
Contig N50		573	819
Contig L50		480,226	642,049
**Sequence Accession**		BDMT010000000 (BDMT010000001-BDMT011663556)	BDMR010000000 (BDMR010000001-BDMR012750712)
