# Supplementary material for: Evolutionary origin of type IV classical cadherins in arthropods
Source: BMC Evol Biol. 2017 Jun 17;17:142. doi: 10.1186/s12862-017-0991-2 (PMC5473995; doi:10.1186/s12862-017-0991-2)
Supplement: Supplementary file 2 — Characterization and subdivision of the amino acid sequences of DN-, Pt1-, and Pt2-cadherins. A. Alignment of the EC1-EC17 regions of DN-, Pt1-, and Pt2-cadherins (abbreviated as DN, P1 and P2, respectively). The “-“character indicates introduced gaps. Conserved hydrophobic residues (blue), Ca2+-binding motifs or residues (red), and XPXF motif sequences (green) are aligned, all of which represent structural features of EC domains as shown schematically at the top. Thick blue arrows denote the seven β-strands (βA to βG). Each red arrow indicates the inter-EC linker to which the Ca2+-binding motif or residue belongs. No residues are omitted from the alignment, except for three sections where 7–12 residues of the DN-cadherin sequences are placed outside the alignment (parentheses). The N-terminal sequence (Nt) preceding the EC1 domain is also shown for each cadherin. B. Alignment of the NC and subsequent domains of the DN-, Pt1-, and Pt2-cadherins. In both A and B, conserved cysteine residues are highlighted in pink, and the residues bordering the start and end of the introns are highlighted with yellow and green. (PDF 362 kb) [file 12862_2017_991_MOESM2_ESM.pdf]

A

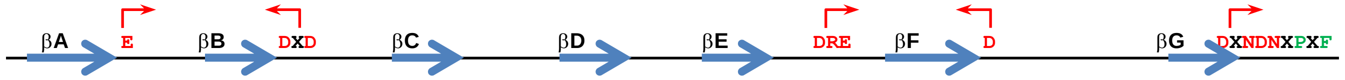


```
DN/Nt                                                                                    MAARRCLNQLRQRYITNRFNICTCAIFLISLP

DN/Nt   FILAIEETTFAGLSAENAARMLAGSPGDVEKSSLSHHSEMSLVLPHDTYPGFSIKKFKTHPVKINGSSHSGAAAYHMLDTDYSKYFTVLEDGVVMTTADISPLVNRPVQLVVVEQTPNATNTHNLQLFVMHRNDMLRF
P1/Nt                               MAGSKRLIRILAVCCCLLSSSCLADVLRVAVPHDAYPGYGITRLDCWGQAFSLLENEFSSLFAVMSDGLLMPTSELSHLIDSPPLTLVVRQEAQNGTKEQMIVIHVVDRNRLVRF
P2/Nt                                      MIHFSRLLTLIFLITPAFAVVKVSVPHDIQPGYSIKKLKSHGRIHLLEDESSPHFTPLQDGSLISTSNISVLAGSKVDLILKEELHDETLIHPITIYVRHRFHMLQF

                                                              DN/EC2  (LVINGDDESGVW)
DN/EC1  SGSLLDASGEVRENQPAGTRVRGVPLMQAFSGSILDEELATPKKVRYTIIDGNVDDAFALQERKANKNIQISAKS()LVTNRPLDREERAH---YDLSVEASD----------VDGLDRTVSKIQITVLDENDNRPIF
P1/EC1  SQDS-Y-SGQIYENEPAGTVVEGLDRMFATSDPY--------HPIIYTFVSGNEEDAFSIVQLPINNLSS----SIVIRTNRVLDRERQEV---YDLVIRATD----------VQEANSADAQVTIQVNNVNDNAPVP
P2/EC1  SKPE-Y-HGYIFENEPPETAVAGLSDLAVYND----------VGMVNYSLSGSSR--FAVVRHKN---------SISIVSTKKLDRESKAT---YELLLNATD-----------SLGDVAIATIKIAVKDVNDNPPIF

         DN/EC2  (QKSASMESNSSV)                                                                                              DN/EC2  (EKRRVTR)
DN/EC2  KS-LDY-KFAIAG()TYQRFAIMGKVEATDAD------GDKIAYRLKSPS-------NVVIIVPQ-----TGEIMLAGEPTSNELLIEVIAHDLRYPSLVSAKPAKVLLEFLAAEPVSFIMQHLEHDDINNHSHHR()
P1/EC2  ER-TIY-EFRIPE--DTELYSIVGNVSAYDAD------GDKPVYHLV-SR------HPVFAIIPK-----TGQILLIAAPELKTYKLNVRTQDTAKPPRISAPFSVYIEVYS---EELPLESENVDSNDIQLLSRQKR
P2/EC2  RR-RNY-NFNVPL--DTPKHSKIGLIRAVDAD------GDKPVYSFLNSK------SKEFVIVPK-----TGEVLLMSDPKIGVYNLFVQAHDNRIPMLYSDAIPVVIRVGN------LVSEENVTP-----HRRSKR

DN/EC3  AVRPTK-RIEFTEADGDTEGKSVFQLEKETDKE---------TFKIRDDNPW--------VTVET-----NGA----VRVKKKWDYEELGPEKTIDFWVIITN------MGHNAGIKYTDNQRVIILVKDVNDEPPYF
P1/EC3  SVRPTK-SYEYKETDGSKPGKVMFQLDKKHPQE---------TYKMENPIKW--------VDVDS-----SGD----VKIKEPWDYEQLGKEKTIDFWVFVTG------PNIND----PERQRVIIHLKDVNDEPPYF
P2/EC3  SIRQTRTYEHFLESDASMPGKVMFRLESIRPDE---------IFTLENGSRW--------IDVDH-----NGD----VKVKEPWDYEQLEREKTIDFWVQIKA------PQQPE----PDRQRIIIHVQDANDENPYF

DN/EC4  INRPLPMQAVVQL--NAPPNTPVFTLQARDPD-----TDHNIHYFIVRDRTGG------RFEVDER----SGVVRTRGTDLFQLDME-------YVLYVKAED-----QNGKVDDRRFQSTPEERLSIVGGKR-APQF
P1/EC4  INRPLPMQAVVQL--NAPPGTPVFKLQARDPD-----TDHNIHYFLVRDRTGG------RFEVDER----SGEVRTLGSDPFMLDKE-------YVLYVKAED-----HNGMTEDRQHQSTGEERLSIMGGKR-PPQF
P2/EC4  INRPMPMQAVVQL--NAQPGTSVFKLQARDPD-----LDSNIHYFLVRDRTGG------RFEVDEM----SGEVRTVGGDPFMLDQE-------YVLYVKAED-----LNGIKVTGKEQSTGEERLSIIGGKR-PPQF

DN/EC5  YYMPSY-EAEIPE--NQKKDSDIISIKAKSFA------DREIRYTLKAQGQGAG-----TFNIGPT----SGI----VKLAKELDFEDLRQPHVYSLIVTATE----------DSGGFSTSVDLTIRVTDVNDNAPKF
P1/EC5  -YMQKY-EATIPE--NQKKDSDIIEVKAKSFA------DREIRYTLRAQGKGAG-----TFNIGPT----TGV----VKLAKDLDYEDLRQPKSYSLIVTATE----------DSGGFSTSVELTIKVTDVNDNAPRF
P2/EC5  -FMQSY-SASIPE--NKKKDSEIIQVKAMSFA------NRQIHYILRTKGQGAG-----TFIIGPT----DGI----VKLAKDLDYEDLRQPKKFSLQITATE----------DSGGFSTNVELNIDVTDVNDNPPRF

DN/EC6  EL-PDYQAHNVDE--DIPLGTSILRVKAMDSD---SGSNAEIEYLVSDD----------HFAVDS-----NGI----IVNNKQLDADNNNA--YYEFIVTAKD---------KGEPPKSGVATVRVYTKNKNDEEPKF
P1/EC6  EL-PDYQAHNVDE--DVAVGTSILQVSASDMD---QNKNAEIEYSLDKE----------DFTIDN-----KGV----IYSNKRLDADINNT---YVLTVRATD---------RGDPPLTGTATVRIYTENKNDEPPKF
P2/EC6  EV-PDYQAYGVPE--DAPIGTSILQVSATDTD---SGKNADIEYSLDKS----------DFTIDA-----RGV----IYSNKRLDADIVNT---YELAVRAVD---------RGEPPLTGTATVRVYTENRNDESPKF

DN/EC7  SQ-QVY-TPNVDE--NAGPNTLVTTVVASDKD------GDNVRFGFVGGGTSSG-----QFVIEDI----TGV-IRLHNKAISLDKD------KYELNVTAMD--DGSCCVNGDQTIHTSTAVVVVFITDVNDNKPVF
P1/EC7  SQ-DVY-TPNVDE--NAGPNTLVTTVVASDKD------GDNIYFGFVGGGTVSG-----MFQIEER----TGV-IRLISGHIQLDKD------KYELNVTARD--DGACCKNGALTTHTSTALVVVFITDVNDNKPLF
P2/EC7  SQ-DVY-TPSVDE--NAGPNTLVTTVVASDID------GDNVVFGFQGGDTVAG-----MFEIEER----TGV-IRLINGQIHLDKD------KYELNVTAKD--DGACCKNGERTLHTSTALVVVFITDVNDNKSVF

DN/EC8  KDCSTY-YPKVEE--GAPNGSPVIKVVATDED---KGVNGQVKYSIVQQPNQKGT----KFTVDEE----TGE----VSTNKVFDREGDDG-KFVSVTVKATD---------QGDPSLEGVCSFTVEITDVNDNPPLF
P1/EC8  EECSTY-APQVEE--GAPSGTQVIKVKAIDND---KGHNGQVRYSIVQQPNQKGT----KFIVDEV----TGD----IRTNKVFDREGDDG-RFVSVTVKATD---------RGNPPLEGVCSFKVEITDINDNPPLF
P2/EC8  ENCDNY-NPKVEE--NADPGTFVIRVEATDQD---KGYNGQVRYSVVQQPNQRGT----KFIVDEI----TGD----VRTNKVFNREGEDG-RFVSVTLKATD---------RGNPPLEGVCSFKVEITDVNDNPPLF

DN/EC9  DR-QKY-VENVKQ-DAS-IGTNILRVSASDED---ADNNGAIVYSLTAPFNPN---DLEYFEIQAE----SGW----IVLKKPLDRE------TYKLEAMAQD--------KGYPP-LSRTVEVQIDVVDRANNPPVW
P1/EC9  DR-QEY-RENVKQ-DTP-KGTNILRVSASDED---ADNNGAIVYNLTAPYDPT---DLDYFEINPD----SGW----ISLRKALDRD------QYHLRAIALD--------KGLPQ-HRATVEVIIDVVDRANNPPIW
P2/EC9  HR-SEY-HENIKQ-DTP-VGSNVLRIAASDED---ADNNGVVVYSLFPTSS-E---YRGYFSINPE----SGW----ISLKKSLDRE------KYFLRAEATD--------KGVPP-MKASVNVILEVVDRKNNPPVW

DN/EC10 DH-TVYGPIYVKE-NMP-VGGKVVSIKASSGI----EGNPTVFYRLMPGSTAQTN-KFHTFYLQQR--PDNGDTWADIKVNHPLDYE---SIKEYNLTIRVEN---------NGAQQLASEATVYIMLEDVNDEIPLF
P1/EC10 DQ-PVYGPIFIKE-NYM-VGQVVASIKARSGI----PDNPTVFYTLMKGSTEQTN-KKDTFYLDQR--NLNGDTWADIQVNYPLDYE---RIQQYNLTVRVEN---------NGIQQLASEATVYIVLEDVNDEIPLF
P2/EC10 DQ-PVYGPISLKE-NIQ-PGQKVAALKARSGI----PDNPRVFYTLIKGGTEQTN-GKDTFYLSQY--SEGGSTWAGIYVNYVLDYE---KVQQYNLTVRVEN---------NGAQQLASEATVYIVLQDVNDEIPLF

DN/EC11 TE-RE--QETVLE--GEPIGTKVTQVNAIDKD--GTFPNNQVYYYIVDSARNEG---KEFFEINLQ----SGE----IFTKTVFDRE---KKGAYALEVEARDGAPSARPN--SNGPNSVTKFIRIGIADKNDNPPYF
P1/EC11 IE-RE--QETVLE--GMPPGTKVTQVQASDKD--GTYPNNKVYYAIESKDQGD-----KFFTIDRE----TGE----IYTRVEFDRE---EKQAYAILVRAEDGAASDRPNMKPGEPNSVTKYIRIGIGDKNDNPPYF
P2/EC11 VE-QE--QETVLE--GMPIGTKVTQIRAVDKD--GTYPNNKVYYSIEDKDDGE------KFRIDRE----TGE----IFTKEIFDRE---ERQSYVVIAKAVDGNPSARPNVPEGEPNSVTKYIRIGIGDKNDNPPYF

DN/EC12 DK-SLY-EAEVDE--NEDIQHTVLTVTAKDHD-----ESSRIRYEITSGNIGG------AFAVKNM----TGA----IYVAGALDYE---TRRRYELRLAASD------------NLKENYTTVIIHVKDVNDNPPVF
P1/EC12 DQ-ALY-EAEVNE--DEDIQHTVITVTAKDKD-----ESSRIRYEITQGNIGG------AFAVKNE----TGA----IYVAGPLDYE---SRKEYKLRLVASD------------NLNENHTTVVIHIKDVNDNPPMF
P2/EC12 EQ-ASY-EAEVNE--DEDIQHTVITVTARDKD-----ESSRIRYEITAGNIGG------AFAVKNE----TGA----IYVASPLDYE---TRKSYNLTLVASD------------TLFENSTTVIIKVKDINDLPPKF

DN/EC13 ER-PTY-RTQITEEDDRNLPKRVLQVTATDGD-KDR--PQNIVYFLTGQGIDPDNPANSKFDINRT----TGE----IFVLKPLDRDQPNGRPQWRFTVFAQD---------EGGEGLVGYADVQVNLKDINDNAPKF
P1/EC13 DR-PTY-ETQITEEDDRNLPKRVLQVTATDGD-RDR--KPDIVYFLTGQGVDDQDPANSKFAINTT----TGE----IYVLKPLDRDLPHGRSQWRFTVFAED---------EGGNGLVGYADVLVNLKDINDNSPFF
P2/EC13 TQ-SLY-QTHILEEDSDGLPKRILKVYATDGD-LDR--NSEILYFLTGQGIEDK-VEDSKFAINDT----SGE----IYVLRPLDRDLPHGRSQWRFTVYATD---------EGGKGLVGYADVLVNLKDINDNAPFF

DN/EC14 PQ-GVY-FGNVTENGTAGMV--VMTMTAVDYDDPNEGSNARLVYSIEKNVIEEE-TGSPIFEIEPD----TGV---IKTAVCCLDRE---RTPDYSIQVVAMD-----------GGGLKGTGTASIRVKDINDMPPQF
P1/EC14 PY-AIY-TGNVTENGTAGMT--VMTMTATDYDDPNEGMNARLKYSIEQNQVNEN--GELIFTIDEE----TGV---ISTAVCCLDRE---TNPEYTIKVVAMD-----------GGGLKGTGTATIKIKDINDMPPEF
P2/EC14 PQ-AIY-TGNVTENGTAGMT--VMTMTATDYDDPNEGSNAKLKYTIEQNQVNEN--HELIFAIDQD----TGV---ITTAVCCLDRE---AISEYTIKVVATD-----------GGGLQGTGTATIKIKDINDMPPVF

DN/EC15 TKDEWFTEVDETDGTA-LPEMPILTVTVHDEDE-----TNKFQYKVIDNSGYG----ADKFTMVRNNDG-TGS----LKIVQPLDYEDQLQSNGFRFRIQVND----KGEDNDNDKYHVAYSWVVVKLRDINDNKPHF
P1/EC15 TKKEWQVEVDETEGDN-IPETPILVVSVNDGDLLE---TNRFSYKVIDNAFG-----ADKFTMVTNSDG-TGS----LKIAKPLDFEDLQQRFGFNITIQVSD-----HGGESSEPYHVDYAKVRVRLRDINDNKPEF
P2/EC15 TKKEWYVQVDETEGDD-IPELPILVVSVKDEDLLE---TNRFSYKVINNTFG-----ADKFTMVTNSDG-TGS----LKIAKPLDYEDPMQRYGFNITIQVSD-----HGGETTNAYHIDYSKVFIRLTDINDNPPEF

DN/EC16 ER-ANVE-VSVFE-DTKVGT-ELEKFKATDPD---QGGKSKVSYSIDRSS-----DRQRQFAINQ-----NGS----VTIQRSLDRE---VVPRHQVKILAID---------DGSPPKTATATLTVIVQDINDNAPKF
P1/EC16 EK-PNIE-VIVKE-DADIGT-SLATFRATDAD---QGGKSRVSYTIDRSS-----DKKRQFRIDQ-----NGV----VRIQRTLDRE---ETPRHQVKILAID---------DGTPPRTATATLTVVVSDINDNAPRF
P2/EC16 SE-PLLR-ASVEE-NVTVGY-VIKKFQARDPD---QAGKSMVKYSIDRSS-----DKKRQFKISQ-----LGV----VTIQRPLDRE---TVNRHDIRILATD---------DGTPTRTATATLIVDVIDVNDNPPHF

DN/EC17 --LKDY-RPVLPE-HVPPRK--VVEILATDDDDRSKSNGPPFQFRLDPSADDII---RASFKVEQDQKGANGDGMAVISSLRSFDRE---QQKEYMIPIVIKD---------HGSPAMTGTSTLTVIIGDVNDNKMQP
P1/EC17 --LRDY-RPVIME-HSPPQK--VEEILATDDDDRSKGNGPPFTFRMDPNAPDII---KQFFDVQHDPTGANGDGMAIVTSKERFDRE---QQKEYLVPIVIKD---------SGTPSMTGTSTLTVIIGDVNDNRMHP
P2/EC17 --REDY-RPVVME-NVEPPV-KVIEIFADDPD-LAPNTKPWFKFRLDTDADDTI---KNSFRVVFNKDGDNGKGSATVYTKVKFDRE---QQKEYHVPIVIAD---------SGVPSLTSTNTLTVIIGDENDNVMYP
```

B

```
DN/NC   GSKDIFVYNYQGQSPDT---PIGRVYVYDLDDWDLPDKKFYWEA-MEHPRFKLDEDSGMVTMRAGTREGRYHLRFKVYDRKHTQTDIPANVTVTVREIPHEAVVNSGSVRLSGISDEDFIRVWNYRTQSMSRSKMDRF
P1/NC   GSKSIFVYNFKGESPPT---PIGRVHVEDLDDWDLPDKSFYWENNIAHPNFELDKDTGLIVMKNVTSGGTYFLRFLVHDRVHTQ-EVSANVSVTVKEIPEEAIYNSGSIRISGISAEDFVRVWNWTENRQVKSKYHKF
P2/NC   GEKDIFVYSYQGATKPTRPVPIGRVHVEDQDDWDIPDKVYYWKDNQQHQNFDLNVETGEISMIELISGGKYTLHFTVIDQKRNE-EVPATVTVTVKEIPEEAVFSSGSIRIAGHSDEDFIRIWDWKNKVQVESKYNKF

DN/NC   RDKLADLLNTERENVDIFSVQLKRKHPPLTDVRFSAHGSPYYKPVRLNGIVLMHREEIEKDVGINITMVGIDE
P1/NC   RDMIASLTTTRKDNVDIFSVILKQERPPITDVRFSAHRTPYFKASMLNGIVALNRELIEQEVGINITMVGIDE
P2/NC   REILAKLLKVKKDNVDIFTVLKHQDHPPMADIRFSAHGSPYYKASRLDGLIGLHRKNIEQIVGVNITMVNIDE

DN/CE1  CLYENQMCEGSCTNSLEISP-LPYMVNANKTALVGVRVDTIADCTCGARNFTKPESCRTTP--CHNGGRCVDTRFGPHCSCPVGYTGPRCQ
P1/CE1  CFFENINCETSCTNILMVDR-QPIVVNANRTSFVGVNTWVQPKCVCGARDFSEIETCRKRPLPCHNGGRCFESYGGVSCKCLDGYEGPQCQ
P2/CE1  CLYEEK-CEDSCTNYLRVYGDRPATVNANRTSLVGVRAQIEAECTCGARDFTSMESCKTRPKPCYNDGICSDKFGVINCTCSEGFNGPQCQ

DN/LG1  QTTRSFRGNGWAWYPPLEMCDESHLSLEFITRKPDGLIIYNGPIVPPERDETLISDFIALELERGYPRLLIDFGSGTLELRVKTKKTLDDGEWHRIDLFWDTESIRMVVDFCKSAEIAEMEDGTPPEFDDMSCQARGQ
P1/LG1  MTTRSFGGRGWAWFPSLQQCENSHLSLEFMTRKPDGLLLYNGPISNPDIGEVAVQDFISLELQSGRPRLLIDFGSGTAEIVVYVDESLSDGEWHQIDIFWDRETVRLVIDNCQKAKI---EDSDPPKINRSRCETKSN
P2/LG1  KTTRHFGGDGFAWFPPLQQCETSHFKLEFMTQSQNGLLLYNGPISEADSNEKIVQDFFSLELQNGKPRLLIDFGSGTTEIIIDTGSSLSDGGWHQLDVFWDRETVRMVVDNCMSARN---QDVDPPVSNHAMCEGTAA

DN/LG1  IPPFNEYLNVNAPLQVGGLYREQFDQSLYFWHYMPTAKGFDGCIRNLVHNSKLYDLAHPGLSRNSVAGC
P1/LG1  IPPFNEFLNVNTPLQLGGVHHQYLTD--YRWQHQHTREGFEGCIKNVIHNSEMYDLGNPGSSSASSPGC
P2/LG1  IKPFNEFLNVNGPLQLGGVNHPVLSS--YKWNFAHTRKGFNGCIKNVIHNSEMYDLANVGESQDSTMGC

DN/CE2  PQTEEVCAQTETTARCWEHGNCVGSLSEARCHCRPGWTGPACNIP
P1/CE2  KPADDACQSNSITRQC-EHGTCVGTYNSAKCTCYPGYFGSRCDKE
P2/CE2  PPAEENCYINQVERFC-KHGTCVGNYTSAKCVCFPGYHGERCNIK

DN/LG2  TIPTTFKAQSYVKYALSFEPDRFSTQVQLRFRTREEYGELFRVSDQHNREYGILEIKDGHLHFRYNLNSLRT-EEKDLWLNAIVVNDGQWHVVKVNRYGSAATLELDGGEGRRYNETFEFVGHQWLLVDKQEGVYAGG
P1/LG2  TISKMFQQNSYIKYALSFEPDPYKTDIQLQFRTRQKHGELFRATSKHGREYCILEIRDKKLRLRFNLNHLRSSDEHELWLPNVQVSDGQWHTVRVLRHGSTASMSVDGGGGRRYNELLEYEGQHQLMMIEKQNVIAGG
P2/LG2  TQEKMFEQNSYIRYALTFNPGEYSNEIRLSFRTRQKQGELFRATSKHGREYLVLEIRNRTLRFRFNLNHYKS-QEQELWLPDVSVSDGQWHSVKVQRFGSTASISLDGGGGRRYSELLGYQGFHQELVIEKQNVIAGG

DN/LG2  KAEYTGVRTFEVYADYQKSCLDDIRLEGKHLPLPPAMNGTQWGQATMARNLEKGC
P1/LG2  DVQYVGPGVTVVDNDFQEGCMNDIRLDQRYLPME---NGSENAAVLEWRNLINKC
P2/LG2  DVQYLAPGVTIVDNDFQEGCLDDIRLDSRYLPME---NGSQSAIVLDSQNVKSGC

DN/CE3  PSNKPCSNVICPDPFECVDLWNVYECTCGEGRIMSPDSKGCMDRNECLD-MPCMNGATCINLEPRLRYRCICPDGFWGENCELVQEGQTLK
P1/CE3  PSNNPCQGIICPKPFICLDLWMLHECRCPEGFTITEDGKNCTDANECLS-DPCLNGGSCVNLPNGEGYYCVCPDGFGGLYCGARHEEKVMR
P2/CE3  PSNNPCLGVYCPPPFVCVDLWMIYECTCPKGFIVTPDRKDCIDDDECVSQQPCLNGGTCVNLPDGQGYECICGAGYFGMHCNAVREEKKMQ

DN/TM   LSMGALAAILVCLLIILILVLVFVV
P1/TM   LSMAALAAILICLLNILILVLVIVA
P2/TM   LSVAAMALIIFCIFAVLILALVVMA

DN/CP   YNRRREAHIKYPGP--DDDVRENIINYDDEGGGEDDMTAFDITPLQIPIGG----PMPPELAPMKMPIMYP-VMTLMPGQEPNVGMFIEEHKKRADGDPNAPPFDDLRNYAYEGGGSTAGSLSSLASGTDDEQQEYDY
P1/CP   YTRNRRPNQKYGHGDVDDDVRENIISYDDEGGGEDDMNAYDITPLRIPIDAATGTPLGAK--PEKPIIKDNRQRQLPPGTD-GVGDFIRDHLDKADNDPNAPPFDDLRNYAYEGCGSTAGSLSSLASGTEDNEQDFDY
P2/CP   YSRFRSP-KPYDQKDIIDDVRENIIVYDDEGGGEDDMNAYDIKTLQIPIDG-YGSPIGAKSGPEKGPIREPHQWQAQPGVQPDVGDFIRDHQDKADTDINVPPFDDLRNYAYEGGGSSAGSLSSLASCYDDNDHDFEY

DN/CP   LGAWGPRFDKLANMYGPEAPNPHNTELEL
P1/CP   LNGWGPRFQKLADMYGPGESEED
P2/CP   LNGWGPRFQKLADMYGQGESEEE
```
