# Supplementary material for: Evolutionary origin of type IV classical cadherins in arthropods
Source: BMC Evol Biol. 2017 Jun 17;17:142. doi: 10.1186/s12862-017-0991-2 (PMC5473995; doi:10.1186/s12862-017-0991-2)
Supplement: Supplementary file 3 — Alignment of the entire amino acid sequences of thirteen type III cadherins in arthropods, and comparison of the exon-intron organizations. The alignment was produced using the ClustalW algorithm without manual adjustment. The classical cadherins shown are Pt1-, Pt2-, Mma1-, Mma2-, Mo-, Sm2-, Cm-, Le2-, Ph2-, Ha2-, Ea2-, Dp2-, and DN-cadherins. The domain organization is indicated above the Pt1-cadherin sequence. Blue lines with breakages indicate exons, and the breaking points indicate intron insertion sites revealed by comparisons with the corresponding genomic sequences. (PDF 5991 kb) [file 12862_2017_991_MOESM3_ESM.pdf]

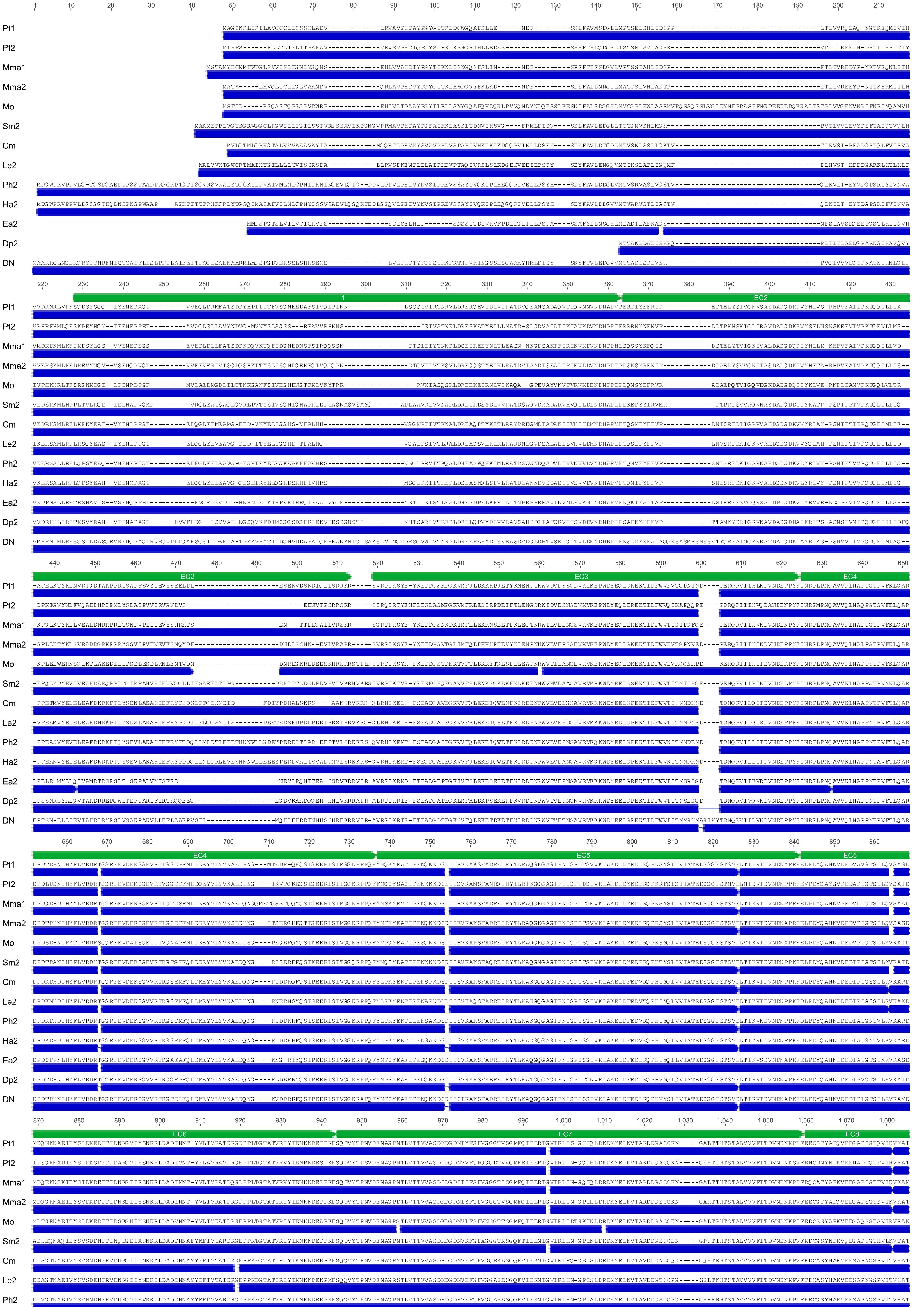
Pt1
Pt2
Mma1
Mma2
Mo
Sm2
Cm
Le2
Ph2
Ha2
Ea2
Dp2
DN
EC2
EC3
EC4
EC5
EC6
EC7
EC8

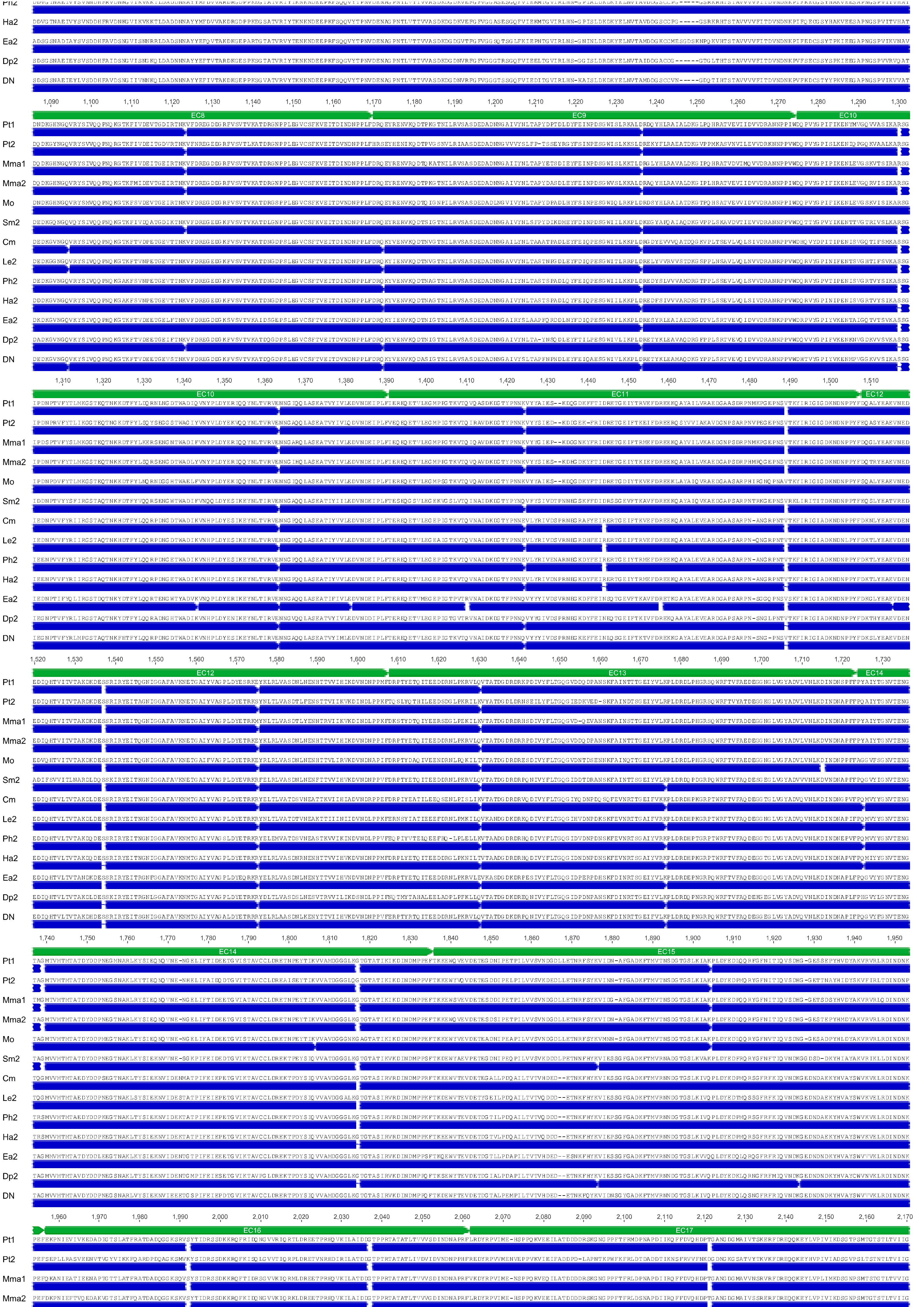

Pt2
Ha2
Ea2
Dp2
DN
EC8
EC9
EC10
EC11
EC12
EC13
EC14
EC15
EC16
EC17
Pt1
Pt2
Mma1
Mma2
Mo
Sm2
Cm
Le2
Ph2
Ha2
Ea2
Dp2
DN

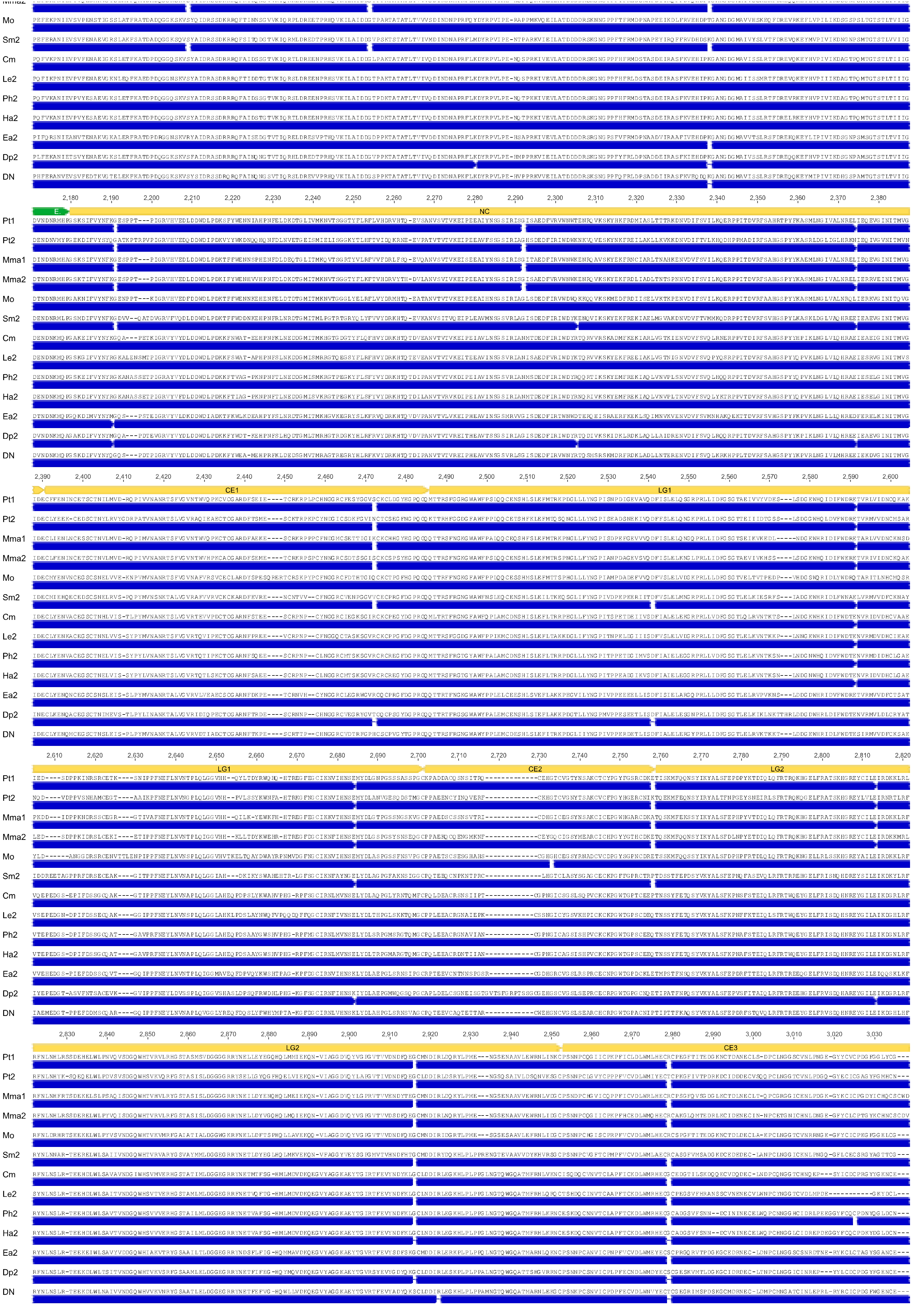
Mma2
Mo
Sm2
Cm
Le2
Ph2
Ha2
Ea2
Dp2
DN
E.
NC
CE1
LG1
CE2
LG2
CE3
Pt1
Pt2
Mma1

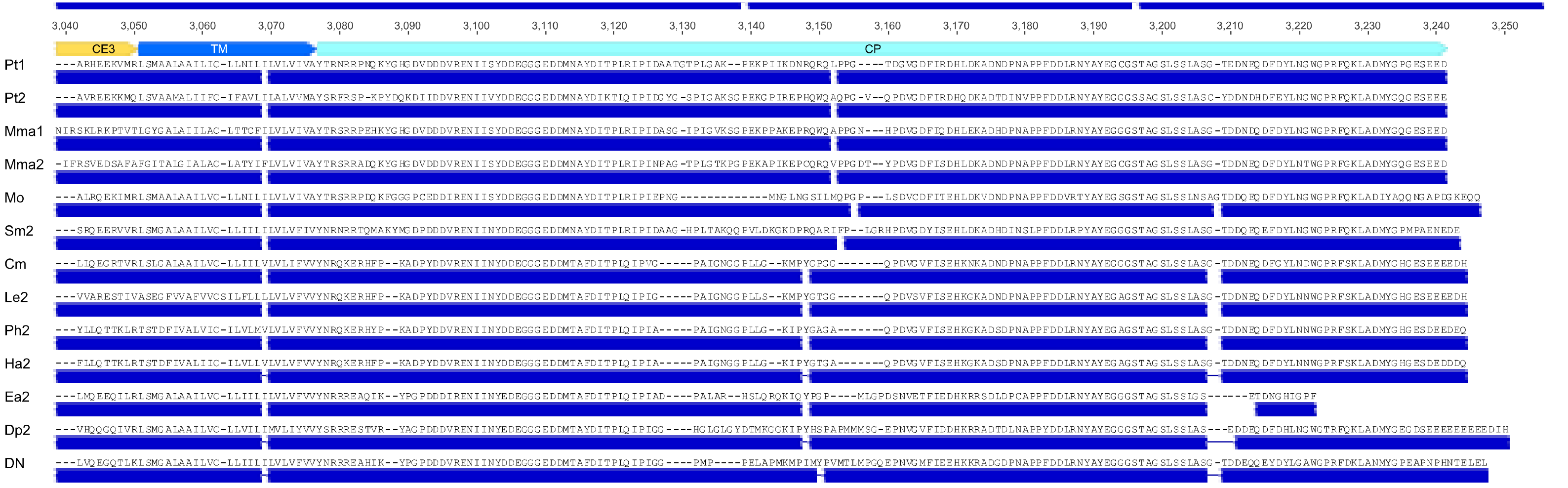
3,040
3,050
3,060
3,070
3,080
3,090
3,100
3,110
3,120
3,130
3,140
3,150
3,160
3,170
3,180
3,190
3,200
3,210
3,220
3,230
3,240
3,250
CE3
TM
CP
Pt1
Pt2
Mma1
Mma2
Mo
Sm2
Cm
Le2
Ph2
Ha2
Ea2
Dp2
DN
