# Supplementary material for: Evolutionary origin of type IV classical cadherins in arthropods
Source: BMC Evol Biol. 2017 Jun 17;17:142. doi: 10.1186/s12862-017-0991-2 (PMC5473995; doi:10.1186/s12862-017-0991-2)
Supplement: Supplementary file 4 — Characterization of the amino acid sequences of type IVa and type IVb cadherins. A. Alignment of the amino acid sequences of all EC domains (EC1-EC7 or EC1-EC9) of the DE-, Dp1-, Ea1-, Le1-, Ha1-, and Ph1-cadherins (abbreviated as DE, D1, E1, L1, H1, and Ph1, respectively). Conserved hydrophobic residues (blue), Ca2+-binding motifs or residues (red), and XPXF motif sequences (green) are aligned. Thick blue arrows denote the seven β-strands (βA to βG), and each red arrow indicates the inter-EC linker to which the Ca2+-binding motif or residue belongs. No residues are omitted from the alignment, except for three instances where 5–7 residues from the Le1- or Ha1-cadherin sequences are placed outside the alignment (parentheses). The N-terminal sequence (Nt) preceding the EC1 domain is also shown for each cadherin. B. Alignment of the amino acid sequences of the NC and subsequent domains of the DE-, Dp1-, Ea1-, Le1-, Ha1-, and Ph1-cadherins. In both A and B, the conserved cysteine residues are highlighted in pink, and the residues bordering the start and end of the introns are highlighted with yellow and green. (PDF 379 kb) [file 12862_2017_991_MOESM4_ESM.pdf]

A

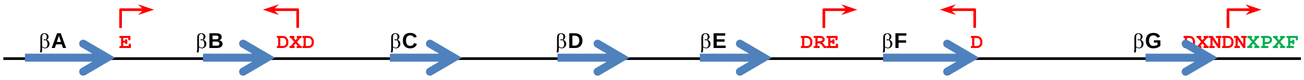


```
H1/Nt                                                                                                                 MASFSSNGSPVDARERQQRVVAA
H1/Nt    HTTEKLYFPHVPLFGNEKPRSHAFIALPLSSNAINPDFKINRSFEPSSPGSLQSLPQGLENPKSALNYHNCSSDADNAGGRYSFSLSHVDEDYAFHQPPETVDVNSEIRDSVSLNLNNRNDKNNEAWGLTKRKS

L1/Nt                        MELIKRMLAVISAMLLSDVYEAESLLPTISRSKNSSNFQSYFAKPHQLHLTKNSRSFGDFEGDFPLFSLKEIDKSAELFPLADFPYNFRKVRHRHNEDSRFPRNTRNLHEPRF
H1/Nt    KISRLHSSDKHAPGSSSFKHSGGSKHPRKHSTHPGKHHDQKRQRNKPHNEGTGRHRRHYQRNRHHEKAVWKNFDTAPFRQPDDSTRPEASISRNQTRAEWPIERYHADAPSVSSVRDSSWSGQQASGNMHLPKF
Ph1/Nt       MTKKKIYSTAIGENNFEVNHKLAKHKLHNLKKHGRGHHKNRRHKDGGLNTRHRRHNRHKVSSGWYTPNFDTSPFRVSGSSVSPEYERTTLTKDPEIYNTQNDGPVVMGVRERPAVLKSSSTNEHLPQF

L1/EC1   -THSEY-HRTVDENVQPGT-SILTVEATDDD---LDEYGLIRYEVEGTN--------AFAVDE-------GGV--VYTNSRLDYESSGG--VYDFYVVANN------PSAS-SDGTSVPIRIQVRDTPD-APVF
H1/EC1   -RQKSY-KATIDENAPVSS-SVMSVYATDDD---IDENGIVEYVSSDS---------HFGVDH-------SGL--VYVKEPLDYESSGG--RYMFLIYARN------PGTAMPMWAQVPVSVVIRDTRE-APEF
Ph1/EC1  -RQARY-DAVVKEDTPVGT-SIVTVYATDED---KNEHGIVEYVSSDS---------HFSVDR-------GGV--VHVRTPLDYESSGG--RYQFLVYARN------PGTARTMWAQAPVTVVIRDTRE-APEF

DE/Nt                                    MSTSVQRMSRSYHCINMSATPQAGHLNPAQQQTHQQHKRKCRDLGRRLIPARLLLGVIVAISLLSPALALHSPPDKNFSGDNRKPAF
D1/Nt                                       MKKMRCCLVTTTVALLLITYVYADVLSESHHVTTRQATGSGRNNRVTDGTFSTPGQQELERSGSPRSNANKRVYVENKYRPVF
E1/Nt                              MGRERTLSKGLVLVRSSVLILSFLVLSTSSFARRSSSSKYLDKSLNPDSSFQSSRHSRWRRMPRPNPEALSDSETLLLLTQENQVDQVANLYKPEI
L1/EC2   -NSDKY-YFNISELATDGD-YVGTVVARDSD------NDLESYSLQNVSPEDL-----FEIDEH------NGI--ITVQ---NRESGTDYV-YEFQAVATD---------ATGSTATVPVVVNVIDENTNKPIF
H1/EC2   -DSPSY-NFAISEFSLQGE-YVGRVIAWDPE------RSLVTYSLAMVSPPNH-----FVVDAA------SGI--ISVG---ERGGSDKFS-YTFQAVATD---------EEGASSTVPVSVKIIDENTHPPVF
Ph1/EC2  -DSPSY-NFAISEFARQGE-YVGRVIAWDPE------KTSVTYNLSGVNPANS-----FTVDPA------SGI--IWVG---TRSDVTKYT-YQFQAVATD---------EEAAYSTVPVTVNIIDENTHAPGF

DE/EC1   KNCAGY-APKVKEEQPENT-YVLTVEAVDPD---PD--QVIRYSIVQSPFERP----KFFINPS------TGV--IFTTHTFDRDEPIHEKFVFVTVQATD-------NGLPPLDDVCTFNVTIEDINDNAPAF
D1/EC1   YQCESA---TLEEGKQPGT-VVSTVRAYDND---TDSAGEIFFTILRAGSEK-----EFEVDPV------TGT—-IRSSKSFDRDEPAREKIAYVTVKATD-------RGTPPLEAICTFNVTIGDINDNAPMF
E1/EC1   LNCDEK--PSVKEESQIDT-AVVQVQAVDKD-DPSSSAGRVVFSIVSTHQ-------KFKINPT------TGW--ISTNKIFNRDEPDREKEVHVTVKASD-------KGRPSLEDVCTITVVIEDINDNSPIF
L1/EC3   DNCSGYNNLKVKENQTVPQ-DILITTATDAD---HGKNGYVEYSLLTDDP-------EFAIDPV------SGQ--LTVQQPLDRDNGLKEHFLTVIAK--D-------MADDPLQSSCSFTIIVTDINDNPPVF
H1/EC3   TECQSYQNFMMKENQTDPA-NVIIVKATDRD---QGVHGQVTYSFLGNIE-------DFVIDER------SGQ--ISTTAPLDRDMGRKEYFMTVVAR--D-------GGSPPLEGSCSFEVILEDINDNVPVF
Ph1/EC3  NECQSYQNFSFKENQTAPA-NVIIVKATDAD---HGPHGVVTYSFLGDVP-------DFQIDER------SGQ--IYTTAPLDRDKGVKEYFITVVGR--D-------GGTPPLEGSCSFEVILEDVNDNAPVF

DE/EC2   -NKARY-DESMSENAQPDA-VVMTISASDFD---DGNNSLVEYEILRER-----DFQYFKIDKE------SGI--IYLKRPIDKR---PGQSYAIIVRAYN------VVPDPPQDAQIEVRIRVVESSIKPPSF
D1/EC2   -DKSAY-DESVPRDLELGK-LVMRIAATDVD---AGENAHISYGWDAGSGP---DAEYFDLDKS------TGA--IHLKKKITQN---EGYKFALMAVARD------NGTEP-MNSRVRVMINVASSKIKPPYF
E1/EC2   -DRASY-DIPIAQDTKEGT-PIMRVSATDID---EGDNQRITYEFQASQYQQ--DIEYFRWDDK------TGV--VTLNKKIDKP---VGSVFLLTAYAKD------GGIPP-RSSQTAVTIDVKESNNKPPSF
L1/EC4   -DLESY-SQTVPSDTEENS-PILRLTASDDD---AGANAEIVYSLEGSDE----DLEYFSVDRD------LGY--IILQKPLTQS-MANEREFIFKARATD----VGEPDQEPLTSTVDVVLSVVSPGSMPPTV
H1/EC4   -DQPSY-SQDIPADSETHV-NLLRVTASDAD---SGVNAVIQYSISGGDD-------YFTIQPD------SGY--IKLVRPLSED-MANVRAFSLSVQAKD------MGTPA-LSSSASVRINVVGSDQVAPSV
Ph1/EC4  -DQPSY-LQNIPVDSETHV-NLIRVTASDAD---SGVNAELVYSVSGDEG-------YFMIEPD------SGH--IKLIKPLSEE-MANVKKFSLAAIATD------KGSPA-LSSSANVRINVVSSDQMAPSV

DE/EC3   -VNPIDTPIYLKENLKNFTHPIATLRAVSNM----PDKPEVIFELNTGRTEQTNSKNTFVFNQI-----GNEVT-ISLGKTLDYE---AITDYTLTMIVRN---------THELGTEHQIKIQVEDVNDNIPYY
D1/EC3   -SDIP-EPVSLPENYTSYKTAIATLGAHSNI----AA-PDLYFELVKGQTEQTNSDETFRINEL------GNSVNIHVAKALDYE---KVTQYILTVRVKN---------EAGVAAETVLTVNVEDVNDEIPTF
E1/EC3   -RQGPGAEIELSEGTVDWNTPIVSYTADSQI----PGDPTLFFELVNGRTEQTNKDTTFRAVQD---ISNQQRVIIYLAKPLEFE---KVQAYTLTLQVRN---------SQDLVAEAQLNIKVKDENNKSPVF
L1/EC5   -ENQNPELAQVNEDATLNT-NIVSICASSNS----ED-NSVYMDMINGNVPETNSEGTFDIRTGTSE()TIGGNIFLATQNLDFE---EIQSYTLIIQLAN----------NNGRAEYSLTVDVIDVNDNPPVL
H1/EC5   -VERDP-SPQVRENATIGT-IVAQLCATSNT----DNRDDVYFTLFAGNTPSMNGDGTFDIRK--DG()TVGAVIYVAVANMDYE---VITAYKLIVQVSN----------SNARIDHSLTVRVLDVNDEMPLL
Ph1/EC5  -VERDP-SPQVHENATVST-PVAQLCATSNT----DNKQDVYFTLFAGNTPSMNGDGTFDIRK--DG()TVGAVIYVAVANLNYE---KIKSYKLIVQVSN----------SNARIDHSLTVRVVDVNDEMPLL
                                                              L1/EC5  (ETGCPSN)
                                                              H1/EC5  (TASCPLN)
                                                             Ph1/EC5  (TASCPPN)

DE/EC4   ---TEVKSGTILENEPPGT-PVMQVRAFDMD--GTSANNIVSFELADN-------REYFTIDPN------TGN--ITALTTFDRE---ERDFYNVKVIASDNSPSSLFDNGEPNRGHQVFRISIGDKNDHKPHF
D1/EC4   ---MAVDGGSVLENSPANT-PVLTVQATDRD--ATYPNNWVRYELRDF-------KDKFEIDRN------TGD--IKTKVKLDRE---ERKSYPLTVVAFDGKESALTLDGLPNQSAKRFQIEVADVNDNAPFF
E1/EC4   ---TNIESGAVLEHEPAGT-VVMSVSAIDGD--GTYPNNRVKYSISDSNPEL---LSKFDINPD------TGV--ITTKVEFDRE---ETAYYALTIIASDGATSSLLKNNKPNQTPQKFRIVIKDKNDNPPYF
L1/EC6   ---QQPYTGSIGENVD-ST-MVMVVQAVDKD--ITPEFRQLEYSFK-DPPDHV--TDNFEIDPV------SGE--IWNTKPLDRE---SEKQYRIPVQVSDGVN--------VRDRQ--YWIIVNDENDEPPKF
H1/EC6   ---QHPFSGVISENSDPNS-LIMTATAIDKD--VSPEFKQLRYSIKADAPVEV--RTNFRVDPN------TGD--IWTLHTLDRE---KKRSYGVPIEVTDGAP--------GHNRHNLYWITINDVNDEPPTF
Ph1/EC6  ---QHPFSGVISENSEPNS-LIMTVTAIDKD--SSPEFKQLHYSIKADAPPEV--RQNFRVDPR------TGD--LWTLQTLDRE---KKRNYGVPIEVTDGAP--------GHNRSSLYWITINDVNDEPPAF

DE/EC5   -QQDKYLAERLLEDANTNT-EVIEVKAEDED-----NASQILYSIESGNVGD-----AFKIGLK------TGK--ITVNQKLDYE---TITEYELKVRAFD----------GIYDDYTTVVIKIEDVNDNPPVF
D1/EC5   -PQSEY-FAEIAENADIGA-KVSELTALDND-----TESQLTYDIVSGNAGL-----VFSIEAQ------TGL--LKVHQPLDYE---TTKSYDLIVEVDD----------GKQKATTKVNVKIINVNDNKPQF
E1/EC5   -PQQTY-RAEVPEDADIGS-KVIEVKAQDLD----KEASLTTYSIESGNLNN-----AFKIEEQ------TGF--IRVNTELDYE---TIREYTLRVQAWD----------GQYSNDTTVHIDILNVNDMKPRF
L1/EC7   NTELGVYETHVDEDMTIGKDTGIKLVVEDKD-----ILNEFSYEIIGGNDAN-----KFKINAE------TGS--IYVNDNLDYDSPVNDRNFTIDVRVAD----------GKSFDTTQVRIIVRNVNDLAPLF
H1/EC7   NLNLGVYNVMIPEDLEIGKDTGIQLVVDDLD-----DVNEFNYQIISGNEAD-----KFAIDAK------TGT--VTVNKKLDYDSPVNDRMFEMSVTVSD----------GRSFDTTGIKISVTNVNDLRPVF
Ph1/EC7  NRDLGAYDVSIPEDLEVGKDTGIQLVVDDLD-----DVNEFNYQIISGNEAD-----KFAINSR------TGE--VTVNKRLDYDSPVNDRLFRMSVTVSD----------GRNYDTTEISINVTNVNDLRPIF

DE/EC6   --KQDY-SVTILEETTYDD-CILTVEAYDPDIKDRNADQHIVYSIHQNDG------NRWTIDN-------SGC--LRLVKTLDRDPPNGHKNWQVLIKAND-----EDGVGTTVSTVKEVTVTLKDINDNAPFL
D1/EC6   MNSNPEQIHGIVENTVPLG-PIVRVNATDPDYDPSTADGPMKITYTLSGSH----ADSFQINQ-------LGE--LSIVKPLDRDLPEGRRDWSVFVVAQD------ELDGVRLENTLEVVVVLDDVNDNAPFL
E1/EC6   -KEEKY-VTTLKENFVPSY-PIFSVQAEDPDIGDPSVPQNITYFLDQTAQI----APHFQISS-------DGA--LRIVKPLDRDLPDGFPVWTMFVFAKD-----ENGGPNGRESFVEFEITLDDVNDNAPFL
L1/EC8   -NPKMY-EAIVRENTECNI-TVTQVYAYDPDYPTNG---SIIYSLSRSE------QSNFTIDPI------EGT--VKVKGCMDREAAPLG-MLTIYPIAFD------EGGEGKNSEPATVEITIEDENDNHPFI
H1/EC8   -LPHTY-NISVVENQECDV-QKVQVRAYDPDLPAEQEDQGIRYYLDLEQ------TRNFTIDRM------TGW--VSVRGCLDREATFGG-TEIINPRADD------GGGKGINAEPGVINVNIIDVNDNHPYI
Ph1/EC8  -SPSTY-NVTVTENTECDV-SKVQVKAYDPDLPPDQEDQGIRYYLAVEQ------TRNFSLDRM------TGW--FSVVGCLDREAAHGG-VEIVNPRADD------GGGKGINAEPGIINVTILDLNDNHPFI

DE/EC7   --INEM-PVYWQENRNPGH--VVQLQANDYD--DTPGAGNFTFGIDSEATPDI--KTKFSM---------DGDY-LHANVQFDRE---AQKEYFIPIRISD-------SGVPRQSAVSILHLVIGDVNDNAMSE
D1/EC7   --EQTR--VVWRENQPQGR--IVVLSATDYD--EPKNGPPFAMKMADTAEDIV--RTSFRI-----DGSPTSSWSLMATKTFDRE---ERKEYAVPIVISD------SGQPTSLTATSTLTVVIGDENDNPMSD
E1/EC7   -NMPDG--LVWPENSPPGE--VGRLEALDYD--TPANGPPFTFKIDPKAPASF--KAKFGI-----KKKSNGDYYLETKEIFDRE---ELKVYEIPIEVAD---------NQGLKAVSFLRLIIGDKNDNPMES
L1/EC9   -QSPPDQYTQFPENVSPED()VVVIRLSDQDDSSMGNGCPCTLAFDESTPPNV--FESFDVI-----ENGENDYKLVTTKTLDRE---AQKYYVIPFRTTD---------SQGLSGVRELTVEVGDQNDSPMTD
H1/EC9   -KVPDNSRARVRENLPPANVPPIPILLDDAD--APEHGCPCTLEFHHDVDPLM--LTLFRVE----RDSVNNKYQLIVQETLDRE---KQKRYLVPFVTSD---------SQNVSGLRHLTIEVDDVNDNPMTD
Ph1/EC9  -KYPDNSRARVRENLAPSQVPPIPIQLDDAD--TPEHGCPCTLEFDPDVDPVL--TNLFEVR----RGSAPNTYQLYVRETLDRE---RHKMYLVPFTTRD---------NEGVSGRRYLTIEVDDVNDNPMTD
                 L1/EC9  (MSTST)
```

B

```
DE/NC    GSSRIFIYNYKGEAPETDIGRVFVDDLDDWDLEDKYFEWKD--LPHDQ---FRLNPSTGMITMLV----HTAEGEYDLSFVVTEDSMFVP-RHSVDAYVTVVVRELPEEAVDKSGSIRFINVTKEEFISVPRDFQ
D1/NC    GSSSILVYNYRNSLPDTEIGRVYVQDADDWDLPDKTFGWSTTVTPPPY---FTVTALGGYITMRAR----TPPGNYPLKFHVTDNIRQEE----ADATVIVTVQEITEEAVRRSGSIRLSGIGQEEFIKDSP---
E1/NC    GSSEIYVYNYMGRAPTTEIGRVYVDDPDDWDLPDKSFFFKDP-SLFPD---FELNRDNGIISMKEGIKLDEEIKVYHMEFRVEDPVHQQTGPNAVYANVTVTVQRIPKEAVTKSGSIR-LDISPEKFISAE----
L1/NC    GESKITVYNYRGSFNRIIIGSVYVTDADDYDVADKTFKIDTKETATEDISHFQVDPDNGNITMLRL----TPAGDYLLVVNVTDNARKEE----AKGIVHITVIELPKEAVMESGSFLIQDYSVSEVITQES-LD
H1/NC    GSGTITVYNYEGKYPDIVIGNVYVTDADDHDSGDKTFEVDP-MTAADAEEQFQVKKETGNITMKAG----TKAGTYTLFVRVTDHARSET----AIGRQVIQVVTLPERAVRQSGSFIVAGTTAHDMVNNN----
Ph1/NC   GTSSITVYNYRGKFPDTVVGSVYVTDADDHDISDKTFEIDP-TTPNNAEKQFLVDKNSGNITMLAG----TAAGTYTLVVKVTDNSRGET----AVGQQTIHVVTLPERAVRQSGSFIVKDHTAHSFVGKD----

DE/NC    SPDALSLKDRFQLSLAKLFNTSVSNVDVFTVLQN---ENHTLDVRFSAHGSPYYAPEKLNGIVAQNQQRLENELDLQMLMVNIDE
D1/NC    -----SKKDLLRQKVAEYVGAKEENVDVFTVMSSSPTRNHSVDVRYSAHGSPYYDPVKLNGLTAAHQDELERILDAEVLMFGIDE
E1/NC    -----DGREKLTKLLRGYLNPNASIVDVFTVLPAG-PNGEFTDVRFSAHGSPYFAPEKMEVTVARRKMDLERTLGFKILMVHIDE
L1/NC    VYEGTSLYDLLKVEFGRIFDELPENVDIFYLQDVERRGFSGVDVRFNIHGSPYYTSSHVNGLLMQHKDEVENNLGIKIPVIDDNL
H1/NC    -GNGPSLYQRLRTRLASVFGVATENMDVFSITEAEG----GVLVRYNCHGSPYFTEPRLNGILLSKKSEVEKDLGIVIPLVDTDK
Ph1/NC   -AIGSSLYSRLRASLAEIYNVETDNMDIFSLTDVEG----GVLVRYNCHGSPYFTRARLVGLLLRHRSQIESDLRITIPMVNTDK

DE/CE    CLIE-KFKC-EESCTNELHKSSV-PYMIYSNTTSFVGVNAFVQAQCVCEAPLM--------------------RRCLNGGS--PRYGENDVCDCIDGFT---GPHCE
D1/CE    CLHEKNSICSEGSCTNRLQITEQ-PYRVATNTSTFVGVDARIVPECVCLADEYLSSS----TCS--------ATSCLNGGTCTGDSVQ--PCRCPDGFQ---GPRCE
E1/CE    CLYE-GTKC-EGSCYNSLEIENN-PTAIMTNTTTFVGVTARVEPRCGCPVDKAFQ------TCSAGFEAPDAGTECLNGGTC-----DGRQCKCPAENSPEFGPQCE
L1/CE    CLYEDFQSCNGTSCQQELRAENNNPLVLESETSTIVGVRVTEKYTCNCGYLDPQPR-----ACPEGFE-----NYCYNGGVCNYTSAGGLSCTCLDDYNY--GPRCE
H1/CE    CLYESLSPCSNSSCNMELRINSTHPAVFGSGNSTVVSIQVDEYYACTCSGDHHFHGEDDGDLCE-----------CLNGGKC-LSSSNPTACTCPDKVNY--GPKCE
Ph1/CE   CLYESMSPCANSSCSMELRINATHPAIIRNEDATVVSIQTEEYYACTCADTNIAYGRQDGIDCN-----------CLNGGTC-PDASNPSLCKCPDGLNY--GPKCE

DE/LG    LVSVAFYGSGYAFYEPIAACNNTKISLEITPQIDQGLIMYLGPLNFNPLLAISDFLALELDNG--YPVLTVDYGSGAIRIRHQH--IKMVADRTYQLDIILQRTSIEMTVDN--CRL-------STCQTLGA--P
D1/LG    GLDVSFGGTGWAWYDPLPTCASGFLSLTVVTQTGNGLILYAGPTAVPPDTSVTDFLALELREG--SPVLYLDLGSGTRRLELPDRSRNLVDGKAHDLEISWNQRSVQMRLGQ--CSD-----GQPYCSASSS--L
E1/LG    KLSASFR-YGWTTQRGVSACANTSLSFMFTTKQQEGLLLYQGPSPNTVVEGVTDFFAVELQEG--KLKYFLNFG-ASTQIGVVP--KVVSDGEEHHVQISWTNSSVSVILDNGECIP-----NIRECQLQNSR-P
L1/LG    LLTGRFK-NGFAWFDTLGTCEEPTLHITFQSDSSSAVLLYNGPVIASPYEYYPKDFLYIVLNN-WVVEAYLNLGSETSRVYVPVD---EGESEPYDVYLSWTKSTVSLIIPN--CGLNITEEASEACKRTLNL-P
H1/LG    LLSARMN-KGFSWFSPLDTCERSSLSLSFESSEEAGILLYNGPIVAKPYFEYPKDFVYIYFDNTPKVVAYLELGTGTMVLSVPFS---QVQDVRVTVVLEWGNGGVTLTVRN--C------SATDPCEDSKPLLG
Ph1/LG   LLTARMV-EGYAWFSSLDTCETSTLSISFESEKDEGILLYNGPIISKPYAEFPKDFVYMYFDSTPKVVAYLELGSGTMMLSVNIS---AEGSERKTAQLEWNSKGVSLTVLN--C------GLAEPCRDSKLLAG

DE/LG    IGPNEFLNVNAPLQLGG---TPVDLEQLGRQLNWT--HVPNQKGFFGCIRNLTINEQTYN----LGMPSVFRNIDSGCQQS
D1/LG    VGSNEYLNVNGPLQVGG---ITADLQKLRLALKWD--YIPTDIGYSGCIQNLTFNGLTYN----LFDPGFFKNATPSCAG-
E1/LG    QGKSQFLNTNGPLQVGGLYFGPERLRELAAELGLTREELPGGTSYGGCIRELRISEGGASRYVSLGTPSDGENYQDGCSME
L1/LG    ESATSILNAGGPLQIGG-LAPMIPLEEIGDSFEWN-LNLPNVEGLSGCISYLEYNGEIYD----LNRTTYNKYFYRTCEKT
H1/LG    DSPSFLFNTGGPLQLGG-MASMPSFATLAQSYGWS-VTPKSMPYFTGCLSSLQFNDQFYD----LNATDYFANFHPTCAEV
Ph1/LG   DSPSFLFNPGGPLQLGG-MVSMPSFSTLAQNYGWT-QTPVGKAYFTGCFSNLKFNGQYYD----LNSTDYRKNFYGTCSEV

DE/TM    VAVAFS-FGIDRNFIIAIIVCLALLLIILLAVVV
D1/TM    VVLPVQTGAFGWEIIIIILGILLLLMIIVLVLVA
E1/TM    YVLAVEATGMNLNFLIIIILVILAIILTAVVVLAM
L1/TM    VVTSRMMMSSESVLIIVGSLLVLLLLVLFVLCLL
H1/TM    KMEAVVQLGAESIVIIIVSLLLLLMLLLLLLLLA
Ph1/TM   KVGAVVQLGTESIIIIIISMLLLLLLLILLLLLA

DE/CP    QKKQKNGWHEKDID-DIRETII-NYEDEGGGERD-TDYDLNVLRTQP--FYEEKLYKDP---HALQGN-----MRDPN-DIPDIADFLGDKKENCDRDVGATTVDDVRHYAYEGDGNSDGSLSSLASCTD--DGDL
D1/CP    YRRRSRKDKPKDFHDDIRENII-NYSDEGGGEGDMTGYDLSVLRMTP--DGKPLIGRSDDYGKLLKDEDL-RPKRAAPGQVPDISQFLDDNKGRVDRDPDGLAYDDLRHYAYEGDGNSMGSLSSLASGTD--DGDL
E1/CP    YRRRRVHYNDKDIDCDIRENII-NYEDEGGGEGDQTGYDLSVLRMMSGENGTPMLHSKD---KIPAPQ-----HRPPVGEVPGVEDFLDETKDRMDQDPEATPYDDLRHYAYEGDGNSGGSLSSLNSGSDA-DEDL
L1/CP    RRRKKPHSYPDHLNDIVKETIG-TTDIEGYGEKDMTQFDLKLLRVTPEGKLLNGYGTKGDLPDVAKGHSPKKAPLARLSEGFNVEDFIDDNITKVDKEHP--DFDDVRHYCFEGDEMSIASLSSLGSGSYDDDGP-
H1/CP    RRSKRQPSYPDHL-QFVNQTMG-ASNLEGFEEKDATKFDLNMLRVTPDGQLLSAEKVPRAMPDVTYTEPLSRAPLAHIPEGWCVGDYIEDSLQKLHSEPE--SLDNVRHYCYEGDDMSIASLSSLSSGTSDGDQPA
Ph1/CP   RRSKRQPSIPENL-HFVNQTVGPTSNIDGFEEKDATKFDLNMLRVTPDGQLIAAEKTPRVLPDVTNNEPLTRAPIAVLPDGLSVGDYIHDCLKKLSSEPE--CLDNVRHYCYEGDDMSIASLSSLGSGSSDADKPM

DE/CP    NFDYLSNFGPRFRKLADMYGEEPSDTDSNVDDDQGWRI
D1/CP    DFECLSDFGPRFKKLADMYGDHSSESED------------------------------------------------------------------------------------GEDGQQGGQ-----SGSESWC
E1/CP    QFEYLNQFGPRFKKLADLYGRE-SDSEDSQDGMDNMGYYPSSGA-------------------------------------------------------------------NLDPNQPQGQ-----PGSESWC
L1/CP    -YNYKEDWGPRFNRIAEIYGPKPDEE----EDSDYEFPIPKKPPKVVSGGSNPSKLNQITPSSGASSVGSLNTQNSADGSEPSTVPYTRLGQHNSSSSHEEDGKKGGAPPVVFRGEYHEAVNPLA--QSKESWC
H1/CP    HYDEFQEWGPKFERLHQLYGQDNNAE----SDSEFDFPPVPKDAARQYRRARFYQNRPSRQEPA-----------ADLRARSPQPYQRL----------RDIGGGEGP--LYHESVNPLADVYS--GGAESWC
Ph1/CP   QLDDFEEWGPKFERLHHMYGQPEKQTDVESQESEYDFPTISKEETRRYRRARFRPTDQRSALPP------------GSVHGSPQPYQRL------------GGGGDGP--SYNESVNPLADVYDDRNGMESWC
```
