# Supplementary figures and images for: Evolutionary origin of type IV classical cadherins in arthropods
Source: BMC Evol Biol. 2017 Jun 17;17:142. doi: 10.1186/s12862-017-0991-2 (PMC5473995; doi:10.1186/s12862-017-0991-2)

A

Accession

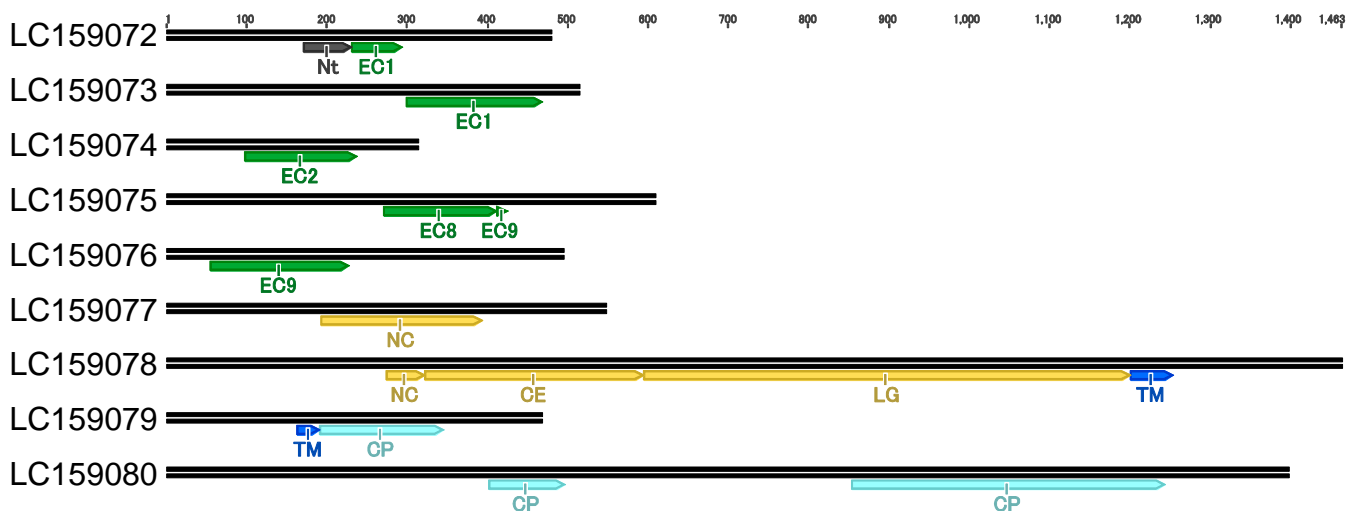

B

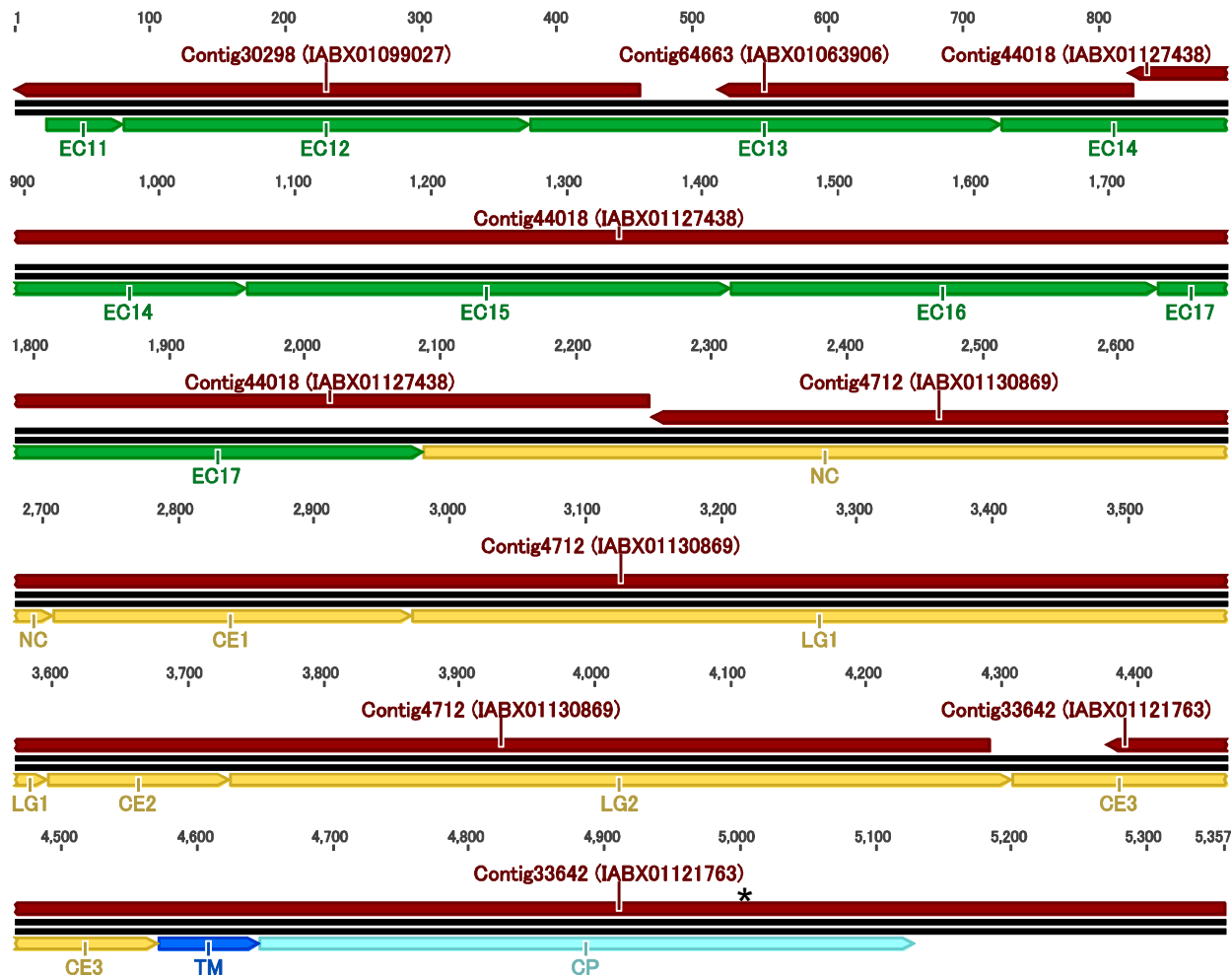

Supplement: Supplementary file 5 — Figure S4. Schematic representation of detected sequences of C. multidentata related to classical cadherins. A. Nine reconstructed genomic sequences of C. multidentata that contain coding sequences closely related to those of Le1-cadherin. The sequences are available under the indicated accession numbers. B. Eight transcriptome contigs connected by raw reads. The sequences of these contigs are available under the indicated accession numbers. Contig33642 was modified by an insertion of 5 nucleotide bases (CCGGA) between the nucleotides 349 and 350 based on assessment of raw reads (asterisk). The assembled transcript and protein sequences are available in Additional file 12. Detected domain elements are shown. (PDF 108 kb) [file 12862_2017_991_MOESM5_ESM.pdf]

# B

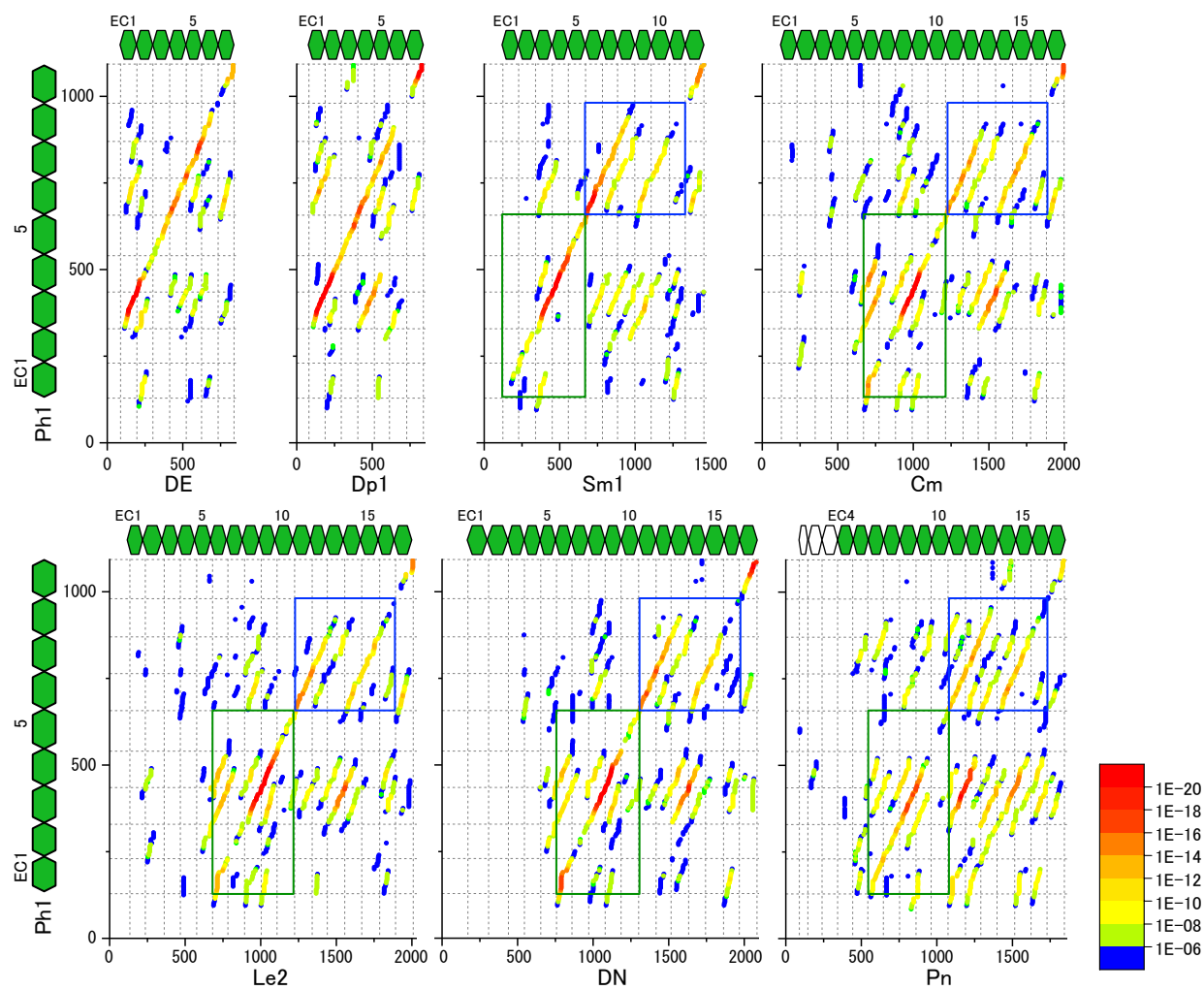

Supplement: Supplementary file 6 — Blast-based dot-plot comparisons between the amino acid sequence of Ha1- (A) or Ph1- (B) cadherin and those of DE-, Dp1-, Sm1-, Cm-, Le2-, DN- and Pn-cadherins. Green boxes indicate comparisons between the EC1-EC5 region of Ha1- or Ph1- cadherin and the EC6-EC10 regions of the type III cadherins or the corresponding region of Sm1-cadherin, which exhibited marked collinear similarities. Blue boxes indicate comparisons between the EC6-EC8 region of Ha1- or Ph1-cadherin and the EC11-EC16 regions of the type III cadherins or the corresponding region of Sm1-cadherin, which exhibited ambiguous collinear similarities. (PDF 2623 kb) [file 12862_2017_991_MOESM6_ESM.pdf]
