# Supplementary material for: Evolutionary origin of type IV classical cadherins in arthropods
Source: BMC Evol Biol. 2017 Jun 17;17:142. doi: 10.1186/s12862-017-0991-2 (PMC5473995; doi:10.1186/s12862-017-0991-2)
Supplement: Supplementary file 7 — Comparison of the exon-intron organizations of type IVa, type IVb and type III cadherin genes. Alignment of the amino acid sequences of the EC1-EC6 region of type IVa cadherins, the EC1-EC8 region of type IVb cadherins, and the EC6-EC13 region of type III cadherins was produced using the ClustalW algorithm without manual adjustment. The classical cadherins shown are DE-, Dp1-, Ea1-, Le1-, Ha1-, Ph1-, Pt1-, Sm2-, Cm-, Le2-, and DN-cadherins. The EC domains for type IVa, type IVb, and type III cadherin are indicated above the DE-, Le1- and, Pt1-cadherin sequence, respectively. Blue lines with breakages indicate exons, and the breaking points indicate intron insertion sites revealed by comparisons with the corresponding genomic sequences. (PDF 1348 kb) [file 12862_2017_991_MOESM7_ESM.pdf]

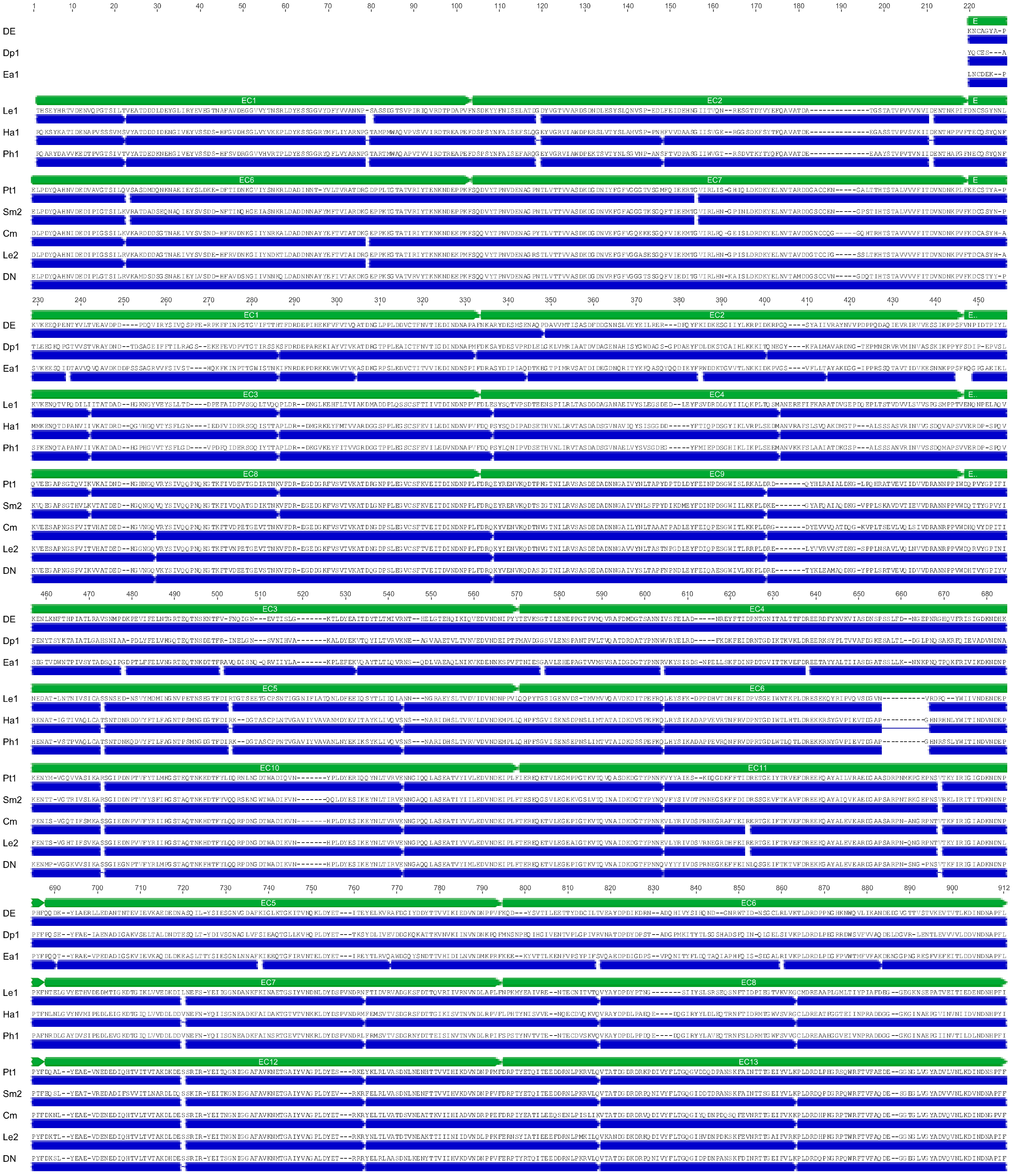
DE
Dp1
Ea1
Le1
Ha1
Ph1
Pt1
Sm2
Cm
Le2
DN
EC1
EC2
EC3
EC4
EC5
EC6
EC7
EC8
EC9
EC10
EC11
EC12
EC13
