# Supplementary material for: Evolutionary origin of type IV classical cadherins in arthropods
Source: BMC Evol Biol. 2017 Jun 17;17:142. doi: 10.1186/s12862-017-0991-2 (PMC5473995; doi:10.1186/s12862-017-0991-2)
Supplement: Supplementary file 9 — Amino acid alignment of selected classical cadherins from arthropods and non-arthropod bilaterians. The classical cadherins shown are as follows: DE-cadherin (DE, fruit fly); Tc1-cadherin (Tc1, beetle); Am1-cadherin (Am1, honey bee); Ap1-cadherin (Ap1, aphid); Gb1-cadherin (Gb1, cricket); Fc1-cadherin (Fc1, springtail); Af1-cadherin (Af1, brine shrimp); Dp1-cadherin (Dp1, water flea); Ea1-cadherin (Ea1, copepod); Le1-cadherin (Le1, sea slater); Ha1-cadherin (Ha1, amphipod); Sm1-cadherin (Sm1, centipede); DN-cadherin (DN, fruit fly); Am2-cadherin (Am2, honey bee); Dp2-cadherin (Dp2, water flea); Le2-cadherin (Le2, sea slater); Cm-cadherin (Cm, shrimp); Sm2-cadherin (Sm2, centipede); Mo-cadherin (Mo, mite); Pt1-cadherin (Pt1, spider); Pt2-cadherin (Pt2, spider); Ct-cadherin (Ct, polychaete); Lg-cadherin (Lg, snail); LvG-cadherin (LvG, sea urchin); Bf-cadherin (Bf, amphioxus); Pn-cadherin (Pn, fish); Ta-cadherin (Ta, placozoan); and Mm5-cadherin (Mm5, mouse). The amino acid sequence of Pt1-cadherin is duplicated; one of the duplicates is placed at the top as a reference to show the domain subdivisions. The “-” character indicates introduced gaps. All residues of each cadherin sequence are shown, although some parts of the sequences were aligned poorly or not at all. Excluding the reference sequence, the amino acid sequences derived from different exons are distinguished using arbitrary background colors to indicate the exon-exon junctions in the transcripts. (PDF 385 kb) [file 12862_2017_991_MOESM9_ESM.pdf]

```
     1        10        20        30        40        50        60        70        80        90        100
Pt1                                               MAGSKRLIRILAVCCCLLSSSCLADVLRVAVPHDAYPGYGITRLDCWGQA----------
DE
Tc1
Am1
Ap1
Dp1
Le1
Sm1
Sm2                                                                           MAVPHDAYPGFAIHKLASSLT----DNVIHSV
Cm                                      MVLGTMLGRVGTALVVVAAAVAYTAMGQETLPEVMYSVAVPHDVSPAHIVHKIKLK---------DGQR
Le2                              MALVVKTGWCRTMAIHYGILLLLCVISCRSDALLRVSDKENPLELAIPHDVPTAQIVRSLSLK---------DGES
Dp2                                                         MTTAKLDALIHHPQPLTLYLAEDGPARKSTHAVQVY-------VVD--R
Am2                                                  MWRALLVVIAALLAATEKVDGDKVVVLPHDVYPGYEVTLFNTK----------RPS
DN     MAARRCLNQLRQRYITNRFNICTCAIFLISLPFILAIEETTFAGLSAENAARMLAGSPGDVEKSSLSHHSEMSLVLPHDTYPGFSIKKFKTHPVKINGSSHSGAA
Pt1                                               MAGSKRLIRILAVCCCLLSSSCLADVLRVAVPHDAYPGYGITRLDCWGQA----------
Pt2                                                     MIHFSRLLTLIFLITPAFAVVKVSVPHDIQPGYSIKKLKSHGR-----------
Ct
Lg
LvG                                                       MAVKLRWNMTRCMCLSAIFLLATLQLTIGLTLPKINVP----------SNA
Bf
Pn                                                MTKAVTGPVILHTLCLLPLLLLHVSPVCLSAQHGHP-----------------------A
Mm5

                                                                             EC1
         110       120       130       140       150       160       170       180       190       200       210
Pt1    -FSLLENEFSSLFAVMSDGLLMPTSELSHLIDSPPLTLVVRQEAQNGTKEQMIVIHVVDRNRLVRFSQD--SYSGQIYENEPAGTVVEGLDRMFATSDPY-----
DE
Tc1
Am1
Ap1
Dp1
Le1
Sm1
Sm2    GFRMLDTDQSSLFAVLEDGLLTTTGNVSHLMGKPVTLVVLEVYPE-FTATQTVQLHVLDSRKMLHFPLT--VLKGEIEEHAPVGMPVRGLEAISAGEG-------
Cm     VIKLLDSEYSDYFAVLDTGDLMTVSKLSSLLGKTVTLKVSTRFADGRTQLFVIRVAVKDRHSMLRFLKP--KYEAFVYENLPPGTELQGLEEMEAMGE-------
Le2    VEEIEPSPYSDYFAVLENGQVMTIKSLAPLIGQMFDLHVSTHFDDGARKLHTLKLFIKERSAMLRFLRS--QYEASIYENLPPGTELQGLEEVEAVGD-------
Dp2    HHLIRFTKSVYRAHVTENAPAGTL---------------VVFLD---------------------------------------GLSVVAENGS-------
Am2    NFRLMENNFSKFFTVLGNGLVMTTSDLSPLVDRPVNLIVVEELAN-ATETHFLHLYVINRRNMITFSHDRLG-EGEVGENRGAGTLVEGFPVLRARGS-------
DN     AYHMLDTDYSKYFTVLEDGVVMTTADISPLVNRPVQLVVVEQ-TPNATNTHNLQLFVMHRNDMLRFSGSLLDASGEVRENQPAGTRVRGVPLMQAFSGSILDEEL
Pt1    -FSLLENEFSSLFAVMSDGLLMPTSELSHLIDSPPLTLVVRQEAQNGTKEQMIVIHVVDRNRLVRFSQD--SYSGQIYENEPAGTVVEGLDRMFATSDPY-----
Pt2    -IHLLEDESSPHFTPLQDGSLISTSNISVLAGSK-VDLILKEELHDETLIHPITIYVRHRFHMLQFSKP--EYHGYIFENEPPETAVAGLSDLAVYNDV------
Ct
Lg
LvG    LPGFRVTEVKKDGQSSELLSDSDI---------HNLFQIAENGA-LEVKNSLKHLANSDIALKIRHTL--RDRSWDDLLNMYVEDSSLEFIKKSYRGYVLESDE-
Bf
Pn     RYFGQIAENSPAGTPVEG----------------VVISVTAANCPGANLTASLKGDYASDFRLVHYRGHR------------------------------------
Mm5
```

```
                                                                                                       EC2
          220       230       240       250       260       270       280       290       300       310
Pt1  -----------------HPIIYTFVSGNEEDAFSIVQLPINNLSSSIVIRTNRVLDRERQEVYDLVIRATDVQEANSADAQVTIQVNNVNDNAPVPERTIYEFRI
DE
Tc1
Am1
Ap1
Dp1
Le1
Sm1
Sm2  --------VRLPVTYSIVSGNIGHAFRLEPIASNASVSAYGAPLAAVRLVVNADLDREIRDSYDLVVRATDSAQVDMADARVHVQILDLNDNAPIFEKEDYYIRV
Cm   ------------------------EDVKYELIGGHSVFALHHVGGMPTIVTKKALDREAQDTYKLTLRAYDREGMDTADAKIIVHIHDMNDHAPVFTQSMFYFFV
Le2  ------------------------EDITYELIGGHDTFALHQVGALPSIVTLRALDREAQSVHKLRLRARDNLGVDSAEAELSVHVLDMNDHAPIFTQSLFYFFV
Dp2  -----------QVKFDIMSGGSDGFRI--EKVTESD-GNCTTNHTSARLVTRRPLDRELQPVYDLVVRAVSAHPPGTATCRVIISVDDVNDNRPIFSAPEYHFEV
Am2  -FPVHYTILADEATGDRPFALRE-----EDSSRTGFNLTLASEKEGVRVVTARPLDREASSAYTVVIHASDGYLLTSTRIDGVVRVLDENDNSPVFEREEYAFEA
DN   ATPKKVRYTIIDGNVDDAFALQERKANKNIQISAKSLVINGDDESGVWLVTNRPLDREERAHYDLSVEASDVDGLDRTVSKIQITVLDENDNRPIFKSLDYKFAI
Pt1  -----------------HPIIYTFVSGNEEDAFSIVQLPINNLSSSIVIRTNRVLDRERQEVYDLVIRATDVQEANSADAQVTIQVNNVNDNAPVPERTIYEFRI
Pt2  -------------------------GMVNYSLSGSSRFAVVRHKNSISIVSTKKLDRESKATYELLLNATDSL-GDVAIATIKIAVKDVNDNPPIFRRRNYNFNV
Ct
Lg
LvG  ANQEVVGLDDLQVNSTKPISYKLAGEDSDDFRLQVGPEGRVHI------FTKAPIDMDTTSQYHLEMVASDLDLSKATAELLIDV--------------------
Bf
Pn   -----------------------------------------VHRGQLCLVSAKALDREFIAMYELMVELPTR-CMRRRA-IVQIEVSDKNDNIPLFTSGNKTIEV
Mm5

         320       330       340       350       360       370       380       390       400       410       420
Pt1  PED-------------------TELYSIVGNVSAYDADGDKPVYHLV-SRHPVFAIIPKTGQILLIAAPELKTYKLNVRTQDTAKPPRISAPFSVYI-EVY---S
DE
Tc1
Am1
Ap1
Dp1
Le1
Sm1
Sm2  MRD-------------------TPRFSVVAQVHAYDADGDDIIYKAT-RPSPTFFTVPKTGEILLIGEPQLKDYEVIVRAHDARQPPLYGTRPAHVHIEVVGGLL
Cm   PLN-------------------ISRFDIIGRVVAHDGDGDKVVYRLA-YPSNTFTIIPQTGEIMLIEPPETMVYELELEAFDKRKPTLYSDNLAKAHIEFRYPSD
Le2  PLN-------------------VSRFDAIGKVVAHDGDGDKVVYQLA-HPSNIFTIVPQTGEILLIDVPEAMVYELELEAHDNRKPTLYSDSLARAHIEFHYPGD
Dp2  PTT-------------------AMKYDRIGRVKAVDADGDHAIFRLT-SASNSFVMIPQTGEIILIDPQLPSSNRSYALQVTAKDRREPGWETEQPARIFIRTKQ
Am2  RPSRRGIGEESGRNSVELVEPGWKRFSTVGKVVAKDADGDKVAYKLV-TPSRLLVVVPQTGELMLVGEPEVGM-DEDGE---CVAMVEAHD----VRNPSRSSVK
DN   AGQKSASMESN-------SSVTYQRFAIMGKVEATDADGDKIAYRLK-SPSNVVIIVPQTGEIMLAGEPTSNELLIEVIAHDLRYPSLVSAKPAKVLLEFLAAEP
Pt1  PED-------------------TELYSIVGNVSAYDADGDKPVYHLV-SRHPVFAIIPKTGQILLIAAPELKTYKLNVRTQDTAKPPRISAPFSVYI-EVY--SE
Pt2  PLD-------------------TPKHSKIGLIRAVDADGDKPVYSFLNSKSKEFVIVPKTGEVLLMSDPKIGVYNLFVQAHDNRIPMLYSDAIPVVIRVGNLVSE
Ct
Lg                                                                                        MATKKCKLLSVCVFILHSIS
LvG  --------------------------------------------------------------------------------------------ATANNQVPPLVKE
Bf
Pn   DEL---------------------TPPGTELARFDAKDGD---------------AESNGHVTFYASPDLYLLHVDPQNGQVSLAGSLAGFSQVTLRLYVKDGGD
Mm5
```

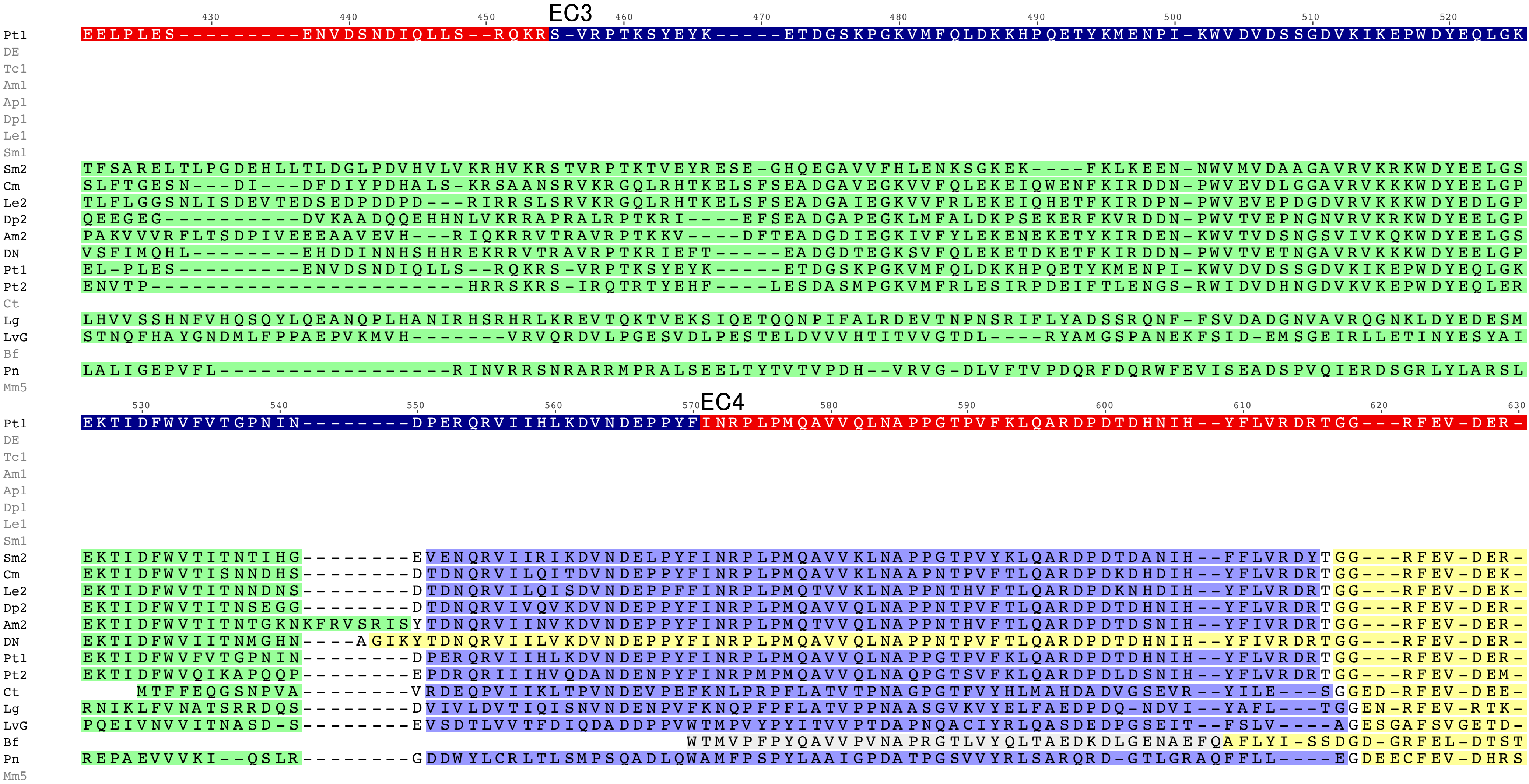
EC3
EC4
Pt1
DE
Tc1
Am1
Ap1
Dp1
Le1
Sm1
Sm2
Cm
Le2
Dp2
Am2
DN
Pt2
Ct
Lg
LvG
Bf
Pn
Mm5

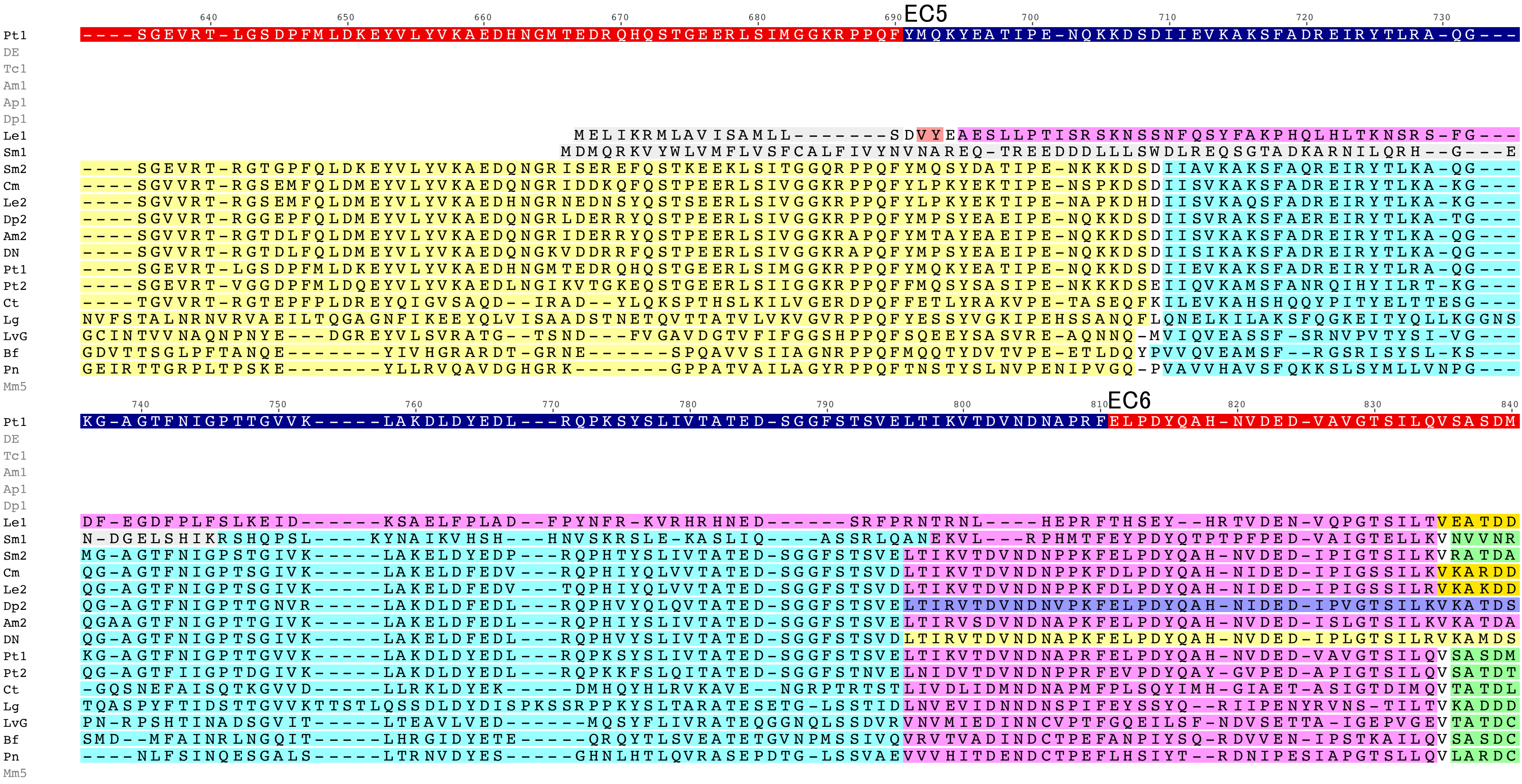
640 650 660 670 680 690 EC5 700 710 720 730
Pt1 ----SGEVRT-LGSDPFMLDKEYVLYVKAEDHNGMTEDRQHQSTGEERLSIMGGKRPPQFYMQKYEATIPE-NQKKDSDIIEVKAKSFADREIRYTLRA-QG---
DE
Tc1
Am1
Ap1
Dp1
Le1 MELIKRMLAVISAMLL-------SDVYEAESLLPTISRSKNSSNFQSYFAKPHQLHLTKNSRS-FG---
Sm1 MDMQRKVYWLVMFLVSFCALFIVYNVNAREQ-TREEDDDLLSWDLREQSGTADKARNILQRH--G---E
Sm2 ----SGEVRT-RGTGPFQLDKEYVLYVKAEDQNGRISEREFQSTKEEKLSITGGQRPPQFYMQSYDATIPE-NKKKDSDIIAVKAKSFAQREIRYTLKA-QG---
Cm ----SGVVRT-RGSEMFQLDMEYVLYVKAEDQNGRIDDKQFQSTPEERLSIVGGKRPPQFYLPKYEKTIPE-NSPKDSDIISVKAKSFADREIRYTLKA-KG---
Le2 ----SGVVRT-RGSEMFQLDMEYVLYVKAEDHNGRNEDNSYQSTSEERLSIVGGKRPPQFYLPKYEKTIPE-NAPKDHDIISVKAQSFADREIRYTLKA-KG---
Dp2 ----SGVVRT-RGGEPFQLDMEYVLYVKAEDQNGRLDERRYQSTPEERLSIVGGKRPPQFYMPSYEAEIPE-NQKKDSDIISVRAKSFAEREIRYTLKA-TG---
Am2 ----SGVVRT-RGTDLFQLDMEYVLYVKAEDQNGRIDERRYQSTPEERLSIVGGKRAPQFYMTAYEAEIPE-NQKKDSDIISVKAKSFADREIRYTLKA-QG---
DN ----SGVVRT-RGTDLFQLDMEYVLYVKAEDQNGKVDDRRFQSTPEERLSIVGGKRAPQFYMPSYEAEIPE-NQKKDSDIISIKAKSFADREIRYTLKA-QG---
Pt1 ----SGEVRT-LGSDPFMLDKEYVLYVKAEDHNGMTEDRQHQSTGEERLSIMGGKRPPQFYMQKYEATIPE-NQKKDSDIIEVKAKSFADREIRYTLRA-QG---
Pt2 ----SGEVRT-VGGDPFMLDQEYVLYVKAEDLNGIKVTGKEQSTGEERLSIIGGKRPPQFFMQSYSASIPE-NKKKDSEIIQVKAMSFANRQIHYILRT-KG---
Ct ----TGVVRT-RGTEPFPLDREYQIGVSAQD--IRAD--YLQKSPTHSLKILVGERDPQFFETLYRAKVPE-TASEQFKILEVKAHSHQQYPITYELTTESG---
Lg NVFSTALNRNVRVAEILTQGAGNFIKEEYQLVISAADSTNETQVTTATVLVKVGVRPPQFYESSYVGKIPEHSSANQFLQNELKILAKSFQGKEITYQLLKGGNS
LvG GCINTVVNAQNPNYE---DGREYVLSVRATG-TSND---FVGAVDGTVFIFGGSHPPQFSQEEYSASVRE-AQNNQ-MVIQVEASSF-SRNVPVTYSI-VG---
Bf GDVTTSGLPFTANQE-------YIVHGRARDT-GRNE------SPQAVVSIIAGNRPPQFMQQTYDVTVPE-ETLDQYPVVQVEAMSF--RGSRISYSL-KS---
Pn GEIRTTGRPLTPSKE-------YLLRVQAVDGHGRK-------GPPATVAILAGYRPPQFTNSTYSLNVPENIPVGQ-PVAVVHAVSFQKKSLSYMLLVNPG---
Mm5
740 750 760 770 780 790 800 810 EC6 820 830 840
Pt1 KG-AGTFNIGPTTGVVK-----LAKDLDYEDL---RQPKSYSLIVTATED-SGGFSTSVELTIKVTDVNDNAPRFELPDYQAH-NVDED-VAVGTSILQVSASDM
DE
Tc1
Am1
Ap1
Dp1
Le1 DF-EGDFPLFSLKEID------KSAELFPLAD--FPYNFR-KVRHRHNED------SRFPRNTRNL----HEPRFTHSEY--HRTVDEN-VQPGTSILTVEATDD
Sm1 N-DGELSHIKRSHQPSL----KYNAIKVHSH--HNVSKRSLE-KASLIQ----ASSRLQANEKVL---RPHMTFEYPDYQTPTPFPED-VAIGTELLKVNVVNR
Sm2 MG-AGTFNIGPSTGIVK-----LAKELDYEDP---RQPHTYSLIVTATED-SGGFSTSVELTIKVTDVNDNPPKFELPDYQAH-NVDED-IPIGTSILKVRATDA
Cm QG-AGTFNIGPTSGIVK-----LAKELDFEDV---RQPHIYQLVVTATED-SGGFSTSVDLTIKVTDVNDNPPKFDLPDYQAH-NIDED-IPIGSSILKVKARDD
Le2 QG-AGTFNIGPTSGIVK-----LAKELDFEDV---TQPHIYQLVVTATED-SGGFSTSVDLTIKVTDVNDNPPKFDLPDYQAH-NIDED-IPIGSSILRVKAKDD
Dp2 QG-AGTFNIGPTTGNVR-----LAKDLDFEDL---RQPHVYQLQVTATED-SGGFSTSVELTIRVTDVNDNVPKFELPDYQAH-NIDED-IPVGTSILKVKATDS
Am2 QGAAGTFNIGPTTGIVK-----LAKELDFEDL---RQPHIYSLIVTATED-SGGFSTSVELTIRVSDVNDNAPKFELPDYQAH-NVDED-ISLGTSILKVKATDA
DN QG-AGTFNIGPTSGIVK-----LAKELDFEDL---RQPHVYSLIVTATED-SGGFSTSVDLTIRVTDVNDNAPKFELPDYQAH-NVDED-IPLGTSILRVKAMDS
Pt1 KG-AGTFNIGPTTGVVK-----LAKDLDYEDL---RQPKSYSLIVTATED-SGGFSTSVELTIKVTDVNDNAPRFELPDYQAH-NVDED-VAVGTSILQVSASDM
Pt2 QG-AGTFIIGPTDGIVK-----LAKDLDYEDL---RQPKKFSLQITATED-SGGFSTNVELNIDVTDVNDNPPRFEVPDYQAY-GVPED-APIGTSILQVSATDT
Ct -GQSNEFAISQTKGVVD-----LLRKLDYEK----DMHQYHLRVKAVE--NGRPTRTSTLIVDLIDMNDNAPMFPLSQYIMH-GIAET-ASIGTDIMQVTATDL
Lg TQASPYFTIDSTTGVVKTTSTLQSSDLDYDISPKSSRPPKYSLTARATESETG-LSSTIDLNVEVIDNNDNSPIFEYSSYQ--RIIPENYRVNS-TILTVKADDD
LvG PN-RPSHTINADSGVIT-----LTEAVLVED------MQSYFLIVRATEQGGNQLSSDVRVNVMIEDINNCVPTFGQEILSF-NDVSETTA-IGEPVGEVTATDC
Bf SMD--MFAINRLNGQIT-----LHRGIDYETE-----QRQYTLSVEATETGVNPMSSIVQVRVTVADINDCTPEFANPIYSQ-RDVVEN-IPSTKAILQVSASDC
Pn ----NLFSINQESGALS-----LTRNVDYES----GHNLHTLQVRASEPDTG-LSSVAEVVVHITDENDCTPEFLHSIYT--RDNIPESIAPGTSLLQVLARDC
Mm5

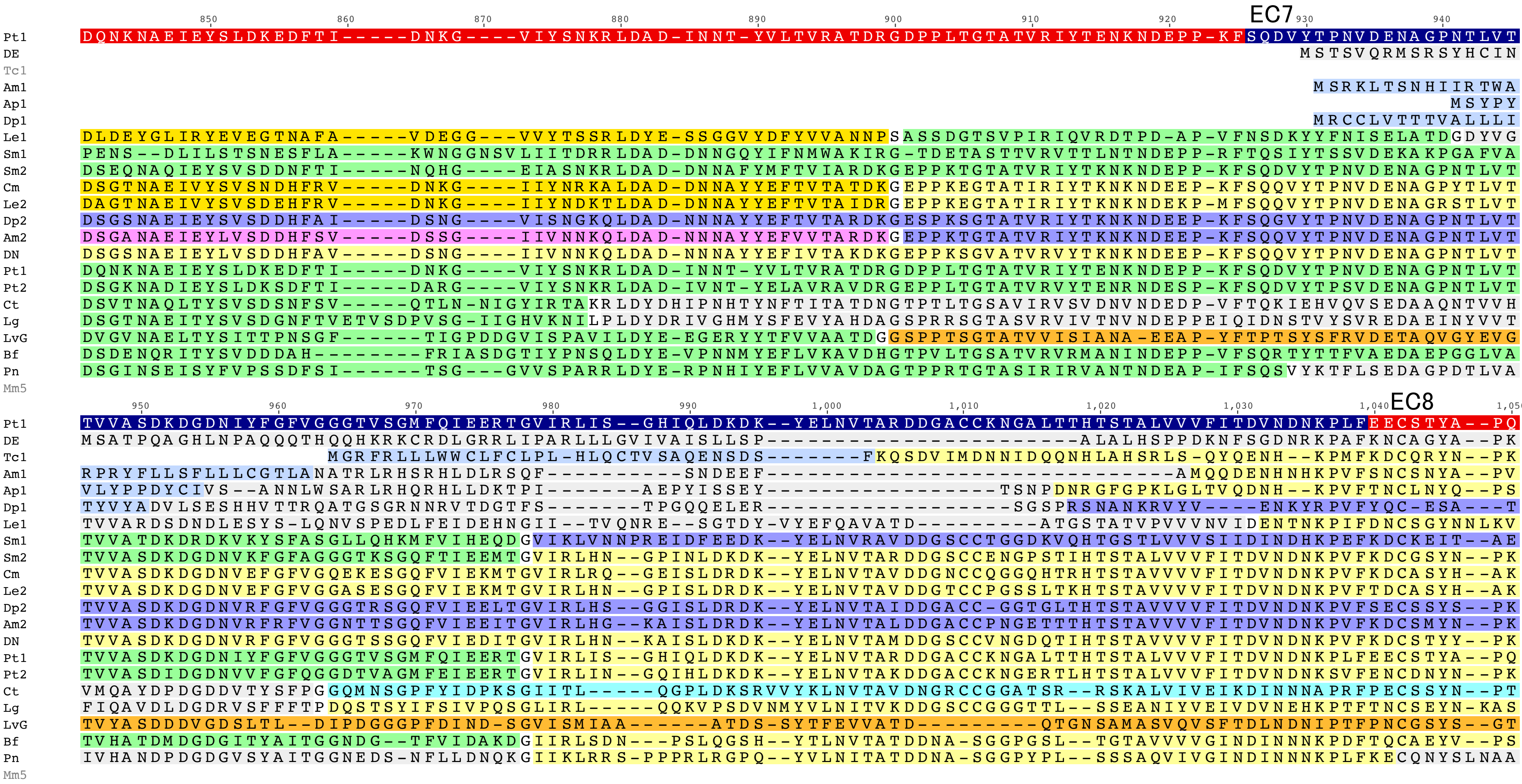

EC7
EC8
Pt1
DE
Tc1
Am1
Ap1
Dp1
Le1
Sm1
Sm2
Cm
Le2
Dp2
Am2
DN
Pt1
Pt2
Ct
Lg
LvG
Bf
Pn
Mm5

```
          1,060     1,070     1,080     1,090     1,100     1,110     1,120     1,130     1,140     1,150
Pt1  VEEGAPSGTQVIKVK-AIDNDKGHN-GQ-VRYSIVQQP-NQKGTKFIVDEVTGDIRTNKVFDREGDD-GRFVSVTVKATDRGNPPLEGVCSFKVEITDINDNPPL
DE   VKEEQPENTYVLTVE-AVDPDPDQ----VIRYSIVQSP-FER-PKFFINPSTGVIFTTHTFDRDEPIHEKFVFVTVQATDNGLPPLDDVCTFNVTIEDINDNAPA
Tc1  VEEEQPSQTFVIRVQ-AVDEDPPDREGGRITYTIEEPP-GTR-PNFKINKRTGEITTALPFDRDEPNRQKEIYLTVRAVDDGRPELADICTIKVTVEDINDNSPS
Am1  VKEEEPVGTVVIQVH-AKDRDNPDN-GGTITYSFVTAP-GEK-LKFEINNRTGLIRTTQVLDRDEPAREKEAYLTVLATDNGRPQLDDVCTFKVTIEDVNDNSPV
Ap1  VKEEQPTNTYVFTVK-AFDKDPPDN-GGTITYTFVSAP-GER-PKFSIDAQTGEITTRHMFDRDEPTREKEVYLSVRATDNGKPHLDDVCTIKVTIQDENDNSPV
Dp1  LEEGKQPGTVVSTVR-AYDNDTDSA-GE-IFFTILRAG-SEKE--FEVDPVTGTIRSSKSFDRDEPAREKIAYVTVKATDRGTPPLEAICTFNVTIGDINDNAPM
Le1  KENQTVPQDILI-IT-ATDADHGKN-GY-VEYSLLTDD-PE----FAIDPVSGQLTVQQPLDRDNGL--KEHFLTVIAKDMADDPLQSSCSFTIIVTDINDNPPV
Sm1  VDENMPIGTFVIKMT-ATDDDRGMN-KV-LNYSIVMQP-DQTESNFIIDVKDGSVNTNKVFDREAEN-GKVVSVTVKATDNGNPPLEGLCTFMVNIKDVNDNAPQ
Sm2  VQEGAPSGTHVLKVT-ATDEDKGQN-GQ-VQYSIVQQP-NQKGTKFIVDQATGDIKTNKVFDREGED-GKFVSVTVKATDLGNPPLEGVCSFKVEITDINDNPPL
Cm   VEESAPNGSPVITVH-ATDEDKGVN-GQ-VRYSIVQQP-NQKGTKFTVDPETGEVTTNKVFDREGED-GKFVSVTVKATDNGDPSLEGVCSFTVEITDINDNPPL
Le2  VEESAPNGSPVITVH-ATDEDKGGN-GQ-VRYSIVQQP-NQKGTKFTVNPETGEVTTNKVFDREGED-GKFVSVTVKATDNGDPSLEGVCSFTVEITDINDNPPL
Dp2  IEEGAPNGSPVVRVQ-ATDADKGVN-GQ-VKYSIVQQP-NQKGTKFTVDEETGEIFTNKVFDREGDD-GKFVSVTVKATDQGDPSLEGVCSFTVEITDVNDNPPL
Am2  VEEGAPNGSTVIKVQ-ATDEDKGVN-GQ-VKYSIVQQP-NQKGTKFTVDEETGQVLTNKVFDREGDD-GKFVSVTVKATDQGEPSLEGVCSFTVEITDVNDNPPL
DN   VEEGAPNGSPVIKVV-ATDEDKGVN-GQ-VKYSIVQQP-NQKGTKFTVDEETGEVSTNKVFDREGDD-GKFVSVTVKATDQGDPSLEGVCSFTVEITDVNDNPPL
Pt1  VEEGAPSGTQVIKVK-AIDNDKGHN-GQ-VRYSIVQQP-NQKGTKFIVDEVTGDIRTNKVFDREGDD-GRFVSVTVKATDRGNPPLEGVCSFKVEITDINDNPPL
Pt2  VEENADPGTFVIRVE-ATDQDKGYN-GQ-VRYSVVQQP-NQRGTKFIVDEITGDVRTNKVFNREGED-GRFVSVTLKATDRGNPPLEGVCSFKVEITDVNDNPPL
Ct   VLEKEDVGTSVVQVR-AVDQDSGAN-GN-ITYSIVKSN-DQSSERFQIEPTSGILRTADIFDREGQIGVTGFGVTIKAKDQGNPSLAGFCTVRVKIGDTNDNPPV
Lg   LLENSPSNTPIIKVH-AVDEDRGEN-GR-VTYSILRSSTDTT-RKFNIDPNTGNITSLIQFDREK---THSHSITIKGNDQANPSLEGYCTFSINIGDVNDNSPV
LvG  VAEDAAIDTSIITVS-ASDLDQGSN-AE-VTYQL-ASNTDA-NQVFKVDQD-GLITTKKLLDREDTS---SFVVTVEAEDGGNPSLTGFCTFSVVVTDVDDNYPE
Bf   VPEGQPSGTFVIQVT-ASDADQGSN-GR-VTYSIVHRE-DS-ETMFNIDADSGEISTAVVFDREERR---EHAVTVTATDNADNPLIGVCQLIVHITDVNDNDPV
Pn   VLENQPPGTFVLRVQ-AHDADMGVN-GE-VKYGIMNRD--GVASGFDIDPDTGVITTAVSFDRERQR---EYTLSVTATDQAEEPLIGICQITVQISDQNDNDPK
Mm5

     EC9 1,160     1,170     1,180     1,190     1,200     1,210     1,220     1,230     1,240     1,250     1,260
Pt1  FDRQEYRENVKQDT-PKGTNILRVSASDEDADNNGAIVYNLTAPYD-PTDLDYFEINP-DSGWISLRKALD-----RDQYHLRAIALDKGLPQHRATVEVIIDVV
DE   FNKARYDESMSENA-QPDAVVMTISASDFDDGNNSLVEYEILRE----RDFQYFKIDK-ESGIIYLKRPIDKRP-GQSYAIIVRYANVVPDPPQDAQIEVRIRVV
Tc1  FDQHDYSSNVSEDL-PENSEVLRVFAYDLDDRENARLTYSFVNGN--DQFEDYFRIDS-VTGVIYLKKQLLGKQ--SKRFSSTVEVSDNGKEKNTARADVVFTVV
Am1  FDKVAYTESVPQDL-PLSREVMRVSATDIDDGNNSVVYYSLSPKK--PEDGVYFRIDR-NTGVIFLNKTIDKVP--DYKFSMTATAQDQGENPKSNVIDLDIRVV
Ap1  FDKVNYVESVPQDL-LSKSEVMRISATDIDDGINSAIQYDLSPRM--PQDSNYFRIDQ-NTGVIYLDRAIDRDP--GYKFKMRATATDQGLVPKSATMDLEILVV
Dp1  FDKSAYDESVPRDL-ELGKLVMRIAATDVDAGENAHISYGWDAGSG--PDAEYFDLDK-STGAIHLKKKITQNE--GYKFALMAVARDNGTEPMNSRVRVMINVA
Le1  FDLESYSQTVPSDT-EENSPILRLTASDDDAGANAEIVYSLEGSDE---DLEYFSVDR-DLGYIILQDPLTQSMANEREFIFKARATDVGEPD-QEPLTSTVDVV
Sm1  FNRDSYQQTITPNQKPREDTLFWIAATDRDADRNAMIGYTLERKHN-NNDLDYFAINP-DSGRVTLKKHLSS----QTNFNFYAVATDKGEPPLSSKVDVRVDVL
Sm2  FDRQEYRERVKQDT-SIGTNILRVSASDEDADNNGAIVYNLSFPYD-IKDMEYFDINP-DSGWIILKKPLD-----KEGYAFQAIAQDKGVPPLSKAVDVTIEVV
Cm   FDRQKYVENVKQDT-NVGTNILRVSASDEDADNNGAILYNLTAAAT-PADLEYFEIQP-ESGWITLKKPLD-----RGDYEVVVQATDQGKVPLTSEVLVQLSIV
Le2  FDRQKYIENVKQDT-NVGTNILRVSASDEDADNNGAVVYNLTASTN-PGDLEYFDIQP-ESGWITLRRPLD-----RELYVVRVVSTDKGSPPLNSAVLVQLNVV
Dp2  FDRQKYVENVKQDT-NIGTNILRVSASDEDADNNGAIVYNLTA-YN-SQDLEYFTILP-ESGWIVLIKPLD-----RETYKLEAVAQDRGFPPLSRTVEVQIDVV
Am2  FDRQRYVENVKQDA-SIGTNILRVSASDEDADNNGAITYSLSSPNN-DQGLEYFEIQP-ESGWIVLKKPLDGCFP-RDSYRLRVRASDRGMQVLHADVDVELDVV
DN   FDRQKYVENVKQDA-SIGTNILRVSASDEDADNNGAIVYSLTAPFN-PNDLEYFEIQA-ESGWIVLKKPLD-----RETYKLEAMAQDKGYPPLSRTVEVQIDVV
Pt1  FDRQEYRENVKQDT-PKGTNILRVSASDEDADNNGAIVYNLTAPYD-PTDLDYFEINP-DSGWISLRKALD-----RDQYHLRAIALDKGLPQHRATVEVIIDVV
Pt2  FHRSEYHENIKQDT-PVGSNVLRIAASDEDADNNGVVVYSLF-PTS-SEYRGYFSINP-ESGWISLKKSLD-----REKYFLRAEATDKGVPPMKASVNVILEVV
Ct   FNLPEYSTSIEENS-VVGKRVKQVYATDRDAGDNGKVGYFME------SDPSGFFAIHRSSGWVTVARPMAG----REHVTIIIRAQDKGTPSKSTTVSMDIQIT
Lg   FDQPSYTVNIVQDY-EVGRSVANVRAEDADLGINAEIEYSLISN---PNDM--FRIDI-DDGIIYLDKDLGT----DTAYQLQVMAKDKGIIPLNANVSVTVKVA
LvG  FPFTDYKASVLSDA-PSNSEVVSVPA--DDLDSSSTLQYDLASSQEECRR--YFTMSP-ADPVIITRGDLSAI-------SNNDVSCIVTASDGIRESRVDVDIF
Bf   FENNKYAAVLSENT-ALHTSFLRVAA--QDAD-MGTNAEILYSLSEEV--PQYLGID-RQTGWLYVKSAISQTQ------RIIRRVIATDGGRSTSVRLDVAIT
Pn   FENSRYQYFLREDT-PVGTSFLRAAAHDDD---QGSNAAITYSLS--RQKPAYLHINP-STGWIYVNHPISQTS------RINQQIVASDGGNRSSSVELTVIIT
Mm5
```

```
     1,270 EC10  1,280     1,290     1,300     1,310     1,320     1,330     1,340     1,350     1,360
Pt1  DRANNPPIWDQPVYG--PIFI--KENYMVGQ-VVASIKARSGIPDNPTVFYTLMKGSTEQTNKKDTFYLDQ-------RNLNGDTWADIQVNYPLDYERIQQYNL
DE   ESSIKPPSFVNPID--TPIYL--KENLKNFTHPIATLRAVSNMPDKPEVIFELNTGRTEQTNSKNTFVFN----------QIG-NEVTISLGKTLDYEAITDYTL
Tc1  DSNKRPPTIEVVGGN-RVIHL--KEDENDFEKPLVALSAQSNIDDPS-VVFELVKGKTTQTNKDQTFILS----------PSGPTSAQITMARPLDYETVEEYQL
Am1  ESHKKAPVFLPR-PA-EPIRL--QENFSDFDASIVQLKAVLNSGDK--LLFELVPGTTEQTNKGNTFRLE----------SNK-DVADIKLSQHLDYESNTQYTL
Ap1  ESHKKAPTFISVQPD-RIISL--PENLTNFSFDIATIIAASNTDEKA-PVFELVPGRTEQSNKLNTFRLE----------SEG-NTAHIRLSRHMDYESISEYIL
Dp1  SSKIKPPYFSDI-PE--PVSL--PENYTSYKTAIATLGAHSNIAAPD-LYFELVKGQTEQTNSDETFRIN----------ELGN-SVNIHVAKALDYEKVTQYIL
Le1  LSVVSPGSMPPTVENQNPELAQVNEDATLNT-NIVSICASSNSEDNS-VYMDMINGNVPETNSEGTFDIRTGTSEETGCPSNTIGGNIFLATQNLDFEEIQSYTL
Sm1  RENALVPKWVTNTKE--PISI--SESTPIGT-LVATLEARSESPDNEKVTYRLLKGNSEQSNKNDVFLLSP-------STKDGKDVALITLNKVLDYETIAEYNL
Sm2  DRANNPPVWDQTTYG--PVYI--KENTTVGT-RIVSLKARSGIDDNPVVFYRIIRGSTAQTNKFDTFYVQQ-------RSENGWTWADIFVNQQLDYESIKEYNL
Cm   DRANRPPVWDHQVYD--PITI--PENISVGQ-TIFSMKASSGIEDNPVVFYRIIRGSTAQTNKHDTFYLQQ-------RPDNGDTWADIKVNHPLDYESIKEYNL
Le2  DRANRPPVWDQRVYG--PINI--FENTSVGH-TIFSVKASSGIEDNPVVFYRIIRGSTAQTNKHDTFYLQQ-------RPDNGDTWADIKVNHPLDYESIKEYNL
Dp2  DRANNPPVWDQVVYG--PIFV--KENKNVGD-KVVSVKASSGIEGNPTVFYRLIRGSTAQTNKYDTFYLQQ-------RADNGHTWADIKVNHPLDYENIKEYNL
Am2  DRNNKPPVWDANVYG--PIHI--KENVTVGT-VVTSVKASSGIENNPTVFYRLMPGSTAQTNKFHTFYLQQ-------RAEKSVTWADIKVNHQLDYESIKEYNL
DN   DRANNPPVWDHTVYG--PIYV--KENMPVGG-KVVSIKASSGIEGNPTVFYRLMPGSTAQTNKFHTFYLQQ-------RPDNGDTWADIKVNHPLDYESIKEYNL
Pt1  DRANNPPIWDQPVYG--PIFI--KENYMVGQ-VVASIKARSGIPDNPTVFYTLMKGSTEQTNKKDTFYLDQ-------RNLNGDTWADIQVNYPLDYERIQQYNL
Pt2  DRKNNPPVWDQPVYG--PISL--KENIQPGQ-KVAALKARSGIPDNPRVFYTLIKGGTEQTNGKDTFYLSQ-------YSEGGSTWAGIYVNYVLDYEKVQQYNL
Ct   QTVNAYPQWVE-DYSSTPLSL--SENAPVNT-VVKTVKATSSIPDSYVN-YVIQPGEAPEQNSLRSFYY---------RIDEDTNEMVLLTYKPLDFEALSSYTI
Lg   NITSKPPSWDRNDYDSTPYKV--NETVPFGY-MIAEFAASSNVDGNPRVLFNLINGDGNAVAVLDPFYL-----------EIQGNKVQLKVNSRLDYNVKPLHEL
LvG  VRSPDDTTYQVPTFSNTPYQVTINENTTIGD-VILLVS----VDGAQEVGFDIVRGSRPSTNSDGTFTL---------TTRDDNLQSDLTLVEKLDFEAVQEYEL
Bf   DVENEPPMWE-REY---PA-VTIPENTPIDQ-VVATVKASSTLLDSRIT-YSLVDGQLPETNKPKRFYL---------RTNRATRTADICVFHPLDFETTPRFEL
Pn   NVHNQPPQWEQSEYW-----VTVPENTVRDA-KIVTIKATSPLGDPRVT-YNLEEGQVPETNMPVRFYM---------KPNRADGSASILVAENLDYETTRFFTL
Mm5

     1,370     1,380     1,390     1,400 EC11  1,410     1,420     1,430     1,440     1,450     1,460     1,470
Pt1  TVRVENNGI-QQLASEATVYIVLEDVNDEIPLFIERE------QETVLEGMPPGTKVTQVQ--ASDKDGTYPNNKVYYAI-ES--K-DQGDKFFTIDRETGEIYT
DE   TMIVRN---THELGTEHQIKIQVEDVNDNIPYYTEVK------SGTILENEPPGTPVMQVR--AFDMDGTSANNIVSFEL-ADNR------EYFTIDPNTGNITA
Tc1  TVRAKN---KDLLYASVTINVKVEDVNDEIPTFIELL------RGSIVENDKIGAQAMTVR--AIDKDGTSPNNLVVYEL-LDYK------DLFEIDKNSGVITA
Am1  IVRVQN---KYQLAAETTVVIEVLDVNDNIPVFREMK------KGSVLENEPPGVPVMQVR--AIDADGTSAHNQVTYEL-DNFR------DLFAIDKYTGNITT
Ap1  NIRIQN---KHNLAAEHQLNIQVEDVNDNIPVFTEVV------SGSVLENEPAGTAVMQVR--AIDADSSMANNRVTYEL-ADNR------DYFAIDPYTGNITT
Dp1  TVRVKN---EAGVAAETVLTVNVEDVNDEIPTFMAVD------GGSVLENSPANTPVLTVQ--ATDRDATYPNNWVRYEL-RDFK------DKFEIDRNTGDIKT
Le1  IIQLAN----NNGRAEYSLTVDVIDVNDNPPVLQQPY-------TGSIGENVDSTMVMVVQ--AVDKDITPEFRQLEYSF-KDPPD--HVTDNFEIDPVSGEIWN
Sm1  AVEVLNV---FQLASEATIYIRLTDVNDEVPLFNEIE------SAIVLENEPPGTRVVNII--AHDKDATPLFRKVTYTL-TS------GYENFKINPDTGEVTT
Sm2  TIRVENNGA-QQLASEATIYIILEDVNDEIPLFTESE------QGSVLEGEKVGSLVTQIN--AIDKDGTYPYNQVFYSI-VDTPN-NEGSKFFDIDRSSGEVFT
Cm   TVRVENNGP-QQLASEATIYVVLEDVNDEIPLFTERE------QETVLEGEPIGTKVTQVN--AIDKDGTYPNNEVLYRI-VDSPR-NEGRAFYEIRERTGEIFT
Le2  TVRVENNGP-QQLASEATIYVVLEDVNDEIPLFTERE------QETVLEGEAIGTKVTQVN--AIDKDGTYPNNEVLYRI-VDSVR-NEGRDHFEIRERTGEIFT
Dp2  TIRVENNGV-QQLASEATVYIVLEDVNDDIPLFTERE------QETVLEGEPIGTGVTRVN--AIDKDGTFPNNQVYYGI-VDSPR-NEGKDYFEINEQTGEIFT
Am2  TIRVENNGA-QQLASEATVYIVLEDVNDEIPLFTERE------QETVLEGEPVGSKVTQVN--AIDKDGTFPNNQVTYYV-VDSDR-NEGKDYFEINRETGEIFT
DN   TIRVENNGA-QQLASEATVYIMLEDVNDEIPLFTERE------QETVLEGEPIGTKVTQVN--AIDKDGTFPNNQVYYYI-VDSPR-NEGKEFFEINLQSGEIFT
Pt1  TVRVENNGI-QQLASEATVYIVLEDVNDEIPLFIERE------QETVLEGMPPGTKVTQVQ--ASDKDGTYPNNKVYYAI-ES--K-DQGDKFFTIDRETGEIYT
Pt2  TVRVENNGA-QQLASEATVYIVLQDVNDEIPLFVEQE------QETVLEGMPIGTKVTQIR--AVDKDGTYPNNKVYYSI-ED--K-DDGEK-FRIDRETGEIFT
Ct   TIKAANRAT-NSLHNDTRLVINLVDENDEIPQFIGLD-ENGRYAGSVAENLNPGTEVITVT--AMDRDEFPDYNKKMARXS--------------------------
Lg   RLRVTNYNF-IPSTSEISPKIEVIDMNNKAPYFVGRNQDSSIYPGSVAENMPPGQTVIDVK--AYDDDVFPPNNELSFSL-VYKEGVSQLFSISKIDDNTARITT
LvG  LVTAQYVGVGQEFIAQTPVTVTIEDFNDVAPKFPAVT-----FYGNIAENQPVSNSDPIITLQATDGDVTPEYSQISYSF-------VNAESDFSIDEDTGEIFA
Bf   KIRAENAAR-IPLAAYTIVEIRLTDVNDEVPDFTAKS-----YAMSVAEQSPKGTLVGQVT--AVDAD-TGDAGKVTYSISPDPDPSLRDWEKFRIDPSTGEVFT
Pn   RVRVQNVAA-VPLASFTTVYVNVTDVNDNVPFFLSST-----YEATVPEGAQMGTSVAQVS--ATDLD-SGLHGMIHYVILKDES--GDSQFFSIDSHSGIIFT
Mm5
```

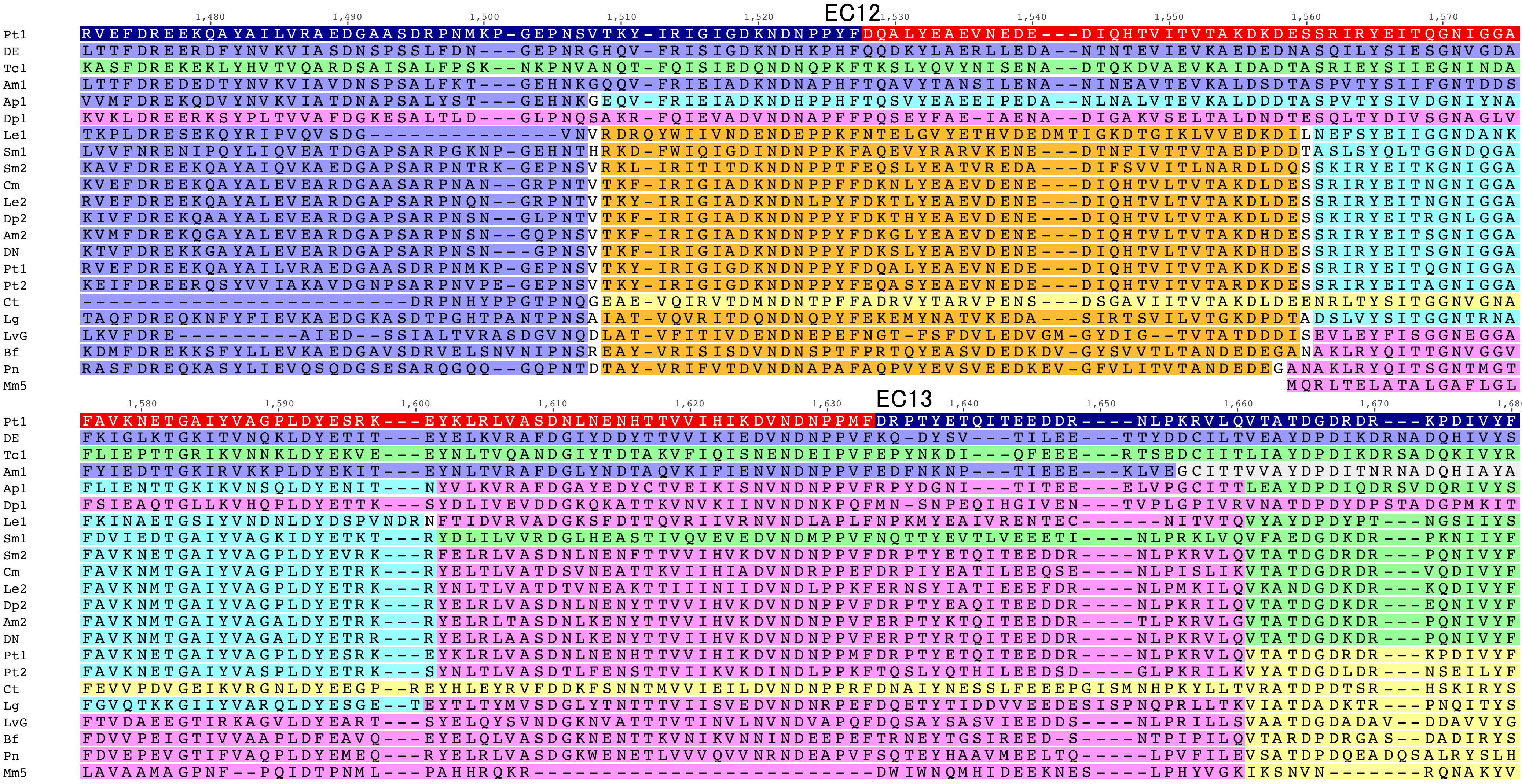
EC12
EC13
Pt1
DE
Tc1
Am1
Ap1
Dp1
Le1
Sm1
Sm2
Cm
Le2
Dp2
Am2
DN
Pt1
Pt2
Ct
Lg
LvG
Bf
Pn
Mm5

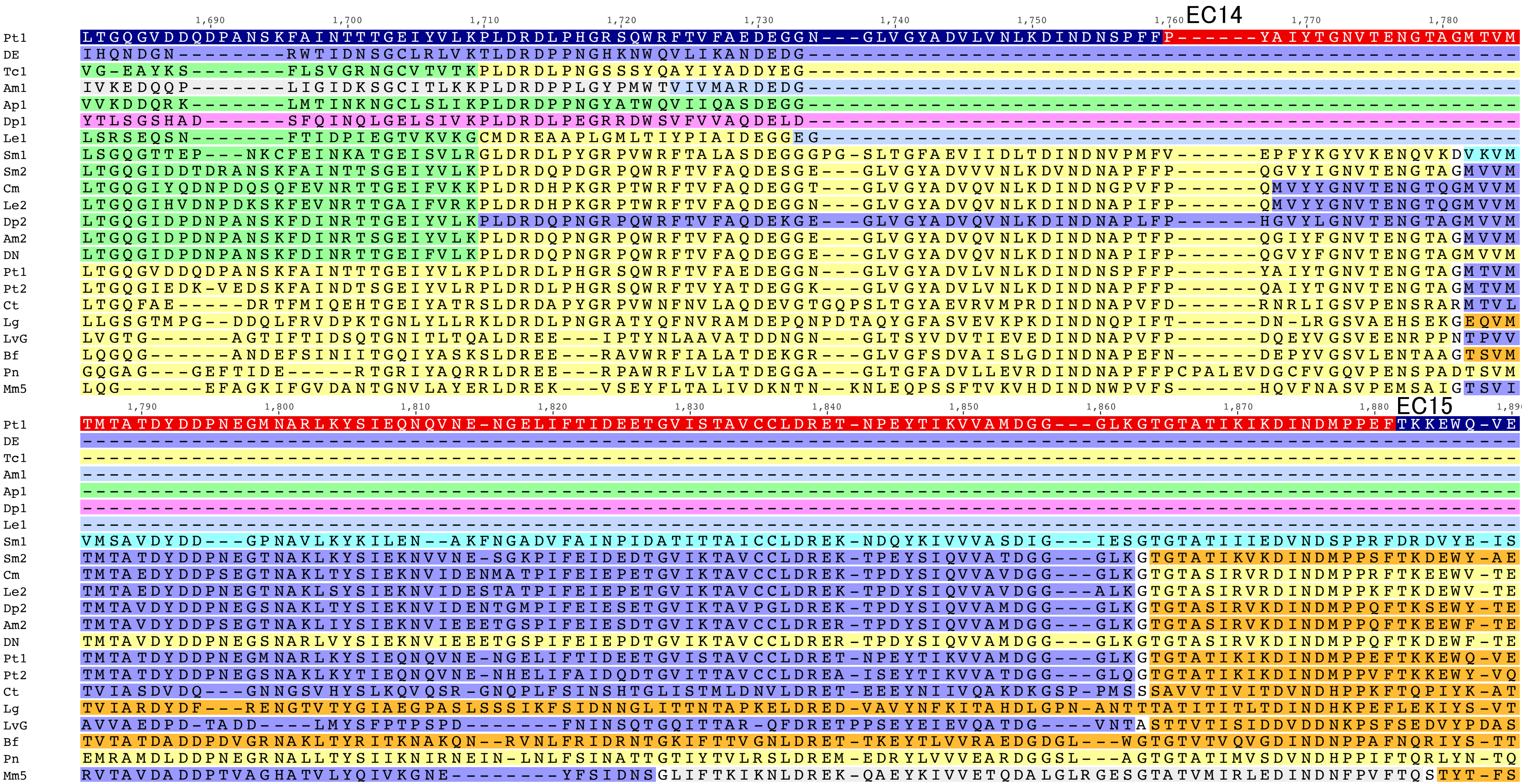

1,690 1,700 1,710 1,720 1,730 1,740 1,750 1,760 EC14 1,770 1,780
Pt1 LTGQGVDDQDPANSKFAINTTTGEIYVLKPLDRDLPHGRSQWRFTVFAEDEGGN---GLVGYADVLVNLKDINDNSPFFP------YAIYTGNVTENGTAGMTVM
DE IHQNDGN--------RWTIDNSGCLRLVKTLDRDPPNGHKNWQVLIKANDEDG--
Tc1 VG-EAYKS-------FLSVGRNGCVTVTKPLDRDLPNGSSSYQAYIYADDYEG--
Am1 IVKEDQQP-------LIGIDKSGCITLKKPLDRDPPLGYPMWTVIVMARDEDG--
Ap1 VVKDDQRK-------LMTINKNGCLSLIKPLDRDPPNGYATWQVIIQASDEGG--
Dp1 YTLSGSHAD------SFQINQLGELSIVKPLDRDLPEGRRDWSVFVVAQDELD--
Le1 LSRSEQSN-------FTIDPIEGTVKVKGCMDREAAPLGMLTIYPIAIDEGGEG---
Sm1 LSGQGTTEP---NKCFEINKATGEISVLRGLDRDLPYGRPVWRFTALASDEGGGPG-SLTGFAEVIIDLTDINDNVPMFV------EPFYKGYVKENQVKDVKVM
Sm2 LTGQGIDDTDRANSKFAINTTSGEIYVLKPLDRDQPDGRPQWRFTVFAQDESGE---GLVGYADVVVNLKDVNDNAPFFP------QGVYIGNVTENGTAGMVVM
Cm LTGQGIYQDNPDQSQFEVNRTTGEIFVKKPLDRDHPKGRPTWRFTVFAQDEGGT---GLVGYADVQVNLKDINDNGPVFP------QMVYYGNVTENGTQGMVVM
Le2 LTGQGIHVDNPDKSKFEVNRTTGAIFVRKPLDRDHPKGRPTWRFTVFAQDEGGN---GLVGYADVQVNLKDINDNAPIFP------QMVYYGNVTENGTQGMVVM
Dp2 LTGQGIDPDNPANSKFDINRTTGEIYVLKPLDRDQPNGRPQWRFTVFAQDEKGE---GLVGYADVQVNLKDINDNAPLFP------HGVYLGNVTENGTAGMVVM
Am2 LTGQGIDPDNPANSKFDINRTSGEIYVLKPLDRDQPNGRPQWRFTVFAQDEGGE---GLVGYADVQVNLKDINDNAPTFP------QGIYFGNVTENGTAGMVVM
DN LTGQGIDPDNPANSKFDINRTTGEIFVLKPLDRDQPNGRPQWRFTVFAQDEGGE---GLVGYADVQVNLKDINDNAPIFP------QGVYFGNVTENGTAGMVVM
Pt1 LTGQGVDDQDPANSKFAINTTTGEIYVLKPLDRDLPHGRSQWRFTVFAEDEGGN---GLVGYADVLVNLKDINDNSPFFP------YAIYTGNVTENGTAGMTVM
Pt2 LTGQGIEDK-VEDSKFAINDTSGEIYVLRPLDRDLPHGRSQWRFTVYATDEGGK---GLVGYADVLVNLKDINDNAPFFP------QAIYTGNVTENGTAGMTVM
Ct LTGQFAE-----DRTFMIQEHTGEIYATRSLDRDAPYGRPVWNFNVLAQDEVGTGQPSLTGYAEVRVMPRDINDNAPVFD------RNRLIGSVPENSRARMTVL
Lg LLGSGTMPG--DDQLFRVDPKTGNLYLLRKLDRDLPNGRATYQFNVRAMDEPQNPDTAQYGFASVEVKPKDINDNQPIFT------DN-LRGSVAEHSEKGEQVM
LvG LVGTG------AGTIFTIDSQTGNITLTQALDREE---IPTYNLAAVATDDNGN---GLTSYVDVTIEVEDINDNAPVFP------DQEYVGSVEENRPPNTPVV
Bf LQGQG------ANDEFSINIITGQIYASKSLDREE---RAVWRFIALATDEKGR---GLVGFSDVAISLGDINDNAPEFN------DEPYVGSVLENTAAGTSVM
Pn GQGAG---GEFTIDE-----RTGRIYAQRRLDREE---RPAWRFLVLATDEGGA---GLTGFADVLLEVRDINDNAPFFPCPALEVDGCFVGQVPENSPADTSVM
Mm5 LQG------EFAGKIFGVDANTGNVLAYERLDREK---VSEYFLTALIVDKNTN--KNLEQPSSFTVKVHDINDNWPVFS------HQVFNASVPEMSAIGTSVI
1,790 1,800 1,810 1,820 1,830 1,840 1,850 1,860 1,870 1,880 EC15 1,890
Pt1 TMTATDYDDPNEGMNARLKYSIEQNQVNE-NGELIFTIDEETGVISTAVCCLDRET-NPEYTIKVVAMDGG---GLKGTGTATIKIKDINDMPPEFTKKEWQ-VE
DE ---
Tc1 ---
Am1 ---
Ap1 ---
Dp1 ---
Le1 ---
Sm1 VMSAVDYDD---GPNAVLKYKILEN--AKFNGADVFAINPIDATITTAICCLDREK-NDQYKIVVVASDIG---IESGTGTATIIIEDVNDSPPRFDRDVYE-IS
Sm2 TMTATDYDDPNEGTNAKLKYSIEKNVVNE-SGKPIFEIDEDTGVIKTAVCCLDREK-TPEYSIQVVATDGG---GLKGTGTATIKVKDINDMPPSFTKDEWY-AE
Cm TMTAEDYDDPSEGTNAKLTYSIEKNVIDENMATPIFEIEPETGVIKTAVCCLDREK-TPDYSIQVVAVDGG---GLKGTGTASIRVRDINDMPPRFTKEEWV-TE
Le2 TMTAEDYDDPNEGTNAKLSYSIEKNVIDESTATPIFEIEPETGVIKTAVCCLDREK-TPDYSIQVVAVDGG---ALKGTGTASIRVRDINDMPPKFTKDEWV-TE
Dp2 TMTAVDYDDPNEGSNAKLTYSIEKNVIDENTGMPIFEIESETGVIKTAVPGLDREK-TPDYSIQVVAMDGG---GLKGTGTASIRVKDINDMPPQFTKSEWY-TE
Am2 TMTAVDYDDPSEGTNAKLIYSIEKNVIEEETGSPIFEIESDTGVIKTAVCCLDRER-TPDYSIQVVAMDGG---GLKGTGTASIRVKDINDMPPQFTKEEWF-TE
DN TMTAVDYDDPNEGSNARLVYSIEKNVIEEETGSPIFEIEPDTGVIKTAVCCLDRER-TPDYSIQVVAMDGG---GLKGTGTASIRVKDINDMPPQFTKDEWF-TE
Pt1 TMTATDYDDPNEGMNARLKYSIEQNQVNE-NGELIFTIDEETGVISTAVCCLDRET-NPEYTIKVVAMDGG---GLKGTGTATIKIKDINDMPPEFTKKEWQ-VE
Pt2 TMTATDYDDPNEGSNAKLKYTIEQNQVNE-NHELIFAIDQDTGVITTAVCCLDREA-ISEYTIKVVATDGG---GLQGTGTATIKIKDINDMPPVFTKKEWY-VQ
Ct TVIASDVDQ---GNNGSVHYSLKQVQSR-GNQPLFSINSHTGLISTMLDNVLDRET-EEEYNIIVQAKDKGSP-PMSSSAVVTIVITDVNDHPPKFTQPIYK-AT
Lg TVIARDYDF---RENGTVTYGIAEGPASLSSSIKFSIDNNGLITTNTAPKELDRED-VAVYNFKITAHDLGPN-ANTTTATITITLTDINDHKPEFLEKIYS-VT
LvG AVVAEDPD-TADD----LMYSFPTPSPD-------FNINSQTGQITTAR-QFDRETPPSEYEIEVQATDG----VNTASTTVTISIDDVDDNKPSFSEDVYPDAS
Bf TVTATDADDPDVGRNAKLTYRITKNAKQN--RVNLFRIDRNTGKIFTTVGNLDRET-TKEYTLVVRAEDGDGL---WGTGTVTVQVGDINDNPPAFNQRIYS-TT
Pn EMRAMDLDDPNEGRNALLTYSIIKNIRNEIN-LNLFSINATTGTIYTVLRSLDREM-EDRYLVVVEARDGGSL---AGTGTATIMVSDVNDHPPIFTQRLYN-TQ
Mm5 RVTAVDADDPTVAGHATVLYQIVKGNE--------YFSIDNSGLIFTKIKNLDREK-QAEYKIVVETQDALGLRGESGTATVMIRLEDINDNFPVFTQSTYT-FS

```
      1,900 1,910 1,920 1,930 1,940 1,950 1,960 1,970 1,980 1,990
Pt1   VDETEGDNIP-ETPILVVSVNDGDLLETNRFSYKVIDNA-FGADKFTMVTNSDGTGSLKIAKPLDFEDLQQRFGFNITIQVSDHGGESS-EPYHVDYAKVRVRLR
DE    ---------------------------------------------------------------------------------------------------------
Tc1   ---------------------------------------------------------------------------------------------------------
Am1   ---------------------------------------------------------------------------------------------------------
Ap1   ---------------------------------------------------------------------------------------------------------
Dp1   ---------------------------------------------------------------------------------------------------------
Le1   ---------------------------------------------------------------------------------------------------------
Sm1   IPETNGNIVKFDEPVLSFKVLDDDLPETNKNYFLIEPKSGPGYDRFIIVPRDPDEGELKVKLPLDYENPEHRKGFHFRIIVDDSGGLSN-DTERMATAEVKIQLI
Sm2   VPETEGDNIP-EQPILVVSVKDDDLPETNNFHYKVIESSGFGADKFTMVRNADGTGSLKVAKPLDYEDFRQRYGFNFTIQVNDKGGDSD-DKYHIAYAKVRIKLL
Cm    VDETEGALLP-DQAILTVTVHDED--ETNKFHYKVIESSGFGADKFTMVRNNDGTGSLKIVQPLDYEDPMQRSGFRFKIQVNDKGEDNDAEKYHVAYSWVKVELR
Le2   VDETYGEILP-DQAILTVTVQDDD--ETNKFHYKVIESSGFGADKFTMVRNNDGTGSLKIVQPLDYEDTMQSSGFRFKIQVNDKGEDNDAEKYHVAYSWVKVELR
Dp2   VDETDGIALP-DAPILTVTVHDED--ETNKFHYKVIESSGFGADKFTMVRNNDGTGSLKVVQPLDYEDKLQRNGFRFMIQVNDKGEDNDNDKYHVAYCWVNVKLR
Am2   VDETEGPDLP-EMPILTVTVHDED--ETNKFQYKVIESSGYGADKFTMVRNNDGTGSLKIVQSLDYEDQLQSNGFRFRIQVNDKGEDNDNDKYHVAYSWVVVKLR
DN    VDETDGTALP-EMPILTVTVHDED--ETNKFQYKVIDNSGYGADKFTMVRNNDGTGSLKIVQPLDYEDQLQSNGFRFRIQVNDKGEDNDNDKYHVAYSWVVVKLR
Pt1   VDETEGDNIP-ETPILVVSVNDGDLLETNRFSYKVIDNA-FGADKFTMVTNSDGTGSLKIAKPLDFEDLQQRFGFNITIQVSDHGGESS-EPYHVDYAKVRVRLR
Pt2   VDETEGDDIP-ELPILVVSVKDEDLLETNRFSYKVINNT-FGADKFTMVTNSDGTGSLKIAKPLDYEDPMQRYGFNITIQVSDHGGETT-NAYHIDYSKVFIRLT
Ct    MSE----NYKAGASITSVSATDKD--MGIHARLTYILNTQDRKYFSMTSVEATNTGVLKVFQPVDYDTLINPV-FNLTVTVHD-PDSRNND---TAY--VEVRVT
Lg    MSE----AQEQGE-IALVRAKDKD--IGENARLTY---SLNSAHFAVNVI--TNEGSITVYKPVDYEDPSQRS-FNLSVTVSD-PDPSHTD---TCY--VEVTVE
LvG   VLETE----PIGTTITTVQAIDPDV-DFRDAVEFSINSGDPDELFRIVADAATLQGLIQVNKELDFETL-ATNEFTLTVAVTDSQGPTDSGRPETAI--VKIIVE
Bf    MSEGLGKDG----IVTTVAASDADV-GDNARLFYSIVGGNRNNQFRMDNSDNK--GIVRVNRRVDYEEP-TQRKFNLTLEVRD-LDFTSRG---YCV----INVL
Pn    VTEDLEVNSE----VLVVSATDGDE-GENAVVTFSIVGGDENRKFFVETDKVNRRGVIKLKKKVDFEKP-NERTFNLTLKAED-ADFFSLA---YCL----IQVE
Mm5   VPEDIRVGKPLGF-LTVV---DPDE-PQNRMTKYSIMQGEYRDTFTIETDPKRNEGIIKPTKSLDYEVI-QQYTFYIEATDPTIRYEYLSSTSGKNKAMVTINVL

      2,000 EC16 2,010 2,020 2,030 2,040 2,050 2,060 2,070 2,080 2,090 2,100
Pt1   DINDNKPEFEKPNIEVIVKEDADI--GTSLATFRATDADQGGKSRVSYTIDRSSDKKRQFRIDQ-NGVVRIQRTLDREETPRHQVKILAID---DGTPPRTA---
DE    -----------------------------------------------------------------------------------------------VGTTVST---
Tc1   -----------------------------------------------------------------------------------------------GPNSLSN---
Am1   -----------------------------------------------------------------------------------------------SPTALRE---
Ap1   -----------------------------------------------------------------------------------------------GPKSLQQ---
Dp1   -----------------------------------------------------------------------------------------------GVRLENT---
Le1   ---------------------------------------------------------------------------------------------------KNS--E
Sm1   DINDNAPEFDNPYINVTVSEGETT--GTRIAKFRATDRDQDGNSTVSYEIERATNRRRHFSILS-DGTVIVQRPLDREREDGHIVHILAID---DGYPAKTS---
Sm2   DINDNKPEFERANIEVSVFENAEV--GRSLAKFSATDADQGGKSQVSYAIDRSSDKRRQFSITQ-DGTVKIQRMLDREDTPRHQVKILAID---DGVPSKTS---
Cm    DINDNKPQFVKPNIEVPVFENAEI--GKSLETFKATDPDQGGQSKVSYAIDRASDRRRQFAIDS-SGTVKIQRTLDREENPRHSVKILAID---DGLPAKTA---
Le2   DINDNKPQFIKPNIEVPVFENAEV--GKNLEQFKAEDPDQGGNSKVSYAIDRASDRRRQFTIDD-TGTVKIQRSLDREENPRHSVKILAID---DGTPAKTA---
Dp2   DINDNKPLFEKANIETSVYENAEV--GKSLETFRATDPDQGGKSKVSFAIDRASDRRRQFAINQ-NGTVTIQRHLDREDTPRHQVKILAID---DGVPPKTA---
Am2   DINDNQPQFEKANIETTVPENARI--GNSLETFKATDPDQGGKSKVYYSIDRSSDRKRQFSINE-NGTVSIQRSLDREETPRHQVKILAID---DGIPPKTA---
DN    DINDNKPHFERANVEVSVFEDTKV--GTELEKFKATDPDQGGKSKVSYSIDRSSDRQRQFAINQ-NGSVTIQRSLDREVVPRHQVKILAID---DGSPPKTA---
Pt1   DINDNKPEFEKPNIEVIVKEDADI--GTSLATFRATDADQGGKSRVSYTIDRSSDKKRQFRIDQ-NGVVRIQRTLDREETPRHQVKILAID---DGTPPRTA---
Pt2   DINDNPPEFSEPLLRASVEENVTV--GYVIKKFQARDPDQAGKSMVKYSIDRSSDKKRQFKISQ-LGVVTIQRPLDRETVNRHDIRILATD---DGTPTRTA---
Ct    DYNDNPPQFVPNIKRVTIHENTTK--DTTLYRFTATDRDTGLNKLFKYSIDRLTDPARQFAVDA-NGLVTISAALDRETEAEHRIHILAID---EGDPPMTG---
Lg    DFNDNAPQFDQASITVNVRENIAV--GEKLATFSANDKDSGLNANFTFHIDTTTDRRRQFAIDEVSGEVSVRRGLDRETLPVHEVRILAID---KGDPQQTG---
LvG   NANDLAPVFDQDVYDGAVSEDATVNSQVGTFTFSATDGDEPG-ATLNYIIDPTTDPDGQFAINE-NQQLIVASPLDRETVASYELKVYAVD---NGEPPMSG---
Bf    DYNDNPPVFNPVYYEKEIYENATV--GTLLRILSATDRDTGNNAVFKYSINPSSDPDQQFLIGAQNGILTVNKALDRETLPQHRLTIQATD---IGPPSLVG---
Pn    DSNDHPPVFFPQFYESPAMSEDVPV-GTIVAQVTASDLDSGQNGRFSYSISKESDPYNQFLVDQ-SGWVVVADSLDREKISQHRIMVLATD---AGSPLLTG---
Mm5   DVDE-PPVFQRHFYHFKLPENQKK---PLIGTVVAKDPDKAQ-RSIGYSIRKTSDR-GQFFRITKQGNIYNEKELDRETYAWYNLTVEANELDSRGNP--VGKES
```

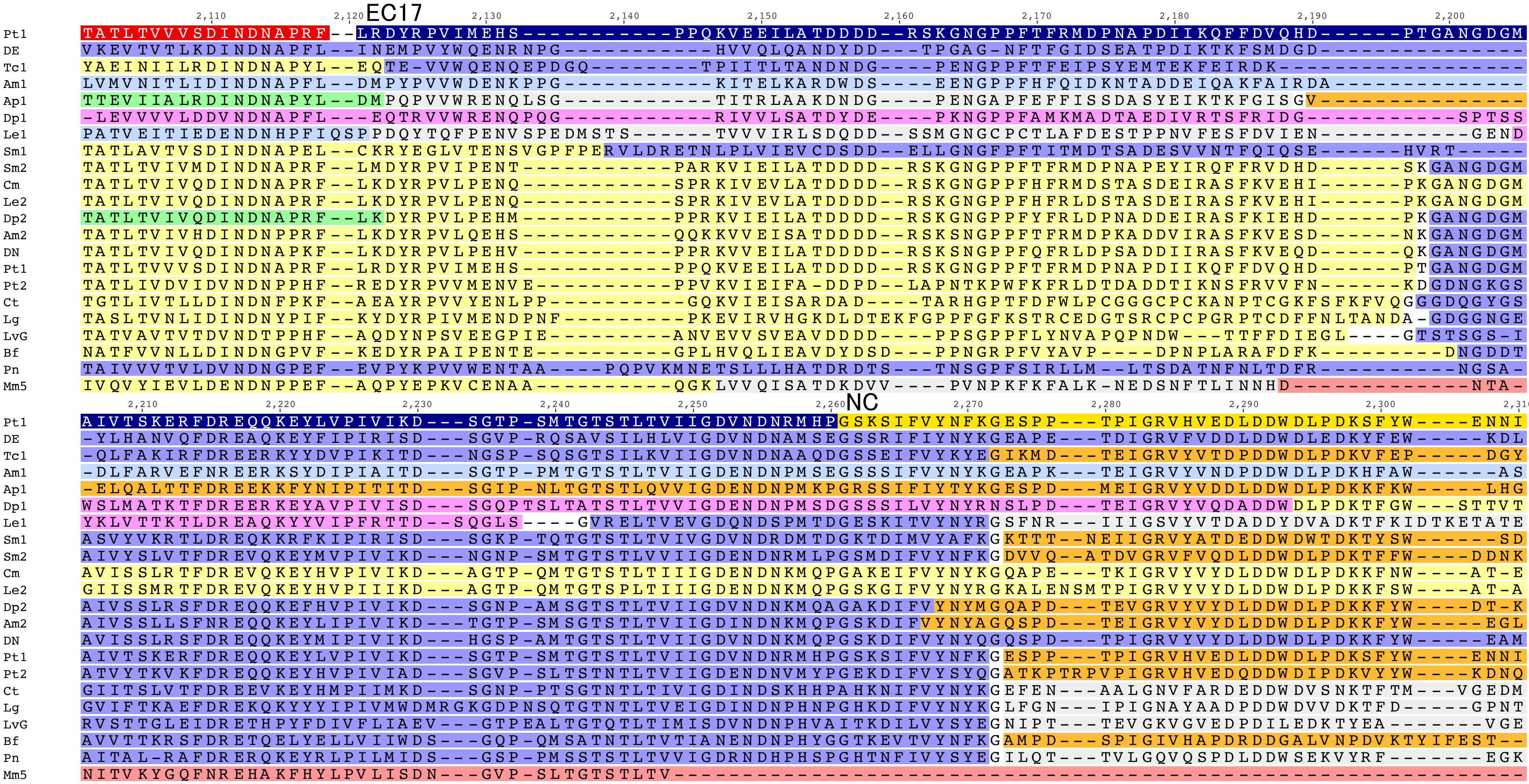
EC17
NC
Pt1
DE
Tc1
Am1
Ap1
Dp1
Le1
Sm1
Sm2
Cm
Le2
Dp2
Am2
DN
Pt1
Pt2
Ct
Lg
LvG
Bf
Pn
Mm5

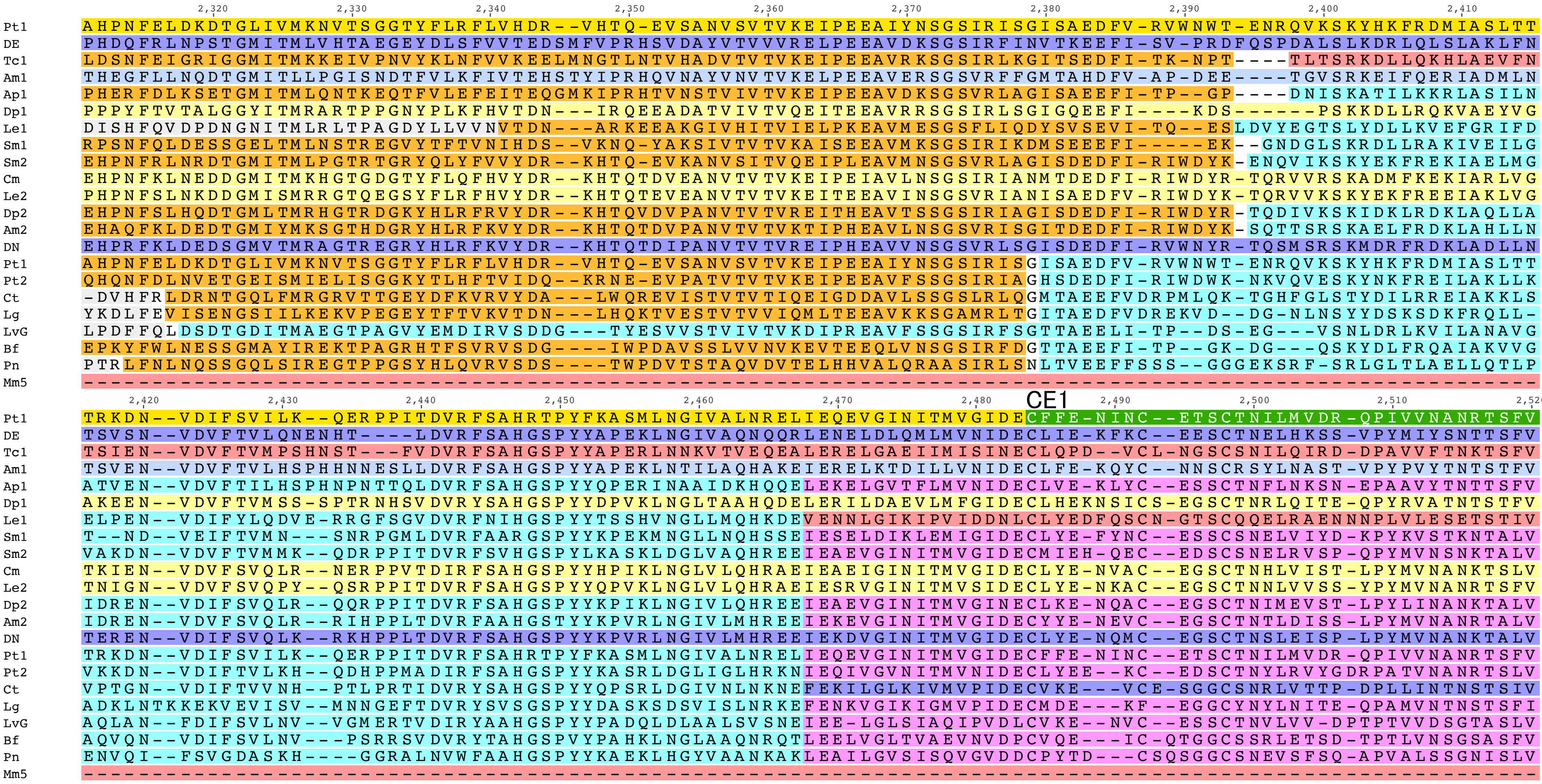
2,320 2,330 2,340 2,350 2,360 2,370 2,380 2,390 2,400 2,410
Pt1 AHPNFELDKDTGLIVMKNVTSGGTYFLRFLVHDR--VHTQ-EVSANVSVTVKEIPEEAIYNSGSIRISGISAEDFV-RVWNWT-ENRQVKSKYHKFRDMIASLTT
DE PHDQFRLNPSTGMITMLVHTAEGEYDLSFVVTEDSMFVPRHSVDAYYTVVVRELPEEAVDKSGSIRFINVTKEEFI-SV-PRDFQSPDALSLKDRLQLSLAKLFN
Tc1 LDSNFEIGRIGGMITMKKEIVPNVYKLNFVVKEELMNGTLNTVHADVTVTVKEIPEEAVRKSGSIRLKGITSEDFI-TK-NPT----TLTSRKDLLQKHLAEVFN
Am1 THEGFLLNQDTGMITLLPGISNDTFVLKFIVTEHSTYIPRHQVNAYYVNVTVKELPEEAVERSGSVRFFGMTAHDFV-AP-DEE----TGVSRKEIFQERIADMLN
Ap1 PHERFDLKSETGMITMLQNTKEQTFVLEFEITEQGMKIPRHTVNSTVIVTVKEIPEEAVDKSGSVRLAGISAEEFI-TP--GP----DNISKATILKKRLASILN
Dp1 PPPYFTVTALGGYITMRARTPPGNYPLKFHVTDN---IRQEEADATVIVTVQEITEEAVRRSGSIRLSGIGQEEFI----KDS------PSKKDLLRQKVAEYVG
Le1 DISHFQVDPDNGNITMLRLTPAGDYLLVVNVTDN---ARKEEAKGIVHITVIELPKEAVMESGSFLIQDYSVSEVI-TQ--ESLDVYEGTSLYDLLKVEFGRIFD
Sm1 RPSNFQLDESSGELTMLNSTREGVYTFTVNIHDS--VKNQ-YAKSIVTVTVKAISEEAVMKSGSIRIKDMSEEEFI-----EK--GNDGLSKRDLLRAKIVEILG
Sm2 EHPNFRLNRDTGMITMLPGTRTGRYQLYFVVYDR--KHTQ-EVKANVSITVQEIPLEAVMNSGSVRLAGISDEDFI-RIWDYK-ENQVIKSKYEKFREKIAELMG
Cm EHPNFKLNEDDGMITMKHGTGDGTYFLQFHVYDR--KHTQTDVEANVTVTVKEIPEIAVLNSGSIRIANMTDEDFI-RIWDYR-TQRVVRSKADMFKEKIARLVG
Le2 PHPNFSLNKDDGMISMRRGTQEGSYFLRFHVYDR--KHTQTEVEANVTVTVKEITEEAVINSGSVRIANISAEDFV-RIWDYK-TQRVVKSKYEKFREEIAKLVG
Dp2 EHPNFSLHQDTGMLTMRHGTRDGKYHLRFRVYDR--KHTQVDVPANVTVTVREITHEAVTSSGSIRIAGISDEDFI-RIWDYR-TQDIVKSKIDKLRDKLAQLLA
Am2 EHAQFKLDEDTGMIYMKSGTHDGRYHLRFKVYDR--KHTQTDVPANVTVTVKTIPHEAVLNSGSVRISGITDEDFI-RIWDYK-SQTTSRSKAELFRDKLAHLLN
DN EHPRFKLDEDSGMVTMRAGTREGRYHLRFKVYDR--KHTQTDIPANVTVTVREIPHEAVVNSGSVRLSGISDEDFI-RVWNYR-TQSMSRSKMDRFRDKLADLLN
Pt1 AHPNFELDKDTGLIVMKNVTSGGTYFLRFLVHDR--VHTQ-EVSANVSVTVKEIPEEAIYNSGSIRISGISAEDFV-RVWNWT-ENRQVKSKYHKFRDMIASLTT
Pt2 QHQNFDLNVETGEISMIELISGGKYTLHFTVIDQ--KRNE-EVPATVTVTVKEIPEEAVFSSGSIRIAGHSDEDFI-RIWDWK-NKVQVESKYNKFREILAKLLK
Ct -DVHFRLDRNTGQLFMRGRVTTGEYDFKVRVYDA---LWQREVISTVTVTIQEIGDDAVLSSGSLRLQGMTAEEFVDRPMLQK-TGHFGLSTYDILRREIAKKLS
Lg YKDLFEVISENGSIILKEKVPEGEYTFTVKVTDN---LHQKTVESTVTVVIQMLTEEAVKKSGAMRLTGITAEDFVDREKVD--DG-NLNSYYDSKSDKFRQLL-
LvG LPDFFQLDSDTGDITMAEGTPAGVYEMDIRVSDDG---TYESVVSTVIVTVKDIPREAVFSSGSIRFSGTTAEELI-TP--DS-EG---VSNLDRLKVILANAVG
Bf EPKYFWLNESSGMAYIREKTPAGRHTFSVRVSDG----IWPDAVSSLVVNVKEVTEEQLVNSGSIRFDGTTAEEFI-TP--GK-DG---QSKYDLFRQAIAKVVG
Pn PTRLFNLNQSSGQLSIREGTPPGSYHLQVRVSDS----TWPDVTSTAQVDVTELHHVALQRAASIRLSNLTVEEFFSSS--GGGEKSRF-SRLGLTLAELLQTLP
Mm5 --
2,420 2,430 2,440 2,450 2,460 2,470 2,480 CE1 2,490 2,500 2,510 2,520
Pt1 TRKDN--VDIFSVILK--QERPPITDVRFSAHRTPYFKASMLNGIVALNRELIEQEVGINITMVGIDECFFE-NINC--ETSCTNILMVDR-QPIVVNANRTSFV
DE TSVSN--VDVFTVLQNENHT----LDVRFSAHGSPYYAPEKLNGIVAQNQQRLENELDLQMLMVNIDECLIE-KFKC--EESCTNELHKSS-VPYMIYSNTTSFV
Tc1 TSIEN--VDVFTVMPSHNST---FVDVRFSAHGSPYYAPERLNNKVTVEQEALERELGAEIIMISINECLQPD--VCL-NGSCSNILQIRD-DPAVVFTNKTSFV
Am1 TSVEN--VDVFTVLHSPHHNNESLLDVRFSAHGSPYYAPEKLNTILAQHAKEIERELKTDILLVNIDECLFE-KQYC--NNSCRSYLNAST-VPYPVYTNTSTFV
Ap1 ATVEN--VDVFTILHSPHNPNTTQLDVRFSAHGSPYYQPERINAAIDKHQQELEKELGVTFLMVNIDECLVE-KLYC--ESSCTNFLNKSN-EPAAVYTNTTSFV
Dp1 AKEEN--VDVFTVMSS-SPTRNHSVDVRYSAHGSPYYDPVKLNGLTAAHQDELERILDAEVLMFGIDECLHEKNSICS-EGSCTNRLQITE-QPYRVATNTSTFV
Le1 ELPEN--VDIFYLQDVE-RRGFSGVDVRFNIHGSPYYTSSHVNGLLMQHKDEVENNLGIKIPVIDDNLCLYEDFQSCN-GTSCQQELRAENNNPLVLESETSTIV
Sm1 T--ND--VEIFTVMN---SNRPGMLDVRFAARGSPYYKPEKMNGLLNQHSSEIESELDIKLEMIGIDECLYE-FYNC--ESSCSNELVIYD-KPYKVSTKNTALV
Sm2 VAKDN--VDVFTVMMK--QDRPPITDVRFSVHGSPYLKASKLDGLVAQHREEIEAEVGINITMVGIDECMIEH-QEC--EDSCSNELRVSP-QPYMVNSNKTALV
Cm TKIEN--VDVFSVQLR--NERPPVTDIRFSAHGSPYYHPIKLNGLVLQHRAEIEAEIGINITMVGIDECLYE-NVAC--EGSCTNHLVIST-LPYMVNANKTSLV
Le2 TNIGN--VDVFSVQPY--QSRPPITDVRFSAHGSPYYQPVKLNGLVLQHRAEIESRVGINITMVSIDECLYE-NKAC--EGSCTNNLVVSS-YPYMVNANRTSFV
Dp2 IDREN--VDIFSVQLR--QQRPPITDVRFSAHGSPYYKPIKLNGIVLQHREEIEAEVGINITMVGINECLKE-NQAC--EGSCTNIMEVST-LPYLINANKTALV
Am2 IDREN--VDVFSVQLR--RIHPPLTDVRFAAHGSTYYKPVRLNGIVLMHREEIEKEVGINITMVGIDECYYE-NEVC---EGSCTNTLDISS-LPYMVNANRTALV
DN TEREN--VDIFSVQLK--RKHPPLTDVRFSAHGSPYYKPVRLNGIVLMHREEIEKDVGINITMVGIDECLYE-NQMC--EGSCTNSLEISP-LPYMVNANKTALV
Pt1 TRKDN--VDIFSVILK--QERPPITDVRFSAHRTPYFKASMLNGIVALNRELIEQEVGINITMVGIDECFFE-NINC--ETSCTNILMVDR-QPIVVNANRTSFV
Pt2 VKKDN--VDIFTVLKH--QDHPPMADIRFSAHGSPYYKASRLDGLIGLHRKNIEQIVGVNITMVNIDECLYEE--KC--EDSCTNYLRVYGDRPATVNANRTSLV
Ct VPTGN--VDIFTVVNH--PTLPRTIDVRYSAHGSPYYQPSRLDGIVNLNKNEFEKILGLKIVMVPIDECVKE---VCE-SGGCSNRLVTTP-DPLLINTNSTSIV
Lg ADKLNTKKEKVEVISV--MNNGEFTDVRYSVSGSPYYDASKSDSVISLNRKEFENKVGIKIGMVPIDECMDE---KF--EGGCYNYLNITE-QPAMVNTNSTSFI
LvG AQLAN--FDIFSVLNV--VGMERTVDIRYAAHGSPYYPADQLDLAALSVSNEIEE-LGLSIAQIPVDLCVKE---NVC--ESSCTNVLVV-DPTPTVVDSGTASLV
Bf AQVQN--VDIFSVLNV---PSRRSVDVRYTAHGSPVYPAHKLNGLAAQNRQTLEELVGLTVAEVNVDPCVQE---IC-QTGGCSSRLETSD-TPTLVNSGSASFV
Pn ENVQI--FSVGDASKH----GGRALNVWFAAHGSPYYKAEKLHGYVAANKAKLEAILGVSISQVGVDDCPYTD---CSQSGGCSNEVSFSQ-APVALSSGNISLV
Mm5 --

```
                 2,530     2,540     2,550     2,560     2,570     2,580  LG1  2,590     2,600     2,610     2,620
Pt1  GVNTWVQPKCVCGARDF-SEIE--TCRKR-PLPCHNGGRCFESYGG-VSCK-CLDGYE-GPQCQMTTRSFGGRGWAWFPSL-QQCENSHLSLEFMTRKPDGLLLY
DE   GVNAFVQAQCVCEAPLM--------------RRCLNGGS--PRYGENDVCD-CIDGFT-GPHCELVSVAFYGSGYAFYEPI-AACNNTKISLEITPQIDQGLIMY
Tc1  GVKAVAEAVCECRKHMT--------------KECFNGGT--PIEG---ICN-CPEGFT-GPNCESLGISFAGDGWAMYPPF-DSCNNTLISFSVLPQTDDGLIFY
Am1  GVRAIVDPQCTCHVYEP--------------IVCLNGGT--PLAD---RCE-CPPGLE-GPRCELLGIGFHGDGWAVMPPPGQACDDSHLGLEITPHMDNGLVFY
Ap1  GIRAVIDPFCQCEQRAT-TK-----------ITCHNGGT--PTSDGTV-CQ-CPSGFE-GPRCEILSIGFYGEGYALYPSL-QSCMESHMSLEVRTSVDNGLLFY
Dp1  GVDARIVPECVCLADEY-LSSS--TCS---ATSCLNGGTC--TGDSVQPCR-CPDGFQ-GPRCEGLDVSFGGTGWAWYDPL-PTCASGFLSLTVVTQTGNGLILY
Le1  GVRVTEKYTCNCGYLDP-QPRA---CPEGFENYCYNGGVCNYTSAGGLSCT-CLDDYNYGPRCELLTGRFK-NGFAWFDTL-GTCEEPTLHITFQSDSSSAVLLY
Sm1  GIRAFVKHNCVCAAREN-FHSE-TMCK---WNSCYNRGVCKVEHNG-IKCK-CESGFD-GPRCQQTTRSFNGDGYAWFSPL-ALCENSHLSFEFITKDGDGLIFY
Sm2  GVRAFVVRVCKCKARDF-EVRE--NCN---TVVCFNGGRCVENPGG-VVCE-CPRGFD-GPRCQQTTRSFRGNGWAWFNSL-EQCENSHLSLEILTKEQSGLIFY
Cm   GVRTEVVPDCTCGARNF-STEE--SCR---PNPCYNGGRCIEGKSG-IRCK-CPEGYD-GPRCQMLTRTFRGNGFAWFQPL-AMCDNSHISLEFLTRRPEGLLFY
Le2  GVRTQVIPECTCGARNF-PREE--VCR---PNPCFNGGQCTASKSG-VRCK-CPPGFD-GPRCQMTTRSFGGDGFAWFPPI-KMCDNSHLSLEFLTAKKDGLIFY
Dp2  GVRIDIQPECTCGARNF-TRDE--SCR---NNPCHNGGRCVEGRYG-VTCQ-CPSGYD-GPRCQQTTRTFRGSGWAWYPAL-EMCENSHLSIEFLAKKPDGTLLY
Am2  GVRVDVIAECTCGARNF-SKGE--SCR---SSPCYNGGRCVEGRFG-LSCQ-CPAGYN-GPRCQQTARSFRGNGWAWYPAL-EMCDNSHLSFEFITKKPDGLLLY
DN   GVRVDTIADCTCGARNF-TKPE--SCR---TTPCHNGGRCVDTRFG-PHCS-CPVGYT-GPRCQQTTRSFRGNGWAWYPPL-EMCDESHLSLEFITRKPDGLIIY
Pt1  GVNTWVQPKCVCGARDF-SEIE--TCRKR-PLPCHNGGRCFESYGG-VSCK-CLDGYE-GPQCQMTTRSFGGRGWAWFPSL-QQCENSHLSLEFMTRKPDGLLLY
Pt2  GVRAQIEAECTCGARDF-TSME--SCKTR-PKPCYNDGICSDKFGV-INCT-CSEGFN-GPQCQKTTRHFGGDGFAWFPPL-QQCETSHFKLEFMTQSQNGLLLY
Ct   GVTAYVQADCVCAARTFDESSEGHLCT--PESCYNGGTCNQREAD-FFCS-CPRGFE-GARCQQLRHSFNGNGWAWYKPL-AQCQDSHTSLEMITSHANGLILY
Lg   GVETKVEALEGCRATMYPDEQE---CS---ADYCYNGGECVKDDWNTISCPKCPIGYD-GPRCQGTHHSFDGKSYSLFKPL-EQCLDSQTSIEFITQQADSLIFY
LvG  AITSVLEAQCICGARTVAPG----NCD---SDPCLNGGTCEDVHGGTYRCT-CPYLFD-GPNCQQTKRSFE-NGYASFSTL-RQCEETSLSIEFITEVSSGTLLY
Bf   SVTATMVHECTCPQRAEVRG----LCD---SSPCYNGGTCINTPTG-YRCK-CRNGYD-GIDCQQTKRSFRANGFAWFDPL-PQCLDTRISLEFITKHPDGLLLY
Pn   SLAASLTAQCGCRGREAVHL----SCSAYPVNPCLNGGTCQDGPLG-YRCK-CPPMYD-GPECQQTKHSFGGQGYAWFPPI-KPCYQSHISLEFLAESANGLLLY
Mm5  ---------------------------------------------------------------------------------------------------------
```

```
             2,630     2,640     2,650     2,660     2,670     2,680     2,690     2,700     2,710     2,720     2,730
Pt1  NGPISNPDIGEVAVQDFISLELQSGRPRLLIDFGSGTAEIVVY--------VDESLSDGEWHQIDIFWDRETVRLVIDNCQKAKI---EDSD--PPKINRSRCET
DE   LGPLNFNPL--LAISDFLALELDNGYPVLTVDYGSGAIRIRHQH--------IKMV-ADRTYQLDIILQRTSIEMTVDNCRL-------------------STCQT
Tc1  VGPLSKRQA--ELNKDFMSVEMRGGFVVLKVDFGDDTEEFISDY--------KKIN-DGGSHKIKINYTNVEVQIEVDECPS-------------------DCIK
Am1  FGPMTYSPK--MKIQDFMALELQQGYAVLYMDYGTGTVRLDQRH--------IKLT-DGKSHKIDIYFTKNAIEMKVDNCGI-----------------SACIS
Ap1  AGPSSVKPSP-LSVQDFMSLELKDGYPVLLVDYGSGTLKIGQKQ--------IKIS-DGEPHRIEIIWTKTSIEMKVDSCNL-----------------PSCLS
Dp1  AGPTAVPPD--TSVTDFLALELREGSPVLYLDLGSGTRRLELPDR-------SRNLVDGKAHDLEISWNQRSVQMRLGQCSDGQ---------------PYCSA
Le1  NGPVIASPYE-YYPKDFLYIVLNNWVVEAYLNLGSETSRVYVPVD---------EGESEPYDVYLSWTKSTVSLIIPNCGLNITEEASEA-----------CKR
Sm1  NGPIAEPLSSE-LVQDFMSFELEGGKPRLLVDYGSGTAEIIVS--------KSKMLNDSEWHRIDISWEPELVTLTVDLCIDISANVYDGKS---SVTERVRCEA
Sm2  NGPIVDPEPEERIITDFVSLELMNGRPRLLIDFGSGTLELKIE--------SRFSIADGEWHRIDLFWNAELVRMVVDFCKNAYIDDREETAG-PPRFDRSECEA
Cm   NGPITSPETDEIIVSDFIALELEKGRPRLLVDFGSGTLQLRVN--------TKTSLNDGKWHKIDIFWDTENVRIDVDHCVAAEVQEPEDGS--EPIFDSSGCQA
Le2  NGPITNPELDGIIISDFVSLELVDGRPRLLIDFGSGTLELKVN--------TKKPLNNGEWHRIDIFWDTENVRMDVDHCIEAEVSEPEDGN--DPIFDSSECQA
Dp2  NGPMVPPESEETLISDFIALELESGNPRLLIDFGSGTLELKIKLN-----KTTHRLDDGDWHRLDIFWDTENVRMVLDLCRYATIYEPEDGT--ASVFNTSACEV
Am2  NGPIVPPEADEIMVSDFISVELERGIPRLLIDFGSGTLELKVK--------PKRTLDDGEWHRLDIFWNTETVKLIIDYCKSADISEPEDGT--PPEFNDTSCQA
DN   NGPIVPPERDETLISDFIALELERGYPRLLIDFGSGTLELRVK--------TKKTLDDGEWHRIDLFWDTESIRMVVDFCKSAEIAEMEDGT--PPEFDDMSCQA
Pt1  NGPISNPDIGEVAVQDFISLELQSGRPRLLIDFGSGTAEIVVY--------VDESLSDGEWHQIDIFWDRETVRLVIDNCQKAKI---EDSD--PPKINRSRCET
Pt2  NGPISEADSNEKIVQDFFSLELQNGKPRLLIDFGSGTTEIIID--------TGSSLSDGGWHQLDVFWDRETVRMVVDNCMSARN---QDVD--PPVSNHAMCEG
Ct   NGPSTTP--SDDQDGDFILLELRNGYPYLRINHGSGETQLAIDGRDRQGRVQLPRLNDGHWHRIDVFRAGNEVRLVVDQC-RVASLTETSGN----MADRRACEA
Lg   NGPIGEL--GPDDPTDYISLELVKGYPRLRIDHGTGELSLQIDGRDEQGSVKLQRLDDGKWHHIDIIREGRNVQLIVDRCQDISRNNGIG-------VDDRACRQ
LvG  NGPIFTP--TGDDPIDMILLELIGGKARLTINLGSTDSTDDNL--VLEAPTDDTQLNDNEWHRIDVYRNGRFVEMTVDRCMGVFFAETSS----SSTLDTSSCRV
Bf   TGPVAPL--ATGEPRDFMAVELVSGKPRLTINLG--DGPLQLD------IAVTTTVNDKKWHRLDIIRQGSTTRFMLDRCKNSVITETVGTGRGTSAEDRTGCEA
Pn   NGPLGNVHSGEQE--DFIAIELRNGVPALSINHGSG--TLTLQ------FPPKSTVTDRRWHRLDIISDGKAVQLILDQCGGVAVNELE--------TDESSCRA
Mm5  ---------------------------------------------------------------------------------------------------------
```

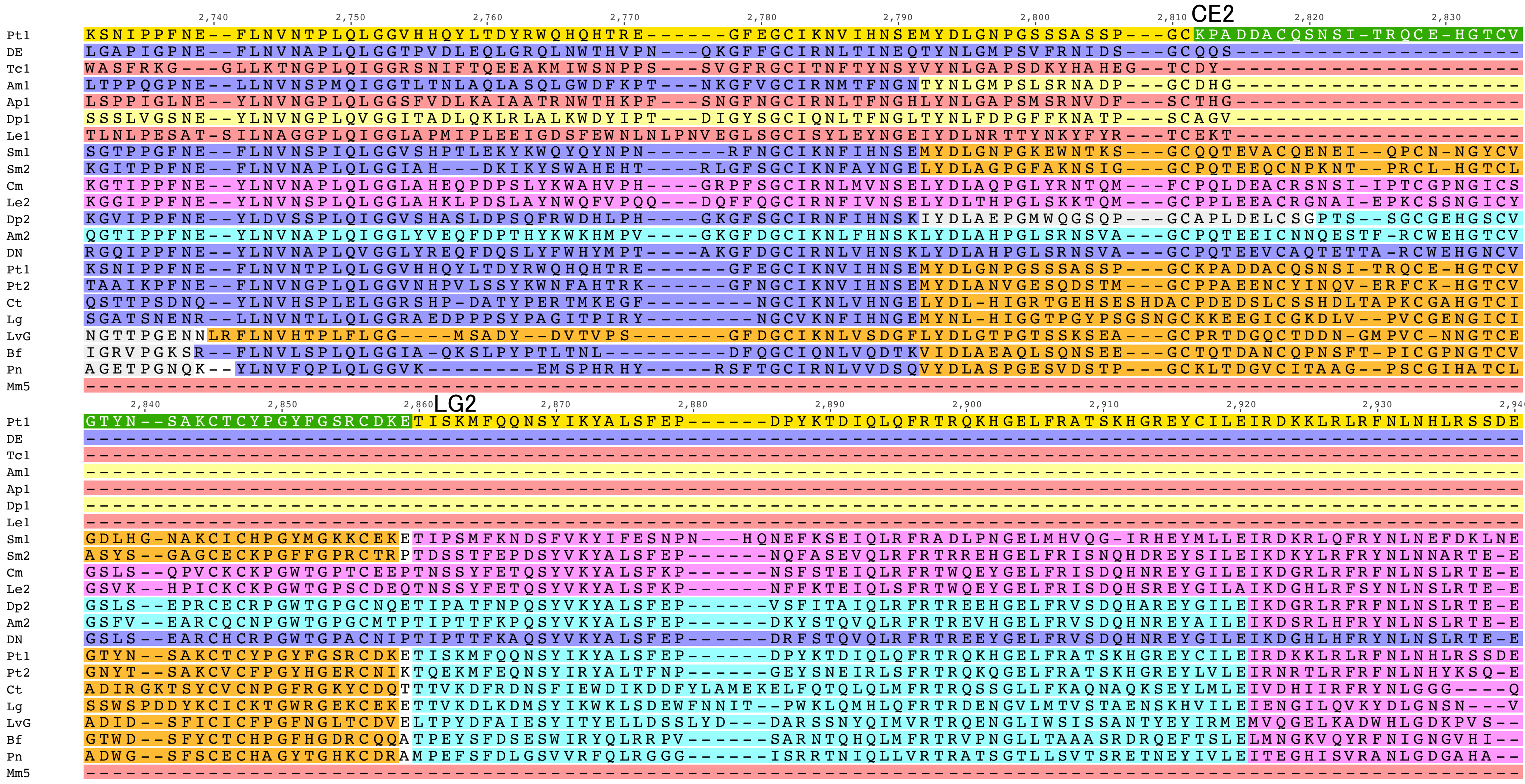

CE2
LG2
2,740
2,750
2,760
2,770
2,780
2,790
2,800
2,810
2,820
2,830
2,840
2,850
2,860
2,870
2,880
2,890
2,900
2,910
2,920
2,930
Pt1
DE
Tc1
Am1
Ap1
Dp1
Le1
Sm1
Sm2
Cm
Le2
Dp2
Am2
DN
Pt1
Pt2
Ct
Lg
LvG
Bf
Pn
Mm5

```
      2,950     2,960     2,970     2,980     2,990     3,000     3,010     3,020     3,030     3,040
Pt1   HELWLPNVQVSDGQWHTVRVLRHGSTASMSVDGGGGRRYNELLEY-EGQHQLMMI-EKQ-NVIAGGDVQYV-GPGVTVVDNDFQEGCMNDIRLDQ--RYLPME--
DE    ---------------------------------------------------------------------------------------------------------
Tc1   ---------------------------------------------------------------------------------------------------------
Am1   ---------------------------------------------------------------------------------------------------------
Ap1   ---------------------------------------------------------------------------------------------------------
Dp1   ---------------------------------------------------------------------------------------------------------
Le1   ---------------------------------------------------------------------------------------------------------
Sm1   HTVYLSSFQANDGYWHTVNVVRYGASASLSVDGGGGKLFNETFNY-NDIHQLITV-PKD-GIVLGGRKDS-----LSGEVPDYENGCMDDVRFDRITQHLPMPPA
Sm2   RELWLSAIIVNDGQWHTVRVARYGSVAYMMLDGGEGRRYNETLDY-EGLHQLMKV-EKQ-NVFAGGYVEYS-GPGMVTVHNDFHTGCMDDIRYDQ--KHLPLPPR
Cm    HDLWLSAVAVNDGIWHSVMVERHGSTATIMLDGGEGRRYNETMTF-SG-HMLMDV-DKQEGVYAGGKAEYT-GIRTFEVYNDYKLGCLDDIRLEG--KHLPLPPG
Le2   HDLWLSAITVNDGQWHSVTVERHGSTAMLMLDGGEGRRFNETVQF-TG-HMLMDV-DKQEGVYAGGKAEYT-GIRTFEVYNDFKLGCLNDIRLEG--KHLPLPPG
Dp2   KDLWLTSITVNDGQWHTVKVSRFGSAAMLELDGGEGRRYNETFIF-EG-HQYMQV-DKQEGVYAGGKAEYT-GVRSYEVYGDYQKGCLDDIRLES--KPLPLPPA
Am2   RDIWLTAIAVDDGQWHTVRVSRYGSAATLELDGGEGRRFNETFSF-EG-HQWLLV-DKQEGVFAGGKAEYT-GVRTFEVYADYQKGCLDDIRLEG--KHLPLPPA
DN    KDLWLNAIVVNDGQWHVVKVNRYGSAATLELDGGEGRRYNETFEF-VG-HQWLLV-DKQEGVYAGGKAEYT-GVRTFEVYADYQKSCLDDIRLEG--KHLPLPPA
Pt1   HELWLPNVQVSDGQWHTVRVLRHGSTASMSVDGGGGRRYNELLEY-EGQHQLMMI-EKQ-NVIAGGDVQYV-GPGVTVVDNDFQEGCMNDIRLDQ--RYLPME--
Pt2   QELWLPDVSVSDGQWHSVKVQRFGSTASISLDGGGGRRYSELLGY-QGFHQELVI-EKQ-NVIAGGDVQYL-APGVTIVDNDFQEGCLDDIRLDS--RYLPME--
Ct    DIVQLSHVQVSDGQWHTVYVERIGQWVTLKLDAGEGRYFNETLGQHKG-HLHVRISQR--SLFAGGDVRFPSSNSDPLVDYDYRDSCMNDVRFNGEWMPMDEQEN
Lg    QILQLYYSPASNGRWHTVELERYGKQFILKMDGGEGRNYNYTLGN-PGEYEGINI-QR--IVFSGASVSETSSISVISPSTDLSDTCLNDVRIENGWFPMEMNEN
LvG   --VTMVNFSINDGAWHAINFDRYDSVVTIKIDGGGGVK--EIQNR-ESQYSGLDV-DEN-SLVIGAFVDVNT-------VTDDFMGCMNDPRINHNFLGMEGTND
Bf    --VRLEDFTVDDGEWHVVNVERFGNEVSLKLDGGGGQR--EITIA-LGSHREILI-DQN-SVLVGANVTRA---AEEQNVYNDFIGCINDIRLNE-----EYLPM
Pn    --LRLPGQRVDVGQWVLVSLSRYDNMFTLRLEHGGGSR--EVQAR-LGSRREIVV-HPS-SLMVGNGPDKA-----------DFQGCLRDVRFNG---HVLPLDG
Mm5   ---------------------------------------------------------------------------------------------------------

                          CE3
      3,050     3,060     3,070     3,080     3,090     3,100     3,110     3,120     3,130     3,140     3,150
Pt1   -NGSENAAVLEWRNLINKCPSNNPCQG--IICP-KPFICLDLWMLHECRCPEGFTI-TEDGK--NCTDANECLSD-PCLNGGSCVNLPNGE--GYYCVCPDGFGG
DE    ---------------------------------------------------------------------------------------------------------
Tc1   ---------------------------------------------------------------------------------------------------------
Am1   ---------------------------------------------------------------------------------------------------------
Ap1   ---------------------------------------------------------------------------------------------------------
Dp1   ---------------------------------------------------------------------------------------------------------
Le1   ---------------------------------------------------------------------------------------------------------
Sm1   EFRSDYAEVFNYSHVESGCDPQ-ACLH--VKCP-ENMYCEDLFFNHDCRCSVGFIM-NKMGL---CVDIDECEDIHICENGGTCNNLLGG----FLCHCPPTHTG
Sm2   ENGTEVAAVVDYKHVRSGCPSNNPCVG--FTCP-MPFVCVDLWMLAECRCASGFVM-SADGK--DCEDEDEC-LANPCLNGGICKNLPNGQ--GFLCECSRGYAG
Cm    LNGTQWGQATMFRNLVKNCISQDQCVN--VTCL-APFICKDLWMRHECGCEDGTIL-SKDQQ--ECVDQDEC-LNDPCQNGGTCHNQEPS----YICDCPRGYYG
Le2   LNGTQWGQATMFRHLQRQCTSHDQCIN--VTCA-APFTCKDLWMRHECGCPEGSVF-HRANS--SCVNENECVLWNPCYNGGTCVDLMP------------DEGK
Dp2   LNGTQWGQATTSHGVRRNCPSNNPCSN--VICP-LPFECNDLWMDYECSCGESKVM-TLDGK--GCIDRDEC-LTNPCLNGGICINREP----YYLCDCPDGYFG
Am2   MNGTQWGQATMARNLERNCPSNKPCAN--VICP-EPFECIDLWNEYDCTCGEGRIP-SPDNK--GCMDKNEC-IDYPCLNGGRCINQDPRF--RYRCICPDGFWG
DN    MNGTQWGQATMARNLEKGCPSNKPCSN--VICP-DPFECVDLWNVYECTCGEGRIM-SPDSK--GCMDRNEC-LDMPCMNGATCINLEPRL--RYRCICPDGFWG
Pt1   -NGSENAAVLEWRNLINKCPSNNPCQG--IICP-KPFICLDLWMLHECRCPEGFTI-TEDGK--NCTDANEC-LSDPCLNGGSCVNLPNGE--GYYCVCPDGFGG
Pt2   -NGSQSAIVLDSQNVKSGCPSNNPCLG--VYCP-PPFVCVDLWMIYECTCPKGFIV-TPDRK--DCIDDDECVSQQPCLNGGTCVNLPDGQ--GYECICGAGYFG
Ct    AYSISANFLTRSRNLEDGCES-TACLT--KHCP-FPLTCFNFWRYAECRCPLGQEFREE-LN--ICENINEC-IQNPCYHG-QC-IDQSQ---GFVCDCYNGYSG
Lg    SES-KAATLVRDVNLEDDCLR-NDCEG--VTCTGEFEVCYPLWGIYECRCQDGYKK--GPNG--KCILIPFC-ETDPCISG-RCNVD--------KCECFEGWDG
LvG   YAVATKSAGVTEGCPSDVCDS-DPCPGSILVCT-------DYWRFYECLCPEGQEEVEDDPD--TCMAIIDC-VPNPCANGGTCVEGDPT---GYTCDCPSGYYG
Bf    EGEN-AVAMATTEGIQPGCWDHEECLS--APCV-IPFVCVDLWRGYICRSGH-YPFFIILLLRSLTRVGSSQIAFTPCFHAALSSGSTPEATVLNICLV--GV--
Pn    -QSRDLVTVLERRGVTSGCSS-NACSS--QPCR-SPLRCVDLWRKHQCKCPGSQVTVTDDSGQQRC-APSPC-GLSSCRNGGVCQPLSPDS---YRCRCLEGFRG
Mm5   ----------------------------------------------------------------------------------------------GVCKCNEQGEF
```

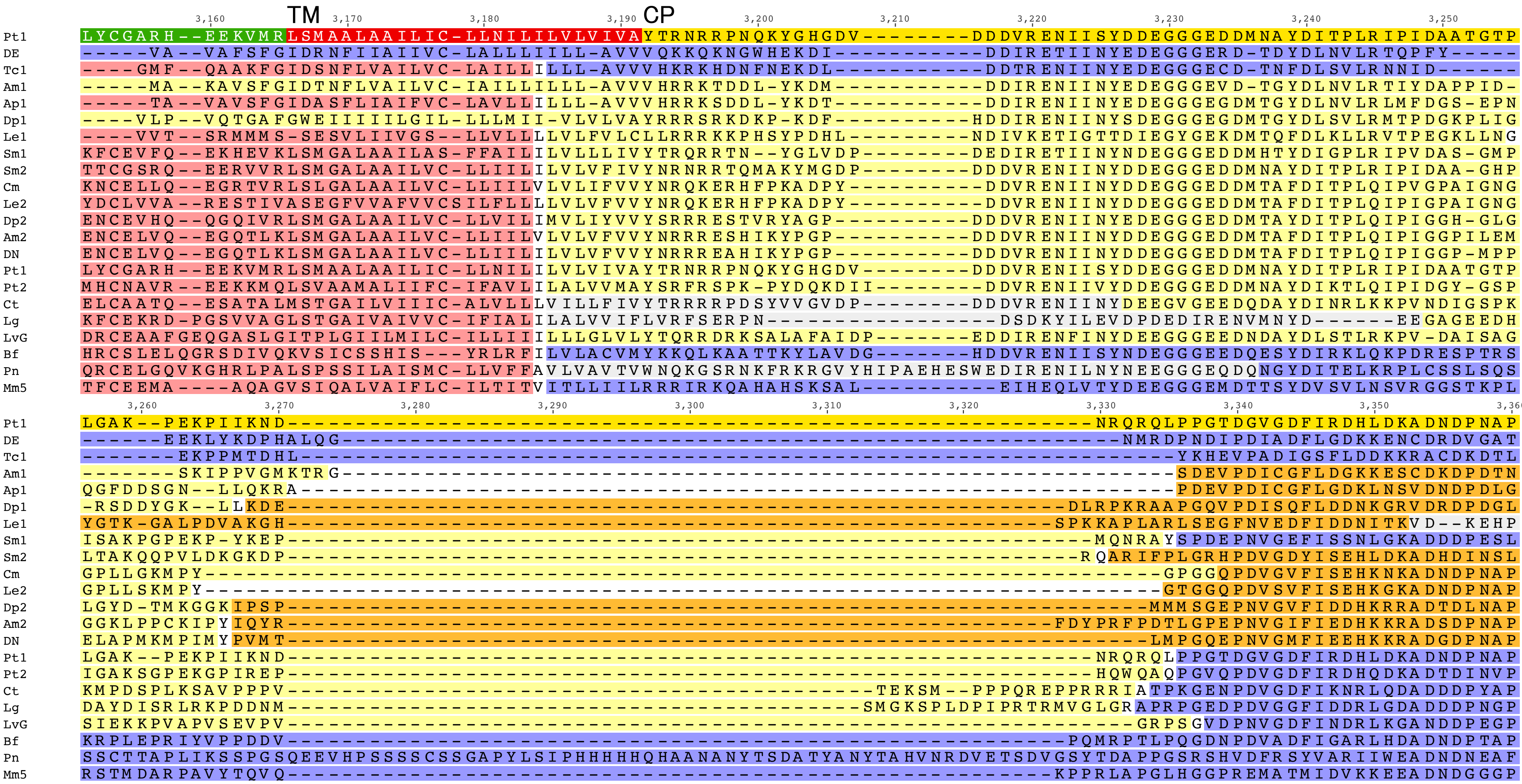
TM
CP
Pt1
DE
Tc1
Am1
Ap1
Dp1
Le1
Sm1
Sm2
Cm
Le2
Dp2
Am2
DN
Pt1
Pt2
Ct
Lg
LvG
Bf
Pn
Mm5

```
      3,370     3,380     3,390     3,400     3,410     3,420     3,430     3,440     3,450     3,460
Pt1  PFDDLRNYAYEGCGSTAGSLSSLASGT-----------------EDNEQDFDY--LNGWGPRFQKLADMYGPGESEED
DE   TVDDVRHYAYEGDGNSDGSLSSLASCT-----------------DDGDLNFDY--LSNFGPRFRKLADMYGEEPSDTDS--------------------------
Tc1  PCDDVRHYGYEGDGNSSGSLSSLASCT-----------------DEGDLKFNY--LSSFGPRFRKLADMYGDDASEEGS--------------------------
Am1  PFDDVRHYAYEGEGNSEGSLSSLASCT-----------------DDGDLKFNY--LSNFGPRFRKLADMYGEEPSDEES--------------------------
Ap1  PYDDVRNYMYEGEGNSVGSLSSLASGT-----------------DDGDLNFDY--LSNFGPRFRKLADMYGEDPSDEESETYRNTHP------------------
Dp1  AYDDLRHYAYEGDGNSMGSLSSLASGT-----------------DDGDLDFEC--LSDFGPRFKKLADMYGDHSSESEDG-------------------------
Le1  DFDDVRHYCFEGDEMSIASLSSLGSGS-----------------YDDDGPYNY--KEDWGPRFNRIAEIYGPKPDEEEDSDYEFPIPKMPPKVVSGGSNPSKLNQ
Sm1  PLDDLRVYAYEGGGSDAGSLSSLASGT-----------------DDNEQDFDY--LNGWGPRFQKLADMYGQGESEEE
Sm2  PFDDLRPYAYEGGGSTAGSLSSLALGT-----------------DDQEQEFDY--LNGWGPRFQKLADMYGPMPAENEDE
Cm   PFDDLRNYAYEGGGSTAGSLSSLASGT-----------------DDNEQDFGY--LNDWGPRFSKLADMYGHGESEEEEDH
Le2  PFDDLRNYAYEGAGSTAGSLSSLASGT-----------------DDNEQDFDY--LNNWGPRFSKLADMYGHGESEEEEDH
Dp2  PYDDLRNYAYEGGGSTAGSLSSLASE-------------------DDEQDFDH--LNGWGTRFQKLADMYGEGDSEEEEEEEEEDIH
Am2  PFDDLRNYAYEGGGSIAGSLSSLASGT-----------------DDEQHEYEY--LGAWGPRFDKLADMYGPAEESEEED
DN   PFDDLRNYAYEGGGSTAGSLSSLASGT-----------------DDEQQEYDY--LGAWGPRFDKLANMYGPEAPNPHNTELEL
Pt1  PFDDLRNYAYEGCGSTAGSLSSLASGT-----------------EDNEQDFDY--LNGWGPRFQKLADMYGPGESEED
Pt2  PFDDLRNYAYEGGGSSAGSLSSLASCY-----------------DDNDHDFEY--LNGWGPRFQKLADMYGQGESEEE
Ct   PHDSVREYAYEGGGSDAGSLSSLASTAS-----------------SEDQEYQY--LNNWGPRFTKLADMYGGQKK
Lg   PPDSVCEYAYEGGGSDAGSLSSLNTTSS-----------------DGDQDYDY--LNDWGPKFAKLADMYGAGQED
LvG  PYDEPHIYDYEGDGSTAGSLSSLNSST------------------DSEQNYDY--LNDWGPQFRKLADMYGS
Bf   PHDSIQPYDYEGQGSTAGSLSSLTSASS-----------------EDDQDYEY--LDNWGPQFRNLADMYSGAGGGASA
Pn   PPDAYHVWCVEGSGSSAGSLSSLGSAVSHRNINTGDGDRDDGEEEEDDGGFVYDRLSRWGPKFQALSEMYDRPQLTLTYRDAMAYIQRSHSHDPLPHHH
Mm5  PYDTLHIYGYEGAESIAESLSSLSTSS--------------------DSDIDYDFLNDWGPRFKMLAELYGSDPQEELII

      3,470     3,480     3,490     3,500     3,510     3,520     3,530    3,539
Pt1
DE   -----------------------------------------NVDDDQGWRI
Tc1  -----------------------------------------------------------------QNGAEESWC
Am1  -------------------------------------------DGVGE-------------------RESESWC
Ap1  ----------------------------------------------------------------------ESWC
Dp1  ------------------------------------------EDGQQGG-----------------QSGSESWC
Le1  ITPSSGASSVGSLNTQNSADGSEPSTVPYTRLGQHNSSSSHEEDGKKGGAPPVVFRGEYHEAVNPLAQSKESWC
Sm1
Sm2
Cm
Le2
Dp2
Am2
DN
Pt1
Pt2
Ct
Lg
LvG
Bf
Pn
Mm5
```
