# Supplementary material for: Evolutionary origin of type IV classical cadherins in arthropods
Source: BMC Evol Biol. 2017 Jun 17;17:142. doi: 10.1186/s12862-017-0991-2 (PMC5473995; doi:10.1186/s12862-017-0991-2)
Supplement: Supplementary file 10 — Conserved cysteine residues in the EC domains of classical cadherins. Alignments were generated from the EC5-EC6 (A), EC7 (B), EC7-EC8 (C), EC13 (D), EC14 (E) and EC17 (F) regions of type III cadherins and the corresponding regions of other classical cadherins. The cysteine residues are shown in red. The “-” character indicates introduced gaps. The classical cadherins shown are as follows: DE-cadherin (DE, fruit fly); Tc1-cadherin (Tc1, beetle); Am1-cadherin (Am1, honey bee); Ap1-cadherin (Ap1, aphid); Dp1-cadherin (Dp1, water flea); Le1-cadherin (Le1, sea slater); Sm1-cadherin (Sm1, centipede); Sm2-cadherin (Sm2, centipede); Cm-cadherin (Cm, shrimp); Le2-cadherin (Le2, sea slater); Dp2-cadherin (Dp2, water flea); Am2-cadherin (Am2, honey bee); DN-cadherin (DN, fruit fly); Pt1-cadherin (Pt1, spider); Pt2-cadherin (Pt2, spider); Ct-cadherin (Ct, polychaete); Lg-cadherin (Lg, snail); LvG-cadherin (LvG, sea urchin); Bf-cadherin (Bf, amphioxus); Pn-cadherin (Pn, fish); and Mm5-cadherin (Mm5, mouse). (PDF 41 kb) [file 12862_2017_991_MOESM10_ESM.pdf]

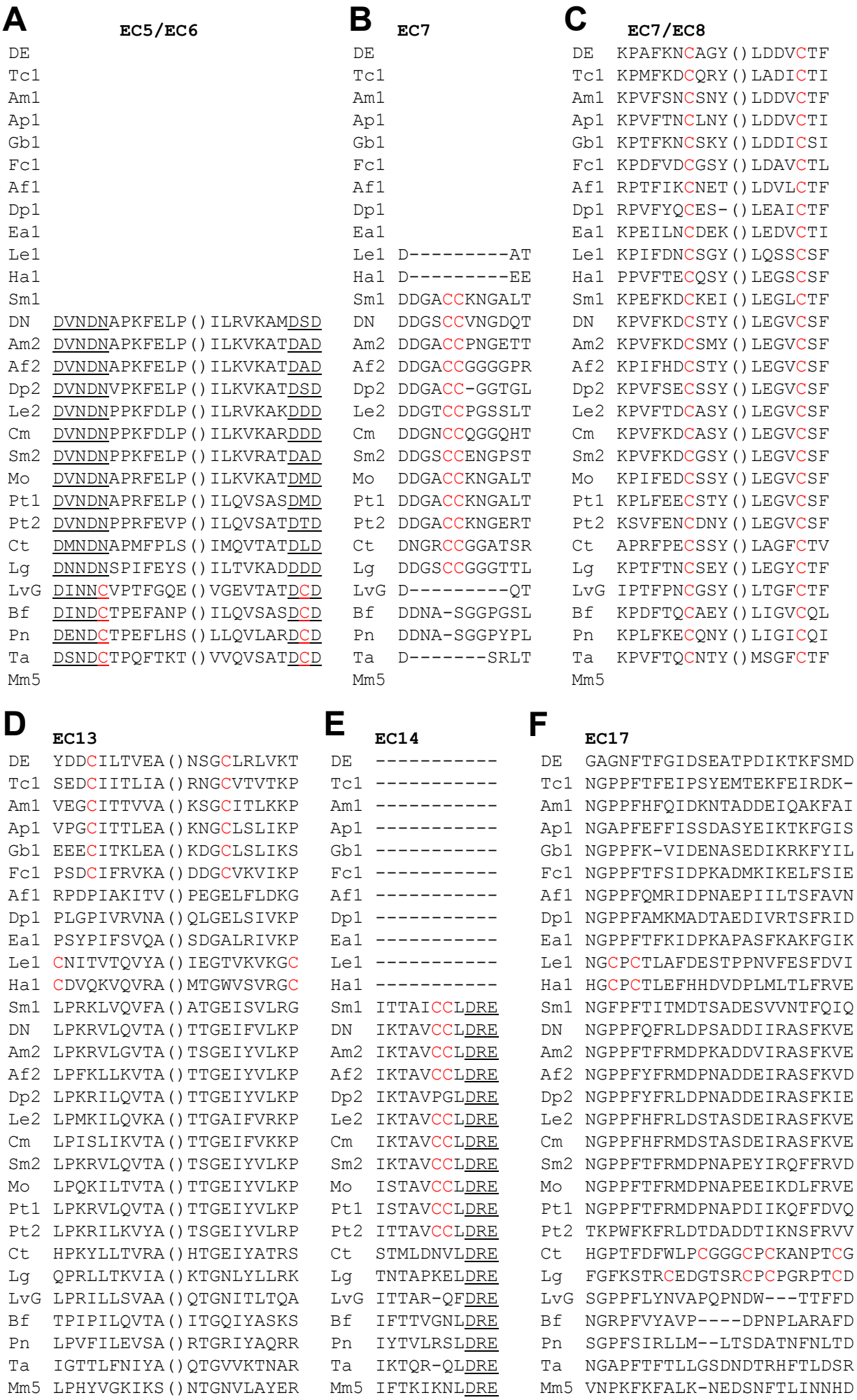
A
EC5/EC6
DE
Tc1
Am1
Ap1
Gb1
Fc1
Af1
Dp1
Ea1
Le1
Ha1
Sm1
DN DVNDNAPKFELP()ILRVKAMDSD
Am2 DVNDNAPKFELP()ILKVKATDAD
Af2 DVNDNAPKFELP()ILKVKATDAD
Dp2 DVNDNVPKFELP()ILKVKATDSD
Le2 DVNDNPPKFDLP()ILRVKAKDDD
Cm DVNDNPPKFDLP()ILKVKARDDD
Sm2 DVNDNPPKFELP()ILKVRATDAD
Mo DVNDNAPRFELP()ILKVKATDMD
Pt1 DVNDNAPRFELP()ILQVSASDMD
Pt2 DVNDNPPRFEVP()ILQVSATDTD
Ct DMNDNAPMFPLS()IMQVTATDLD
Lg DNNDNSPIFEYS()ILTVKADDDD
LvG DINNCVPTFGQE()VGEVTATDCD
Bf DINDCTPEFANP()ILQVSASDCD
Pn DENDCTPEFLHS()LLQVLARDCD
Ta DSNDCTPQFTKT()VVQVSATDCD
Mm5
B
EC7
DE
Tc1
Am1
Ap1
Gb1
Fc1
Af1
Dp1
Ea1
Le1 D---------AT
Ha1 D---------EE
Sm1 DDGACCKNGALT
DN DDGSCCVNGDQT
Am2 DDGACCPNGETT
Af2 DDGACCGGGGPR
Dp2 DDGACC-GGTGL
Le2 DDGTCCPGSSLT
Cm DDGNCCQGGQHT
Sm2 DDGSCCENGPST
Mo DDGACCKNGALT
Pt1 DDGACCKNGALT
Pt2 DDGACCKNGERT
Ct DNGRCCGGATSR
Lg DDGSCCGGGTTL
LvG D---------QT
Bf DDNA-SGGPGSL
Pn DDNA-SGGPYPL
Ta D-------SRLT
Mm5
C
EC7/EC8
DE KPAFKNCAGY()LDDVCTF
Tc1 KPMFKDCQRY()LADICTI
Am1 KPVFSNCSNY()LDDVCTF
Ap1 KPVFTNCLNY()LDDVCTI
Gb1 KPTFKNCSKY()LDDICSI
Fc1 KPDFVDCGSY()LDAVCTL
Af1 RPTFIKCNET()LDVLCTF
Dp1 RPVFYQCES-()LEAICTF
Ea1 KPEILNCDEK()LEDVCTI
Le1 KPIFDNCSGY()LQSSCSF
Ha1 PPVFTECQSY()LEGSCSF
Sm1 KPEFKDCKEI()LEGLCTF
DN KPVFKDCSTY()LEGVCSF
Am2 KPVFKDCSMY()LEGVCSF
Af2 KPIFHDCSTY()LEGVCSF
Dp2 KPVFSECSSY()LEGVCSF
Le2 KPVFTDCASY()LEGVCSF
Cm KPVFKDCASY()LEGVCSF
Sm2 KPVFKDCGSY()LEGVCSF
Mo KPIFEDCSSY()LEGVCSF
Pt1 KPLFEECSTY()LEGVCSF
Pt2 KSVFENCDNY()LEGVCSF
Ct APRFPECSSY()LAGFCTV
Lg KPTFTNCSEY()LEGYCTF
LvG IPTFPNCGSY()LTGFCTF
Bf KPDFTQCAEY()LIGVCQL
Pn KPLFKECQNY()LIGICQI
Ta KPVFTQCNTY()MSGFCTF
Mm5
D
EC13
DE YDDCILTVEA()NSGCLRLVKT
Tc1 SEDCIITLIA()RNGCVTVTKP
Am1 VEGCITTVVA()KSGCITLKKP
Ap1 VPGCITTLEA()KNGCLSLIKP
Gb1 EEECITKLEA()KDGCLSLIKS
Fc1 PSDCIFRVKA()DDGCVKVIKP
Af1 RPDPIAKITV()PEGELFLDKG
Dp1 PLGPIVRVNA()QLGELSIVKP
Ea1 PSYPIFSVQA()SDGALRIVKP
Le1 CNITVTQVYA()IEGTVKVKGC
Ha1 CDVQKVQVRA()MTGWVSVRGC
Sm1 LPRKLVQVFA()ATGEISVLRG
DN LPKRVLQVTA()TTGEIFVLKP
Am2 LPKRVLGVTA()TSGEIYVLKP
Af2 LPFKLLKVTA()TTGEIYVLKP
Dp2 LPKRILQVTA()TTGEIYVLKP
Le2 LPMKILQVKA()TTGAIFVRKP
Cm LPISLIKVTA()TTGEIFVKKP
Sm2 LPKRVLQVTA()TSGEIYVLKP
Mo LPQKILTVTA()TTGEIYVLKP
Pt1 LPKRVLQVTA()TTGEIYVLKP
Pt2 LPKRILKVYA()TSGEIYVLRP
Ct HPKYLLTVRA()HTGEIYATRS
Lg QPRLLTKVIA()KTGNLYLLRK
LvG LPRILLSVAA()QTGNITLTQA
Bf TPIPILQVTA()ITGQIYASKS
Pn LPVFILEVSA()RTGRIYAQRR
Ta IGTTLFNIYA()QTGVVKTNAR
Mm5 LPHYVGKIKS()NTGNVLAYER
E
EC14
DE ----------
Tc1 ----------
Am1 ----------
Ap1 ----------
Gb1 ----------
Fc1 ----------
Af1 ----------
Dp1 ----------
Ea1 ----------
Le1 ----------
Ha1 ----------
Sm1 ITTAICCLDRE
DN IKTAVCCLDRE
Am2 IKTAVCCLDRE
Af2 IKTAVCCLDRE
Dp2 IKTAVPGLDRE
Le2 IKTAVCCLDRE
Cm IKTAVCCLDRE
Sm2 IKTAVCCLDRE
Mo ISTAVCCLDRE
Pt1 ISTAVCCLDRE
Pt2 ITTAVCCLDRE
Ct STMLDNVLDRE
Lg TNTAPKELDRE
LvG ITTAR-QFDRE
Bf IFTTVGNLDRE
Pn IYTVLRSLDRE
Ta IKTQR-QLDRE
Mm5 IFTKIKNLDRE
F
EC17
DE GAGNFTFGIDSEATPDIKTKFSMD
Tc1 NGPPFTFEIPSYEMTEKFEIRDK-
Am1 NGPPFHFQIDKNTADDEIQAKFAI
Ap1 NGAPFEFFISSDASYEIKTKFGIS
Gb1 NGPPFK-VIDENASEDIKRKFYIL
Fc1 NGPPFTFSIDPKADMKIKELFSIE
Af1 NGPPFQMRIDPNAEPIILTSFAVN
Dp1 NGPPFAMKMADTAEDIVRTSFRID
Ea1 NGPPFTFKIDPKAPASFKAKFGIK
Le1 NGCPCTLAFDESTPPNVFESFDVI
Ha1 HGCPCTLEFHHDVDPLMLTLFRVE
Sm1 NGFPFTITMDTSADESVVNTFQIQ
DN NGPPFQFRLDPSADDIIRASFKVE
Am2 NGPPFTFRMDPKADDVIRASFKVE
Af2 NGPPFYFRMDPNADDEIRASFKVD
Dp2 NGPPFYFRLDPNADDEIRASFKIE
Le2 NGPPFHFRLDSTASDEIRASFKVE
Cm NGPPFHFRMDSTASDEIRASFKVE
Sm2 NGPPFTFRMDPNAPEYIRQFFRVD
Mo NGPPFTFRMDPNAPEEIKDLFRVE
Pt1 NGPPFTFRMDPNAPDIIKQFFDVQ
Pt2 TKPWFKFRLDTDADDTIKNSFRVV
Ct HGPTFDFWLPCGGGCPCKANPTCG
Lg FGFKSTRCEDGTSRCPCPGRPTCD
LvG SGPPFLYNVAPQPNDW---TTFFD
Bf NGRPFVYAVP----DPNPLARAFD
Pn SGPFSIRLLM--LTSDATNFNLTD
Ta NGAPFTFTLLGSDNDTRHFTLDSR
Mm5 VNPKFKFALK-NEDSNFTLINNHD
